# Identification of vaccine targets in pathogens and design of a vaccine using computational approaches

**DOI:** 10.1038/s41598-021-96863-x

**Published:** 2021-09-02

**Authors:** Kamal Rawal, Robin Sinha, Bilal Ahmed Abbasi, Amit Chaudhary, Swarsat Kaushik Nath, Priya Kumari, P. Preeti, Devansh Saraf, Shachee Singh, Kartik Mishra, Pranjay Gupta, Astha Mishra, Trapti Sharma, Srijanee Gupta, Prashant Singh, Shriya Sood, Preeti Subramani, Aman Kumar Dubey, Ulrich Strych, Peter J. Hotez, Maria Elena Bottazzi

**Affiliations:** 1 Centre for Computational Biology and Bioinformatics, Amity Institute of Biotechnology, Amity University Uttar Pradesh, Noida, India; 2 Texas Children’s Hospital Center for Vaccine Development, Departments of Pediatrics and Molecular Virology and Microbiology, National School of Tropical Medicine, Baylor College of Medicine, Houston, TX USA; 3 Department of Biology, Baylor University, Waco, TX USA

**Keywords:** Vaccines, Computational biology and bioinformatics

## Abstract

Antigen identification is an important step in the vaccine development process. Computational approaches including deep learning systems can play an important role in the identification of vaccine targets using genomic and proteomic information. Here, we present a new computational system to discover and analyse novel vaccine targets leading to the design of a multi-epitope subunit vaccine candidate. The system incorporates reverse vaccinology and immuno-informatics tools to screen genomic and proteomic datasets of several pathogens such as *Trypanosoma cruzi*, *Plasmodium falciparum*, and *Vibrio cholerae* to identify potential vaccine candidates (PVC). Further, as a case study, we performed a detailed analysis of the genomic and proteomic dataset of *T. cruzi* (CL Brenner and Y strain) to shortlist eight proteins as possible vaccine antigen candidates using properties such as secretory/surface-exposed nature, low transmembrane helix (< 2), essentiality, virulence, antigenic, and non-homology with host/gut flora proteins. Subsequently, highly antigenic and immunogenic MHC class I, MHC class II and B cell epitopes were extracted from top-ranking vaccine targets. The designed vaccine construct containing 24 epitopes, 3 adjuvants, and 4 linkers was analysed for its physicochemical properties using different tools, including docking analysis. Immunological simulation studies suggested significant levels of T-helper, T-cytotoxic cells, and IgG1 will be elicited upon administration of such a putative multi-epitope vaccine construct. The vaccine construct is predicted to be soluble, stable, non-allergenic, non-toxic, and to offer cross-protection against related *Trypanosoma* species and strains. Further, studies are required to validate safety and immunogenicity of the vaccine.

## Introduction

New data-driven approaches, such as reverse vaccinology^1,2^, systems vaccinology^3^, and machine learning^4^, have started to capitalize on the vast amount of omics data available for vaccine design. Several computational studies have analysed genomes or proteomes of individual pathogenic strains or species to predict vaccine candidates^5–10^. In one of these studies, researchers have used the protein–protein interaction dataset and a network biology approach to prioritize vaccine targets for *Borrelia burgdorferi*^11^. Moreover, Goodswen et al.^12^ used a machine learning approach to distinguish between true and false vaccine candidates for eukaryotes including *Caenorhabditis elegans*, *Toxoplasma gondii* and *Plasmodium* sp.^12^.

There are several tools, resources, and databases available in the immuno-informatics domain that have contributed to the development of vaccines in the recent past^13–15^. In 2019, Dalsass et al*.* compared six open-source standalone Reverse Vaccinology (RV) programs designed for bacterial pathogens: NERVE, VaxiJen, Vaxign, Bowman-Heinson, Jenner-predict, and VacSol and tested them on eleven different bacterial proteomes^16^. Several advantages, as well as limitations, have been reported in the existing pipelines or tools. For instance, most of the programs and algorithms have been built around bacterial and prokaryotic systems with only a little work with eukaryotic pathogens, including *Trypanosoma cruzi*. Furthermore, the issue of false-positive predictions remains a challenge. (See “Supplementary Website”).

Despite significant advancements in vaccinology, computational proteomics, machine learning, and reverse vaccinology, finding vaccine candidates, producing them in the laboratory, and confirming their efficacy in animal models remains a complicated undertaking. Thus, there is an urgent need for building pipelines or computational frameworks, to integrate diverse algorithms and databases using a single input and provide meaningful results for researchers working on vaccine development.

In this work, we are introducing an integrated framework that combines immuno-informatics approaches, bioinformatics tools, and supervised machine learning-based tools for vaccine discovery. Here, we rank or classify pathogen proteins based on their propensity to be good vaccine candidates and to design safe and effective multiple epitope vaccine candidates using a set of tools such as PsortB, WoLF PSORT, BLAST, HMMTop, ProtParam, FungalRV, NetCTL, VaxiJen 2.0, or IEDB tools. As a proof of concept, we applied our system to different pathogens including *Mycobacterium tuberculosis*, *Plasmodium vivax, Candida albicans,* and *Influenza A* virus and identified several key vaccine candidates.

Since we have a long-term interest in the development of vaccines against neglected tropical diseases, we performed a detailed analysis of genomic and proteomic datasets of *T. cruzi*, the causative agent of Chagas disease (CD). CD affects an estimated 6.5 million people (healthdata.org), particularly those living in extreme poverty in Latin-America and certain areas of the USA, such as South Texas. An estimated 10,000–20,000 patients succumb to CD annually^17^ and previous studies have reported several issues in the development of a vaccine against CD^18^. Since monovalent vaccines had only partial success, the idea of combining vaccine candidates was proposed^19,20^. Recently, Sanchez Alberti et al. designed Traspain, a chimeric antigen including the N-terminal domain of Cruzipain (Cz), the central region of the Amastigote surface protein 2 and a subdominant region of an inactive trans-sialidase^21^ as a potential vaccine candidate.

Clinical studies, like the BENEFIT trial, have shown limited benefits of therapeutic drugs (i.e., benznidazole) in halting the progression of CD-associated cardiovascular disease^22,23^. Even with available antiparasitic drugs, patients with chronic Chagasic cardiomyopathy (CCC) experience cardiac inflammation and fibrosis leading to heart failure, conduction abnormalities, or sudden death. Several studies have demonstrated that CCC from chronic *T. cruzi* infection in the heart can be controlled by therapeutic vaccines in animal models, but so far, no vaccine has entered human clinical trials^24,25^. Using computational techniques, we not only identified vaccine targets against *T. cruzi* but also designed a putative multi-epitope vaccine along with an in-silico model of immune stimulation that predicts responses associated with protective immunity.

## Methodology

**Figure 1:**
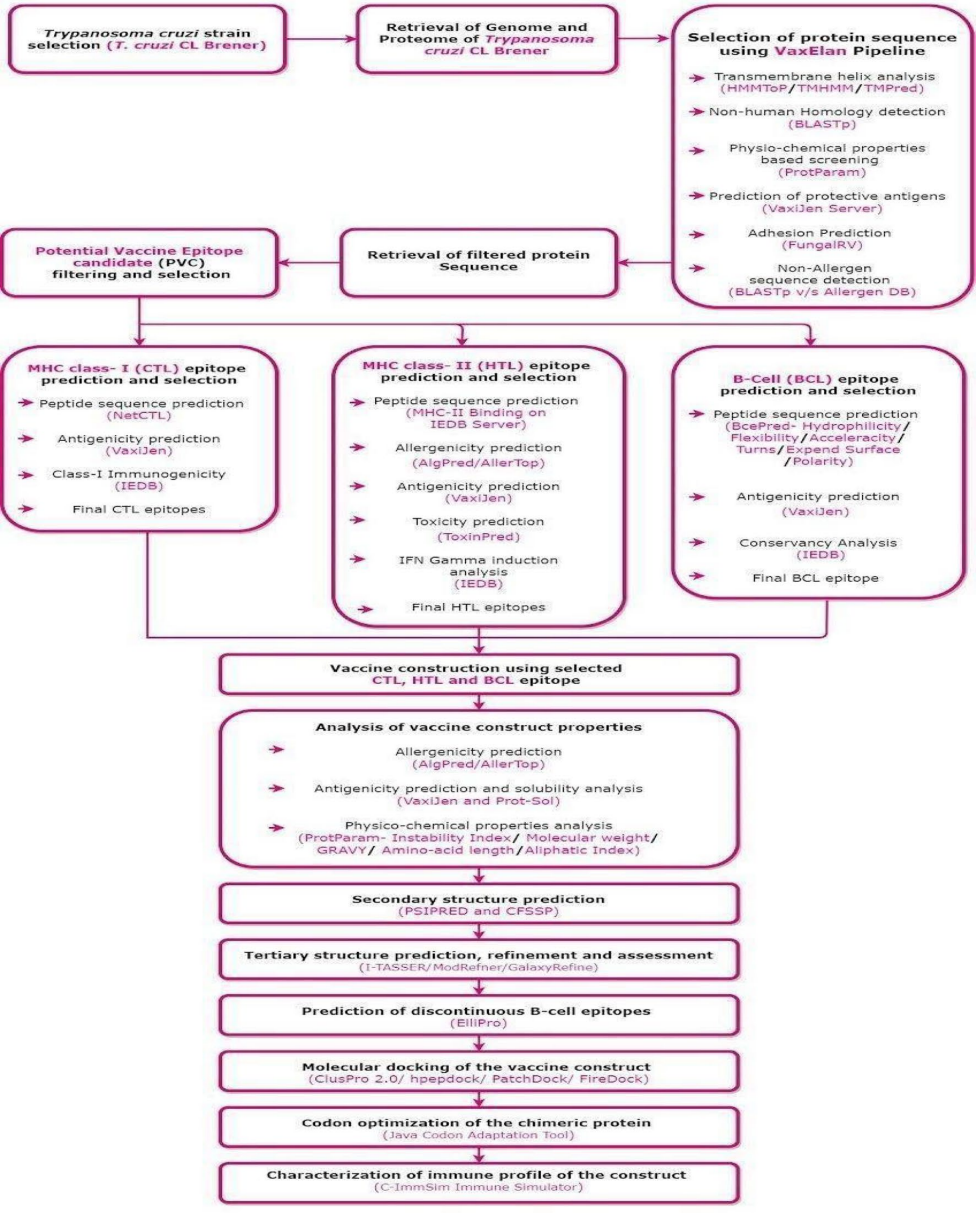
Methodology for developing a multi-epitope subunit vaccine construct (Draw.io—https://www.diagrams.net/-14.6.10).

The Vax-ELAN pipeline (https://vac.kamalrawal.in/vaxelan/) was developed using computational tools to screen the pathogen proteomes. Vax-ELAN evaluates and shortlists proteins that show the relevant characteristics (features) to qualify them as potential vaccine candidates (Supplementary Fig. 1, Fig. 1).

### Features and thresholds

The features used in Vax-ELAN include subcellular localization^26^, secretory/non-secretory protein^27^, stability^28^, cleavage sites^29^, adhesion property^30^, CTL epitope prediction, MHC class-I binding^31^, transmembrane helix prediction^32^, essentiality^33^, virulence^34^, molecular weight^28^, non-homology with host proteins, etc.

In Supplementary Table 1, we summarize various research studies to provide the rationale for the selection of particular features and thresholds. For example, Pizza et al. reported that the main cause of failed cloning and expression of 250 out of 600 vaccine candidates from *Neisseria meningitidis* B was due to the presence of more than one transmembrane spanning region (TM)^6^. Thus, we decided to have no more than two predicted TMs as an a priori requirement. Further, to avoid autoimmunity, the vaccine targets should not be similar to human proteins, therefore BLASTp was utilized to filter those proteins having > 30% identity with human proteins [*E*-value < 0.005]^35^.

Because the immune system readily recognizes surface-exposed proteins on the pathogen, predicting the subcellular localization of the proteins serves as one of the major criteria for designing a vaccine candidate. Therefore, we used tools such as PSORTb2.0, WoLF PSORT, TargetP and CELLO^36,37^ to identify the localization of proteins as extracellular, outer membrane, cytoplasmic, periplasmic, and inner membrane.

### Tools

To compute these features, we used different bioinformatics and immunoinformatics tools/databases such as TargetP^26^, SignalP^27^, ProtParam^28^, PSORTb^38^, WoLF PSORT^39^, TMPred^40^, NetMHC^41^, NetChop^29^, BLAST^42^, Virulence Factor Database [VFdb]^34^ and microbial virulence database [MvirDB]^43^ (Table 1).

**Table 1:** Tools used for extraction of features along with their cut-off values.

S. no.	Features	Tool	Cut-off	References
1	Proteins with less number of trans-membrane helices	TmPred TMHMM HMMtop	≤ 1	Monterrubio-López et al. (2015)^5^ Naz et al. (2019)^44^ Solanki et al. (2018)^45^
2	Non-homology with human	BLAST with human proteome	e-value:10e − 5, identity > 30%, query coverage ≥ 70%	Pearson et al. (2013)^35^
3	Stability (instability index value)	ProtParam	< 40	Solanki et al. (2018)^45^
4	Non-allergen	Blastp with AllerBase	e-value:10e − 5, identity > 30%	Pearson et al. (2013)^35^
5	Adhesion prediction	FungalRv	≥ − 1.2	Monterrubio-López et al. (2015)^5^
6	Essential genes prediction	DEG Database	e-value:10e − 5, identity > 30%, query coverage ≥ 70%	Solanki et al. (2018)^45^
7	Virulence factor	Blastp with VFDB	e-value:10e − 5, identity > 30%	Solanki et al. (2018)^45^
8	Molecular weight	ProtParam	< 110 kDa	Naz et al. (2019)^44^
9	Secretory/non-secretory protein	Signalp (dvalue)	≥ 0.5	Liebenberg et al. (2012)^46^
10	Non-bacterial pathogen/BLAST with gut flora	Blastp with GutfloraDB	e-value:10e − 5, identity > 30%, query coverage ≥ 70%	Naz et al. (2019)^44^
11	Sub-cellular localization	Targetp	≥ 0.8	Goodswen et al. (2014)^47^
12	MHC Class-1 binding (number of high binders)	NetMHC	≥ 4.9	Schroeder and Aebischer (2011)^48^
13	MHC Class-1 binding (number of weak binders)	NetMHC	≥ 5.05	Schroeder and Aebischer (2011)^48^
14	Number of cleavage sites	NetChop	≥ 110	Dhanda et al. (2017)^49^
15	Number of peptides	NetMHC	< 500	Schroeder and Aebischer (2011)^48^
16	Number of amino acids	NetChop	< 500	Dhanda et al. (2017)^49^
17	Cytotoxic T lymphocytes (CTL epitope prediction) (number of MHC ligands)	NetCTL	< 7.5	Solanki et al. (2018)^45^
18	Antigenicity	Vaxijen	> 0.4	Monterrubio-López et al. (2015)^5^
19	Subcellular localization	Psortb	> 9.5	Muruato et al. (2017)^50^
20	MHC Class-1 binding prediction	IEDB (HLA02*01)	> 50 nM	Schroeder and Aebischer (2011)^48^
21	Subcellular localization	Psortb	Cell wall Extracellular	Naz et al. (2019)^44^ Muruato et al. (2017)^50^ Solanki et al. (2018)^45^
22	Subcellular localization	Psortb	Outer membrane, extracellular and periplasmic	Naz et al. (2019)^44^
23	Subcellular localization	Wolf Psort	Extracellular or plasma membrane	Watanabe et al. (2021)^51^

### Strategies

Vax-ELAN has the provision to scan protein sequences (or proteomes) using multiple strategies (See Supplementary Fig. 1). For instance, in strategy 1, we used subcellular localization prediction programs to identify outer membrane and periplasmic proteins. Since, there are no specific algorithms available for protozoa or parasites, we used tools such as PSORTB (Strategy 1A) as well as WoLF PSORTB (Strategy 1B) for the prediction of subcellular localization (See Supplementary Fig. 1).

Subsequently, we employed various filters to prioritize proteins based on features that are associated with antigenicity, including adhesion, allergenicity, and non-homology with the host proteome. The filtering strategy has been reported to find vaccine targets in *Shigella sonnei*^52^, *Brucella* sp.^53^ and *Helicobacter pylori*^54^.

Pearce et al.^55^ had reported the induction of protective immunity against *Schistosoma mansoni* by vaccination with schistosome paramyosin (Sm97), a nonsurface parasite antigen in a mouse model^55^. Therefore, we designed strategy 2 (without sub-cellular localisation filter) in Vax-ELAN, so that there is minimal risk of filtering important (non-surface) antigens.

In another alternative approach based upon inclusion (strategy 4), we use all possible tools (without elimination/filtering) to perform a comprehensive evaluation of a given protein sequence.

In this approach, we also convert the outputs from different tools (N) into binaries (1/0) using threshold values (Supplementary Tables 2, 3). Second, a row-wise sum corresponding to all the properties [i.e., S_i_] was computed. This is followed by the computation of probability value (P_i_ = S_i_/N). Higher P_i_ indicates the propensity of a given protein molecule to possess desirable properties in order to be a good vaccine candidate (Supplementary Table 4).

For instance, trans-sialidases (TS) were found to be among the top-ranking hits (with a comparatively higher P_i_ value of 0.75). TS have been reported to be important vaccine candidates in numerous preclinical immunological studies in TC-CLB (Supplementary Table 5). Likewise, important vaccine targets were reported as top-scoring hits from other pathogens as well. For example, ferric enterobactin receptor protein (S_i_ = 9; P_i_ 0.75) [present in *N. gonorrhoeae*] was shortlisted as a vaccine target^56^ (Supplementary File 1).

In the next section, we describe the approach for building a machine learning-based tool using components of the Vax-ELAN framework.

### Optimisation of thresholds

Though threshold values (listed in Table 1) are supported by literature evidence there is no guarantee of optimality when they are used in machine learning systems. Therefore, we decided to optimise these cut-offs using a quantitative approach. For this reason, we collected protein sequences (antigenic) with experimental evidence from different organisms and labelled them as examples of a positive dataset (see, VaxiDL supplementary). Similarly, another dataset consisting of non-immunogenic proteins (negative dataset) was also compiled. Next, we compared the distributions of each property in the positive and negative datasets. Subsequently, we harnessed Receiver Operating Curves (ROC) to find thresholds at which positive and negative examples could be discriminated against (See Supplementary Fig. 2). With the help of optimized thresholds generated for each property (Supplementary Table 6), we converted the numerical/categorical values of each property into a binary score (0 or 1).

### Machine learning approach

A dataset containing positive and negative protein sequences (PVCs) was compiled using text data mining and manual curation. A total of 11 biological and 1436 physicochemical features were computed for the dataset using several bioinformatics tools. Further, this dataset was subdivided into training, testing, and validation datasets, followed by scaling and normalization of data. Next, a DL model with Fully Connected Layers (FCLs) was constructed, hyper-tuned and trained. The Vaxi-DL model was benchmarked against known PVC prediction tools such as VaxiJen and Vaxign-ML. The preliminary results have shown that the Vaxi-DL model surpassed other PVC-prediction servers in terms of accuracy and efficiency (See, https://vac.kamalrawal.in/vaxidl/). Areas under the receiver operating characteristics curves (AUC) were primarily used to assess the algorithm. On an independent dataset, the algorithm achieved an AUC of 0.90 (95% CI 0.91–0.93) for detecting potential vaccine candidates (Manuscript in Preparation).

### Screening of proteomes of pathogens to shortlist vaccine candidates

Using Vax-ELAN (strategy 4), we screened proteomes of 21 pathogens [seven bacterial, four fungal, five protozoan, and five viral proteomes] to shortlist and rank proteins as potential vaccine targets (Supplementary Table 7). We found that the highest scoring results were enriched in vaccine targets (with experimental evidence reported in the literature) (Supplementary File 1). To illustrate, GPI anchored protein was predicted as one of the top vaccine targets (P_i_ score 0.75) while screening the *Aspergillus fumigatus* proteome^57^. VAX-Elan also predicted Glycerol-3-phosphate acyltransferase (GPAT3) (having P_i_ score 0.71) in *M. pneumoniae*^58^. In addition, Histone 2B^59^ was shortlisted as one of the vaccine targets (P_i_ = 0.67) in *Plasmodium vivax*, and CyRPA^60^ (P_i_ = 0.67) was shortlisted as one of the candidates in *Plasmodium falciparum.* Cysteine protease^61^ (P_i_ = 0.67), 24-c-methyltransferase^62^ (P_i_ = 0.58) and iron superoxide dismutase^63^ (P_i_ = 0.58) in *Leishmania donovani* were found as potential vaccine targets.

### Evaluation of experimentally known antigenic and non-antigenic proteins

**Figure 2:**
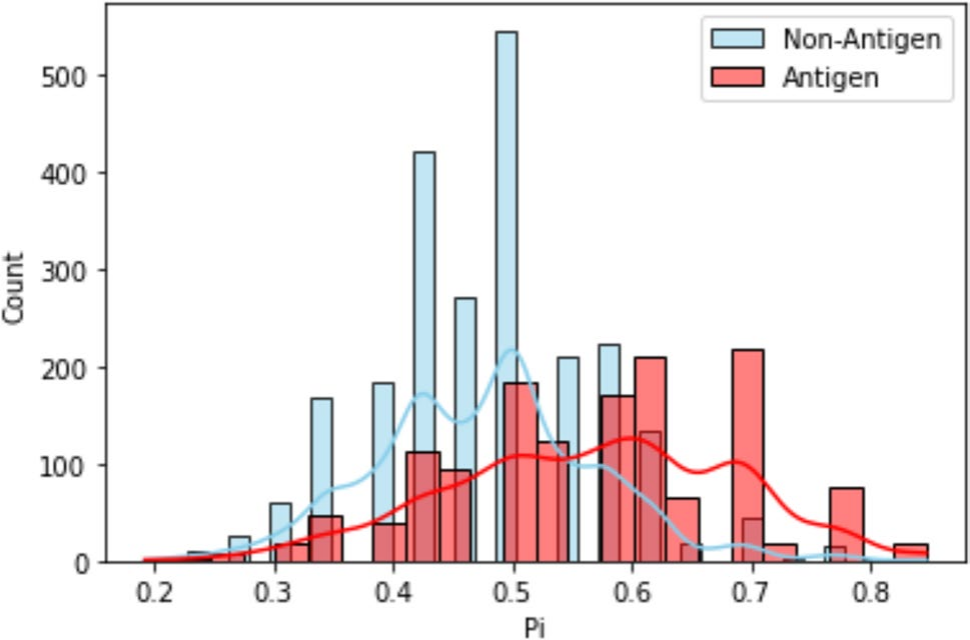
Frequency distribution of P_i_ values based on the results from positive and negative datasets of bacteria, protozoa, fungi, and viruses. The Y-axis represents sequence count. The X-axis represents the *P_i_ score values for each sequence. Blue depicts non-antigen and red antigen sequences. *P_i_ stands for probability value where P_i_ = S_i_/N (where, S_i_ refers to the row-wise sum values and N refers to the total number of the tools).

Protective antigens are proteins that can evoke an adaptive immune response against infectious and non-infectious diseases^64^. To begin with, we collected four datasets of protective antigens belonging to bacteria, protozoa, fungi, and viruses. Each set is composed of antigenic and non-antigenic sequences collected from previously reported resources such as Protegen^65^. For example, we collected 1237 bacterial antigen sequences as a positive dataset (Supplementary File 2). To create a negative/control dataset, we randomly selected those proteins (from the same species) which had less than 10% sequence similarity with sequences belonging to the positive dataset. We also removed redundancies in each dataset by filtering protein sequences that had sequence similarities of more than 30%^36^. Thus, the filtered positive dataset had 670 unique bacterial antigens whereas 677 sequences were obtained for the negative dataset^66^. Similarly, we created independent datasets for protozoan, fungal, and viral pathogens (Supplementary Table 8). Subsequently, we applied the Vax-ELAN tool on sequences of positive and negative datasets (Supplementary Fig. 3a–d, Fig. 2). We found that known antigens had comparatively higher P_i_ values when compared to non-antigens (Mann–Whitney U test, p-value < 0.005).

### Application of Vax-ELAN on *T. cruzi*

#### Retrieval of genome and proteome sequences for vaccine designing

We applied Vax-ELAN on two different strains of *T. cruzi,* CL Brenner and Y. The whole-genome sequences of *T. cruzi* (strains CL Brenner and Y) were obtained from NCBI (Accession ID: NZ_AAHK00000000 and Accession ID: NMZO00000000) along with protein sequences in FASTA format. The results of TC-CLB are shown in the subsequent sections of the manuscript whereas the results of Y strain are shown in the Supplementary File 5.

#### Vax-ELAN pipeline for prediction of vaccine candidates

*T. cruzi* protein sequences were screened based on several parameters such as cellular localization^26^, transmembrane helices^27^, instability index value^28^, allergenicity^67^, antigenicity^66^, the probability of having adhesion-like characteristics^30^, and non-homology with human proteins. Additionally, the *T. cruzi* proteins were also screened against the Database of Essential Genes [DEG]^33^, using the BLAST tool [bit score of 100, cut-off (E-value) of 1E − 5, and BLOSUM 62 matrix]. Further, virulent proteins were extracted using the Virulence Factor Database [VFdb]^34^ and microbial virulence database [MvirDB]^43^. Ideally, the vaccine targets should not be similar to the human proteins, therefore BLASTp was utilized to filter those *T. cruzi* proteins having > 35% identity with human proteins [E-value < 0.005] (See Supplementary Table 9, Supplementary Fig. 4).

#### Alternate strategies adopted for protein filtering

Apart from the methods mentioned in the previous section, we also used alternate strategies to identify potential vaccine targets from *T. cruzi* CL Brenner (TC-CLB). For example, in one of the experiments on proteome screening, we filtered TC-CLB proteins using a set of bioinformatics tools. First, we used the PSORTb tool, to check subcellular localization, followed by the BLASTp tool to evaluate non-homology with human proteins. Subsequently, we used ProtParam to compute the stability of proteins, succeeded by a BLASTp search against the allergen database to filter non-allergen proteins. Furthermore, we used the VaxiJen2.0 server^66^ to check the antigenicity of the filtered set of proteins and then used FungalRV^30^ to predict adhesion molecule-like properties. This strategy generated a set of potential vaccine candidates. As an alternative strategy (1B), we used the WoLF PSORT tool for screening in the first step instead of PSORTb. Additionally, we repeated this analysis after the randomizing order of the application of filters (See Supplementary File-A).

#### Conversion of proteins’ feature/property values into binary values

A row-wise sum corresponding to all the properties [i.e., total score] was computed for TC-CLB. Thereafter, all the proteins of TC-CLB were ranked according to the total score (S_i_ or P_i_). Finally, the top 100 unique proteins were selected for further analysis [See, Supplementary File-A (Strategy-4)].

#### Strategy—ORF-based screening of TC-CLB

To perform comprehensive screening for all possible vaccine candidates, we downloaded the *T. cruzi* CL Brenner and TC-Y genomes from NCBI to find out all possible ORFs. We used Prodigal^68^ to predict 121,349 in the genome. Next, the predicted ORFs were subjected to evaluation with tools such as WoLF PSORT/PSORTB, BLAST, ProtParam, Vaxijen, and Fungal RV to filter proteins.

#### Comparison of different strategies to find top ranking proteins

We collected the top-ranking hits from different strategies and used python-based programs to find common and unique proteins (See, Supplementary File-B). Shortlisted proteins reported from multiple strategies were used in subsequent steps such as epitope prediction and vaccine construction.

#### Interspecies and inter-strain comparison of trypanosoma

We retrieved proteomes from thirteen strains of *T. cruzi* (See Table 2) and four related species of Trypanosoma (See Supplementary File 3). Subsequently, we applied the Vax-ELAN server to obtain top-ranking hits using strategy 4.

**Table 2:** Different strains and species of *Trypanosoma* used for the identification of key vaccine candidates.

*Trypanosoma cruzi* (different strains)	Trypanosoma species with its strain
*Trypanosoma cruzi* Berenice	*Trypanosoma brucei brucei* (927/4 GUTat10.1)
*Trypanosoma cruzi* BrazilcloneA4	*Trypanosoma brucei equiperdum*(IVM-t1***)***
*Trypanosoma cruzi* Dm28c Dm28c	*Trypanosoma brucei gambiense* (MHOM/CI/86/DAL972)
*Trypanosoma cruzi* G	*Trypanosoma congolense* (strain IL3000)
*Trypanosoma cruzi* Sylvio_X10_1	
*Trypanosoma cruzi* Marinkellei B7	
*Trypanosoma cruzi* YcloneC6	
*Trypanosoma cruzi* CL	
*Trypanosoma cruzi* CL Brenner	
*Trypanosoma cruzi* CruziDm28c	
*Trypanosoma cruzi* Dm28c	
*Trypanosoma cruzi* TCC	
*Trypanosoma cruzi* Y	

### Design of multi-antigenic and multi-epitope vaccines against TC-CLB

#### Identification of epitopes

Numerous studies have suggested that epitope-based antigens can induce protective immunity against different infectious agents^69–71^. Various methods have been described in the literature to determine the B and T-cell epitopes which include; functional assays wherein the antigen is sometimes mutated and antibody-antigen interaction is evaluated, 3D structure analysis of antigen–antibody complexes or screening the peptide library of antibody binding, utilization of MHC multimers, and lymphoproliferation by ELISPOT assays^72^. Apart from these time-consuming and expensive experimental techniques, scores of computational methods have also been developed in the past few years. In the subsequent section, we shall describe different approaches for the prediction of B-cell, MHC-I, and MHC-II epitopes in potential vaccine candidates.

#### Selection of linear B-cell epitopes

**Figure 3:**
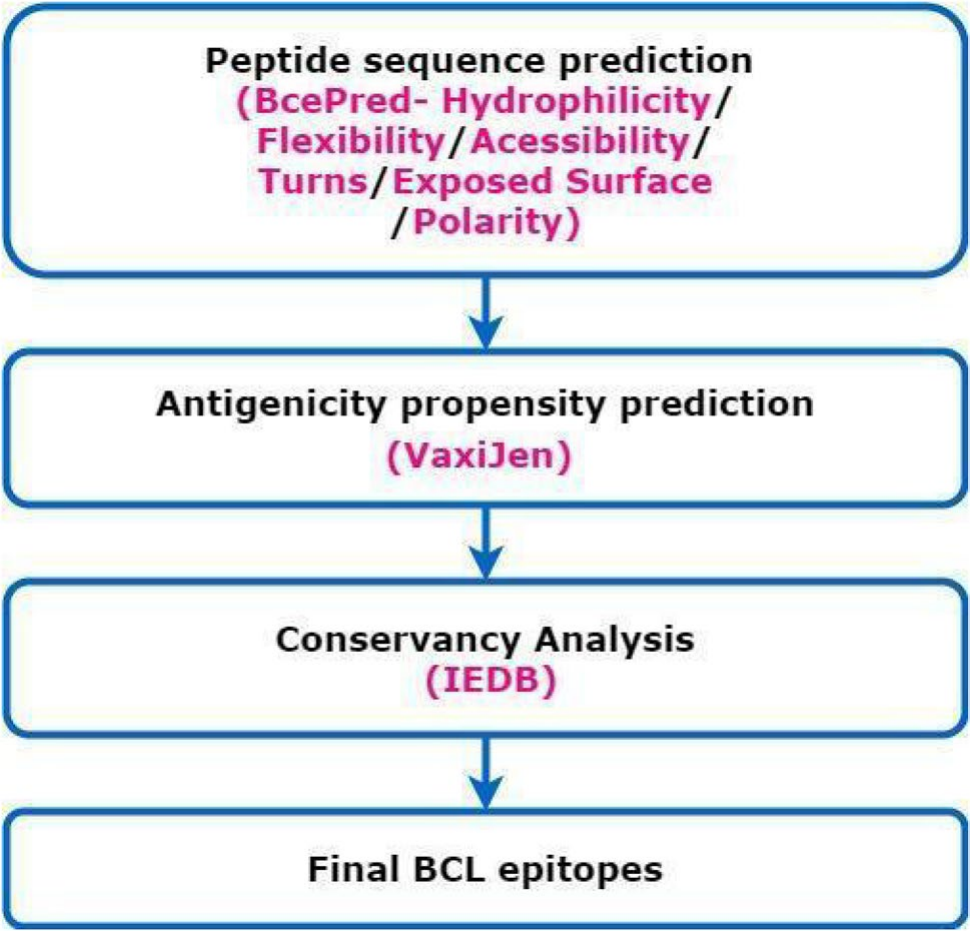
Workflow for the selection of B-cell epitope sequences (Draw.io—https://www.diagrams.net/-14.6.10).

Linear B-cell epitopes are effective antigenic peptide sequences for stimulating B-cell immune responses. There are different methods for B-cell epitope predictions which can be classified into sequence-based and machine learning-based methods. We used multiple tools for predictions which include BCEPRED^73^, ABCPred^74^, and BepiPred^75^ servers (See, Supplementary File-C). We selected the top-scoring epitopes simultaneously predicted by different servers for the final vaccine peptide. Besides, we also used VaxiJen 2.0 along with the IEDB server conservancy analysis to rank and shortlist epitopes. To illustrate, only those epitopes which had shown 100% conservation were selected (Fig. 3).

#### T-cell epitope prediction

The objective of T-cell epitope prediction is to identify short peptide sequence within an antigen that can act as a stimulant of CD4^+^ or CD8^+^ T-cells. There are several methods available to predict MHC binding peptides which can be divided into data-driven approaches or structure-based methods. Structure-based methods are not used commonly because of their poor accuracy and requirement of intensive computational infrastructure. Data-driven methods are based on peptides [i.e., anchor residues, PSSM] known to bind with MHC molecules which are stored in databases such as IEDB, EPIMHC^76^, and AntiJen^77^. Further, there are machine learning-based methods that have been trained on data sets consisting of peptides that either bind or do not bind to MHC molecules. The presence of hundreds of allelic variants of human leukocyte antigens [HLAs] encoding MHCs presents another set of challenges for epitope prediction^78^. We used different methods such as NetCTL^79^, Propred^80^, EpiJen^78^ and NetMHC^81^ tools. Different tools for predictions were used during the study but for brevity, we shall describe results from one of the best-known tools (i.e., NetCTL) in subsequent sections.

#### Selection of cytotoxic T lymphocytes [CTL] epitopes

**Figure 4:**
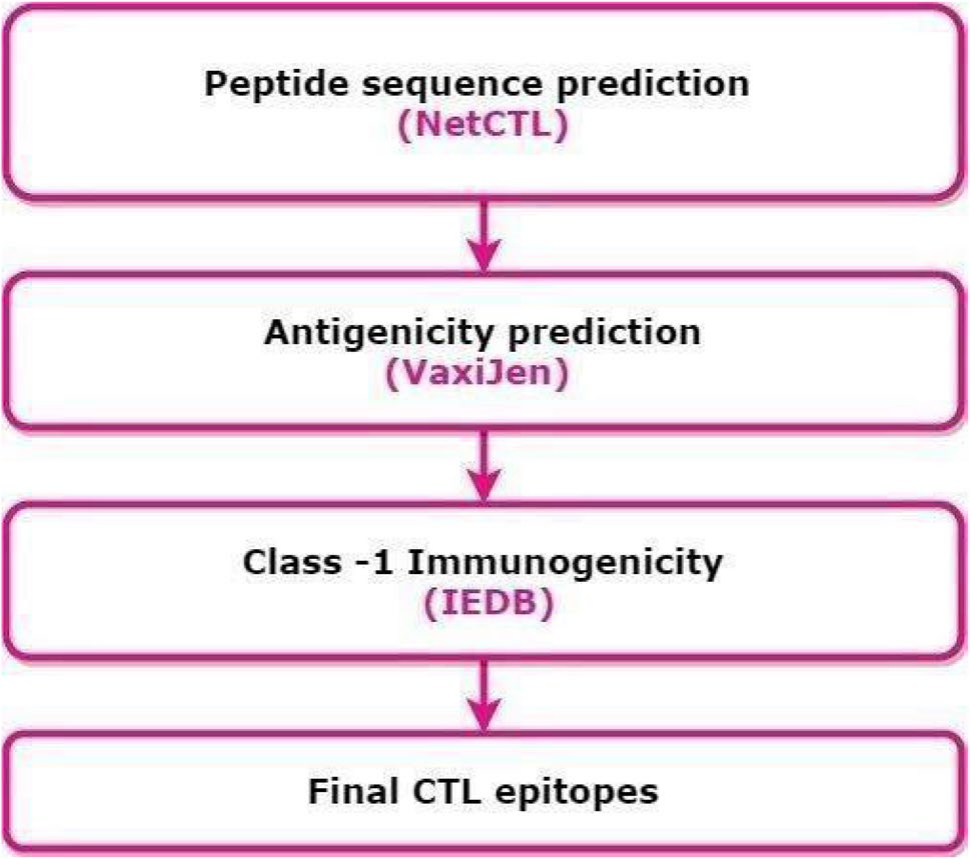
Workflow for selecting cytotoxic-T-lymphocyte epitope sequences (Draw.io—https://www.diagrams.net/-14.6.10).

NetCTL1.2 server has demonstrated comparatively high-level accuracy for CTL epitope predictions therefore a docker image of this tool was created for its execution on local systems (See, Supplementary File-D). It predicts the MHC-class I binding peptide sequences, with proteasome C-terminal cleavage and transporter associated with TAP efficiency (Transporter associated with Antigen Processing). Using this server, the CTL epitopes were predicted based on default parameters and cut-offs [MHC supertype A1, the threshold as 0.75, and weight on C-terminal cleavage as 0.15, and weight on TAP transport efficiency as 0.05]. Further, these epitopes were subjected to antigenic propensity analysis using the VaxiJen 2.0 and immunogenicity analysis (by IEDB class-1 Immunogenicity servers). The epitopes showing poor scores, or overlaps were discarded (Fig. 4).

#### Selection of helper T cells [HTL] epitopes

**Figure 5:**
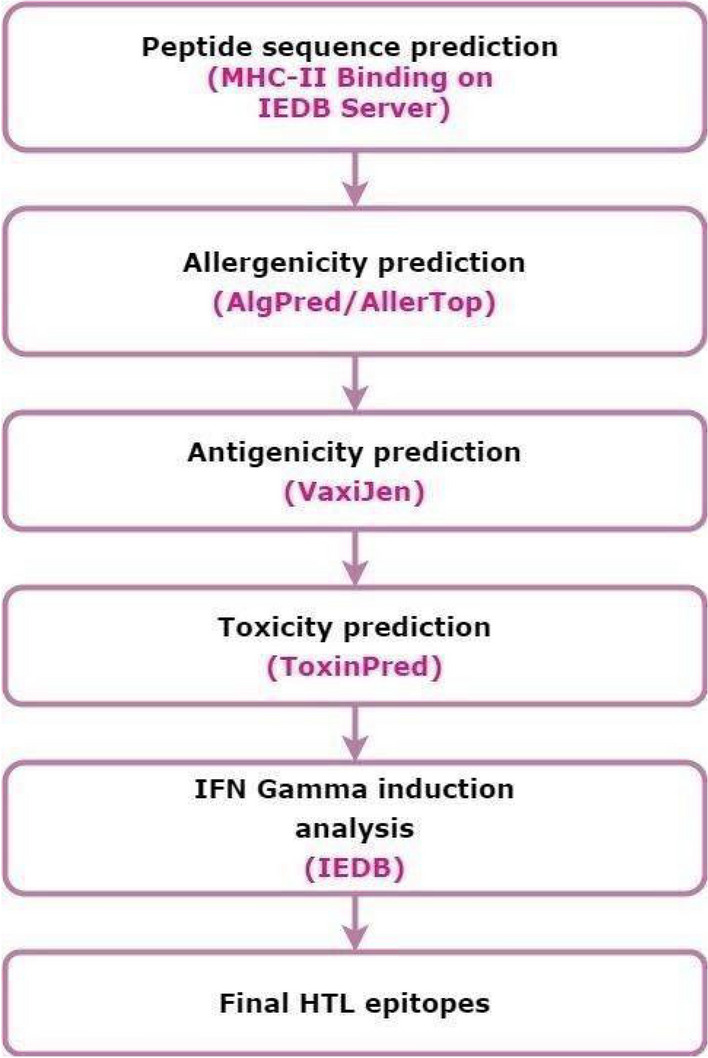
Workflow for selecting Helper-T-lymphocyte epitope sequences (Draw.io—https://www.diagrams.net/-14.6.10).

Prediction of HTL epitopes was performed using the IEDB-MHC-II binding tool (http://tools.iedb.org/mhcii/). This tool utilizes different methods to predict the epitopes, including a consensus method combining NN-align, SMM-align, and other combinatorial approaches. Epitopes obtained through the MHC-II Binding server were subjected to allergenicity prediction using the AlgPred^82^ and AllerTop^83^ servers. Next, using the VaxiJen 2.0 server, non-allergenic epitopes were tested for their antigenic propensity. To predict the toxicity status of epitopes, the antigenic epitopes were subjected to the ToxinPred server^84^. Finally, by employing the IFNepitope server^15^, IFN gamma induction analysis was performed on the non-toxic epitopes. Epitopes that possess the potential to induce the release of IFN gamma were selected as potential epitope candidates for vaccine construction (Fig. 5) (See, Supplementary File-E).

#### The assemblage of multi-epitope vaccine candidate sequence

Three potential vaccine candidates were constructed from top-ranking B-Cell, CTL, and HTL epitopes predicted using various bioinformatics tools. Immunogenicity of the constructs was enhanced by adding adjuvants such as β-defensin [Accession ID: AGV15514.1], L7/L12 50s ribosomal protein [Accession ID: WP_088359560.1, *Flavobacteria* JJC], and HABA protein [Accession ID: AGV15514.1; *Mycobacterium. tuberculosis*]. The adjuvant was attached to the first top CTL epitope [Protein ID: XP_804513.1] using an EAAAK linker. The other top CTL epitopes, belonging to the eight proteins filtered using the RV pipeline, were joined with each other through the GGGS linker. Next, the AAY linker was used to connect the CTL epitope to the HTL epitope sequence as well as all the HTL epitopes with each other. The KK linker was used to bridge the HTL epitope to the BCL epitopes as well as the BCL epitopes with each other. Finally, an EAAAK linker was added at the end to improve the stability of the constructs.

#### Evaluation of antigenicity and allergenicity of vaccine construct

The antigenic propensity prediction for the vaccine construct was performed through VaxiJen 2.0 and ANTIGENpro (http://scratch.proteomics.ics.uci.edu/) servers. The VaxiJen tool is based on the principle of auto cross-covariance [ACC] transformation of protein sequences into vectors using the physicochemical properties of amino acids.

The AlgPred and AllerTOP (http://www.ddg-pharmfac.net/AllerTOP) servers were used to predict the allergenicity of vaccine constructs. AlgPred is a web-based tool for predictions of allergens that combines bioinformatics and machine learning approaches such as IgE epitope scanning, MEME/ MAST motif-based search, amino acid composition, or dipeptide composition-based SVM methods, hybrid method, and BLAST on ARPs. The authors have reported an accuracy of 93.5% for their tool. On the other hand, AllerTOP v2.0 is based on auto and cross-variance transformation, amino acid E-descriptors, and machine learning methods such as k-nearest neighbours [KNN], algorithm. AllerTOP v2.0 was reported with 85.3% accuracy at fivefold cross-validation.

#### Analysis of solubility and physicochemical properties

To evaluate the solubility of the designed vaccine sequence, Protein-Sol^85^ [https://protein-sol.manchester.ac.uk/] server was used. Furthermore, it was assessed for several physicochemical parameters by using the ProtParam server. The properties evaluated include molecular weight, theoretical isoelectric point [pI], half-life, instability index [II], aliphatic index, and hydropathicity or GRAVY value.

#### Prediction of the secondary structure of the construct

PSIPRED^86^ and CFSSP^87^ tools were employed for secondary structure analysis. The consensus of both tools was taken into consideration. PSIPRED 3.2 is a freely accessible online server that utilizes a position-specific iterated BLAST for the identification and selection of specific sequences that show significant similarity with the designed vaccine construct. Further, it is reported to show a Q3 score of 81.6% and is available at http://bioinf.cs.ucl.ac.uk/psipred/.

CFSSP (Chou and Fasman Secondary Structure Prediction Server) is an online protein secondary structure prediction server. This server predicts regions of the secondary structure of the protein sequence such as alpha-helix, beta-sheet, and turns from the amino acid sequence in a linear sequential graphical view. CFSSP implements the Chou-Fasman algorithm, which is based on an analysis of the relative frequencies of each amino acid in alpha helices, beta sheets, and turns based on the known protein structures solved by X-ray crystallography.

#### Tertiary structure assessment of the vaccine construct

For homology modelling, the final multi-epitope vaccine construct was subjected to the Iterative Threading ASSEmbly Refinement (I-TASSER)^88^ server (https://zhanglab.ccmb.med.umich.edu/I-TASSER/). It is used for generating automated protein structures and performing predictions. It is reported to design a 3D atomic model by utilizing the multiple threading alignments and iterative structural assembly simulations of the submitted amino acid sequence.

#### Refinement of the tertiary structure

Using the I-TASSER server, a three-dimensional model of the chimeric protein was obtained. Next, we refined the 3D model using two-step refinement process consisting of 3Drefine^89^ (http://sysbio.rnet.missouri.edu/3Drefine/) and GalaxyRefine^90^ (http://galaxy.seoklab.org/cgi-bin/submit.cgi?type=REFINE) online protein structure refinement servers. The 3Drefine refinement protocol utilizes iterative optimization of hydrogen bonding network combined with atomic-level energy minimization on the optimized model using a composite physics and knowledge-based force field for efficient protein structure refinement. Whereas GalaxyRefine rebuilds side chains and performs side-chain repacking and subsequent overall structure relaxation by molecular dynamics simulation.

#### Validation of the model stability

Validation is essential for the evaluation of stability and to find inherent errors that might be present in the predicted 3D protein models. For validation of the 3D model, the ProSA-web server (https://prosa.services.came.sbg.ac.at/prosa.php) was used to calculate the overall quality score in context with all the known protein structures. For generating the Ramachandran plot, MolProbity and RAMPAGE servers were used. MolProbity (http://molprobity.biochem.duke.edu/) is an all-atom structure validation online server that offers Ramachandran analysis. Ramachandran plots are used to visualize the energetically allowed and disallowed dihedral angles, psi [ψ], and phi [ϕ], of amino acids. RAMPAGE (http://mordred.bioc.cam.ac.uk/~rapper/rampage.php) is another freely accessible server that integrates the PROCHECK^91^ principle for validation of the protein model by applying a Ramachandran plot and segregating the Glycine and Proline residues plot.

#### Prediction of discontinuous B-cell epitopes for the vaccine construct

Antibodies must interact with antigen epitopes to remove the infectious agent. Therefore, the prediction of conformational epitopes such as discontinuous B-cell epitopes is important. It has been found that discontinuous B-cell epitopes comprise residues remotely located in the primary structure that are brought into proximity due to the folding of the protein and 90% of B-cell epitopes are discontinuous^92^. There are several tools for discontinuous B-cell epitopes prediction such as BEPro^93^, Ellipro^94^, and Epitopia^95^. Ellipro is based on the notion that residues that protrude from the protein surface are more accessible for antibody binding and that these protruding residues can be identified by treating the protein as an ellipsoid. Therefore, we employed ElliPro (http://tools.iedb.org/ellipro/) for discontinuous B cell epitope predictions.

#### Molecular docking of the vaccine construct with TLR-4 and several HLA alleles

Molecular docking is an important tool for studying interactions amongst biological molecules. We employed molecular docking tools to find out the effect of vaccine construct with TLR-4 and HLA alleles. Since the majority of adjuvants originate from microbial components known as PAMPs [pathogen-associated molecular patterns], the immune system responds to these PAMPs by using Toll-like receptors [TLRs]^96^.

For docking assessment, the 3D structures of different MHC molecules and human TLR-4 [PDB ID: 4G8A] were retrieved from RCSB PDB. It is observed that specific varieties of Human Leukocyte Antigen [HLA] alleles are predominant in the South and Central American region. Therefore, we focused on these specific classes of HLA in interaction studies. Molecular interactions between various HLA molecules namely HLA-A, HLA-B7 [3VCL], HLA-DRB1*01:01 [2fse], HLA-DRB1*03:01 [1a6a], HLA-DRB5*01:01 [1h15] and the designed vaccine construct was performed. Various online tools for protein–protein docking were employed to calculate the binding affinity of designing a vaccine construct with different HLA alleles and TLR-4 immune receptors. The tools include ClusPro 2.0^97^, HDOCK^98^, and PatchDock^99^. PatchDock generated numerous possible solutions that were further subjected to the refinement of the complexes using FireDock^100^.

#### Codon optimization of the chimeric protein

Java Codon Adaptation Tool or JCat server^101^ [http://www.jcat.de] was employed for codon optimization of the predicted vaccine construct. It involves the reverse transcription of the chimeric protein sequence to the nearest obtainable DNA sequence, which should contain specific genes responsible for encoding the target vaccine construct. This reverse-transcribed DNA sequence [RT-DNA] obtained is incorporated into the multiple cloning site of the pET-28a [+] vector using the SnapGene tool^102^ following our previous strategy. This was done to adapt the DNA sequence in the model organism [*E. coli* strain K12] so that this RT-DNA undergoes cellular adaptations within the model organism and the codons of RT-DNA are utilized by the model organism to produce the desired vaccine construct. This is a crucial step in vaccine construction, due to the effect of the degeneracy of codons, which can vary from one organism to another, including the cellular mechanisms that exist. To circumvent issues of glycosylation in the bacterial system, we also performed codon optimization using the yeast model (Supplementary File Y_F in Supplementary File 5).

#### Characterization of the immune profile of the vaccine construct

The simulation of the actual response of an immune system to our final vaccine construct was obtained using the C-ImmSim immune simulator [http://150.146.2.1/C-IMMSIM/index.php]. The tool was run with default parameters with three-time steps [1, 42, and 84] and without Lipopolysaccharide [LPS]. It works on Position-Specific Scoring Framework [PSSM] to simulate and predict immune interactions along with immunogenic epitopes.

#### Evaluation of genetic diversity

In order to develop a broad-spectrum *T. cruzi* vaccine, the prioritized proteins were scrutinized for their genetic diversity among fully annotated proteomes of 13 *T. cruzi* strains and different species (Supplementary Table 10). Protein sequences from these strains which are positive for that particular protein, were downloaded from NCBI RefSeq^103^ and aligned to predict conserved regions using CLC Main Workbench 21.0.2 (QIAGEN). Evolutionary distances (*p*-distances) among variant sites were also calculated for prioritized proteins using Mega 6.0^104^. The predicted epitopes were also checked for their sequence divergence among different strains and species of *Trypanosoma*. Each predicted epitope was further checked for antigenicity using VaxiJen (threshold value = 0.4)^54^. In addition, we also mapped epitopes to genomic sequences. For this purpose, we first reverse translated the epitope sequences and thereafter used pairwise alignment tools for mapping. We also checked the conservancy of epitopes through IEDB conservancy analysis tool^105^.

## Results

### Defining a potential vaccine candidate (PVC)

A Potential Vaccine Candidate (PVC) could be defined as the protein or corresponding DNA/RNA sequence that possesses properties of an “ideal vaccine” such as non-homology with the host (i.e., human) proteins to avoid the generation of a potential autoimmune response^106^, the lack of transmembrane regions to facilitate expression, antigenicity, adhesion-like properties, immunogenicity, a molecular weight of < 110 kDa, non-homology with the gut flora proteome, surface-exposure/secretion, and the presence of anchoring and/or secretion signals. Based on sequence similarity, proteins relevant to microbial pathogenesis would also be highly ranked. For our model, we label these desirable properties P_i_ [i = 1, 2, 3….n] (Supplementary Table 9).

### Selection, ranking, and filtering of PVCs

To understand the distribution of properties in the *T. cruzi* CL-Brenner (TC-CLB) proteome, we used python-based scripts to characterize the whole proteome using various bioinformatics tools. During the analysis, we found that 91.46% of all proteins [i.e., 19,602] have a molecular weight < 110 kDa, 13.20% of proteins are secretory and 7.12% are extracellular. Also, 84.80% of the proteome is dissimilar to human proteins. Likewise, we observed similar trends in proteomes of four related species and thirteen different strains of *Trypanosoma* (Table 2). In addition, we computed distributions of properties in other pathogens for comparative purposes (Supplementary Tables 11a–11c).

### Identification of subcellular location of the proteins

Using the PSORTb tool (Strategy 1A), we screened 19,602 proteins of the reference proteome of TC-CLB [Accession ID: NZ_AAHK00000000] and found that 1846 proteins were predicted to be localized in the periplasm, extracellular matrix, and outer membrane of the cell. Next, we used the PSORTb score (threshold set to 9.5) as an additional filter to shortlist 653 proteins. Alternatively, WoLF-PSORT (Strategy 1B) predicted 7274 proteins, localized in the plasma membrane and extracellular matrix. Despite using two different approaches (1A and 1B), we observed that most of the proteins (i.e., mucin TcMUCII, Mucin Associated Surface Protein (MASP), trans-sialidase, hypothetical protein, dispersed gene family (DGF-1) and subtilisin-like peptidase) were present in the top-ranking filtered list of both the approaches.

### Identification of TC-CLB proteins that are non-homologous to human proteins

To prevent undesired cross-reactivity of vaccines with the human host, the proposed vaccine candidate must be different from human proteins. Therefore, we used BLASTp to identify the 572 such non-homologous proteins out of the 653 proteins identified through PSORTb.

### Instability analysis

Protein stability is of crucial importance for the efficient presentation of antigenic peptides on MHC, which plays a decisive role in triggering strong immune reactions. Using ProtParam, the protein instability index was determined and proteins having an Instability Index (II) less than 40 were selected. This led to shortlisting of 138 proteins (out of 572) that were predicted to be stable.

### Non-allergenicity analysis

To find out non-allergenic proteins in our list, we performed a BLASTp search against the Allergen Online database and found 137 proteins to be non-allergenic.

### Evaluation of antigenicity

To determine the antigenicity of the shortlisted proteins for vaccine construction, VaxiJen 2.0 was employed. Proteins having antigenicity greater than 0.5 were selected for subsequent analysis. We identified 122 antigenic proteins out of 137 proteins using this tool.

### Adhesion prediction

Next, we performed adhesion prediction using FungalRV with a threshold value of greater or equal to − 1.2. Several studies have shown that adhesins are vital in initiating pathogen-based infections^107^. Therefore, it seemed practical to target these proteins for vaccine development. A total of 100 proteins (out of 122) were predicted to possess desired properties similar to adhesin proteins. We used these top 100 proteins for subsequent analysis as a filtered list. It was also found that several hits belonging to the same gene/protein family such as trans-sialidases, and mucin-associated surface protein were present in the top 100 list. In VAX-Elan, we have also included an option to filter (or include) multi-copy genes/proteins for subsequent analysis^108^.

### Shortlisted potential vaccine candidates (PVCs)

The top 100 shortlisted proteins were analysed further to evaluate the presence of additional criteria (TM α-helices, signal peptides, essentiality, and virulence) to narrow down the best eight proteins as PVCs. These include Dispersed gene family [XP_813527.1], subtilisin-like serine peptidase [XP_809835.1], DNAJ Chaperone protein [XP_806816.1], Mucin-associated Surface Protein [MASP] [XP_809166.1], Mucin TcMUCII [XP_816522.1], Trans-sialidase [XP_818708.1], 90 kDa surface protein [XP_815016.1] and a hypothetical protein [XP_821916.1], each belonging to different protein families (Table 3). We also used alternative strategies (see “Methods”) which also reported these PVCs in their top-ranking lists. Next, we independently checked these proteins as PVCs from scientific literature using text mining and manual curation approaches (Supplementary Table 12).

**Table 3:** Ranking of unique proteins with the highest antigenic score. Here the hypothetical protein has displayed similarity with regulator sigma E protease during the Blast search.

Top proteins (unique) after filtration	VaxiJen score
XP_813527.1 (DGF-1)	0.61
XP_809835.1 (substilin-like serine peptidase)	0.65
XP_806816.1 (DNAJ Chaperone protein)	0.51
XP_809166.1 (MASP)	1.41
XP_816522.1 (Mucin TcMUCII)	1.16
XP_818708.1(Trans-sialidase)	0.80
XP_815016.1 (Surface Protein)	0.85
XP_821916.1 (hypothetical protein)	0.76

### Epitope predictions

#### Linear B-cell epitopes identification

We identified a total of 1173 linear B cell epitopes in 8 PVCs using different prediction servers (ABCPred, BCEPRED & Bepipred). The maximum number of epitopes [510 epitopes] were found in Dispersed Gene Family protein [XP_813527.1] whereas the minimum number of epitopes [34 epitopes] were identified for Hypothetical Protein [XP_821916.1]. We ranked the epitopes based upon antigenicity value generated by the VaxiJen 2.0 tool (threshold: 0.5; target organism used as ‘Parasite’). Further, we found that approximately 295 epitopes were predicted by multiple servers. In Table 4, we show the highest-ranked epitope found in each protein, shortlisted for further analysis.

**Table 4:** Predicted linear B-cell epitopes in the selected proteins for designing vaccine constructs.

S. no.	Protein ID	Top BCL epitopes	VaxiJen score
1	XP_813527.1	GSCGCRC	3.51
2	XP_809835.1	PLLLFVFF	3.06
3	XP_806816.1	VHINLKQ	1.49
4	XP_809166.1	TSPLFPLLLVVAC	1.23
5	XP_816522.1	MTCRLLCALLVLALCCCPSVCVT	0.77
6	XP_818708.1	SLWSVRL	1.61
7	XP_815016.1	DVPPSSLP	0.89
8	XP_821916.1	EKPQCLLLSSGILVDVLMR	1.15

#### T-cell epitopes [CTL] prediction

First, we identified 16,385 CTL epitopes in the eight shortlisted proteins. Second, we found 221 epitopes (out of 16,385) that were predicted by four different prediction tools namely NETMHC, EpiJen, Propred1, and NetCTL. Third, we selected eight high-scoring epitopes for subsequent work (Table 5).

**Table 5:** Predicted linear cytotoxic T-lymphocyte epitopes in the selected proteins for designing vaccine constructs.

S. no.	Protein ID	Top CTL epitopes	VaxiJen score
1	XP_813527.1 (DGF-1)	DAALLGGDY	2.09
2	XP_809835.1 (subtilisin-like serine peptidase)	GVDFDSCFF	1.84
3	XP_806816.1 (DNAJ Chaperone protein)	KTGRNGDMY	1.81
4	XP_809166.1 (MASP)	STDDHATGS	1.75
5	XP_816522.1 (Mucin TcMUCII)	GTDGVTGTT	1.48
6	XP_818708.1 (Trans-sialidase)	SSDADPTVV	1.03
7	XP_815016.1 (Surface Protein)	LLVLAALTY	0.94
8	XP_821916.1 (hypothetical protein)	YTCGTSCAV	0.75

#### Helper T lymphocytes [HTL] prediction

With the IEDB MHC-II prediction tool, HTL cell epitopes were predicted with the highest binding corresponding to the alleles from the human 7-allele reference set i.e., HLA-DRB alleles. Based on the percentile rank as well as IC50 value [< 50 nM], 41 epitopes were selected for further analysis. Out of those, a total of 8 HTL epitopes were chosen for the vaccine construct (Table 6).

**Table 6:** Predicted helper T-lymphocyte epitopes in the selected proteins for designing vaccine constructs.

S. no.	Protein ID	Top HTL epitopes	VaxiJen score
1	XP_813527.1 (DGF-1)	GSFVMDGTVALGGAG	1.75
2	XP_809835.1 (subtilisin-like serine peptidase)	KAPRGRIIRLQYLRF	1.68
3	XP_806816.1 (DNAJ Chaperone protein)	TGVSKNGRQLRVSGK	1.79
4	XP_809166.1 (MASP)	ASGVLGENGSHMPDG	1.45
5	XP_816522.1 (Mucin TcMUCII)	STSGSAEPTKKVQEQ	1.23
6	XP_818708.1 (Trans-sialidase)	MLVGKYSRNAAAGAR	1.1
7	XP_815016.1 (Surface Protein)	LKSWWQRNVETKAVT	1.32
8	XP_821916.1 (hypothetical protein)	SGILVDVLMRTSAHR	1.01

#### The assemblage of multi-epitope subunit vaccine construct

**Figure 6:**
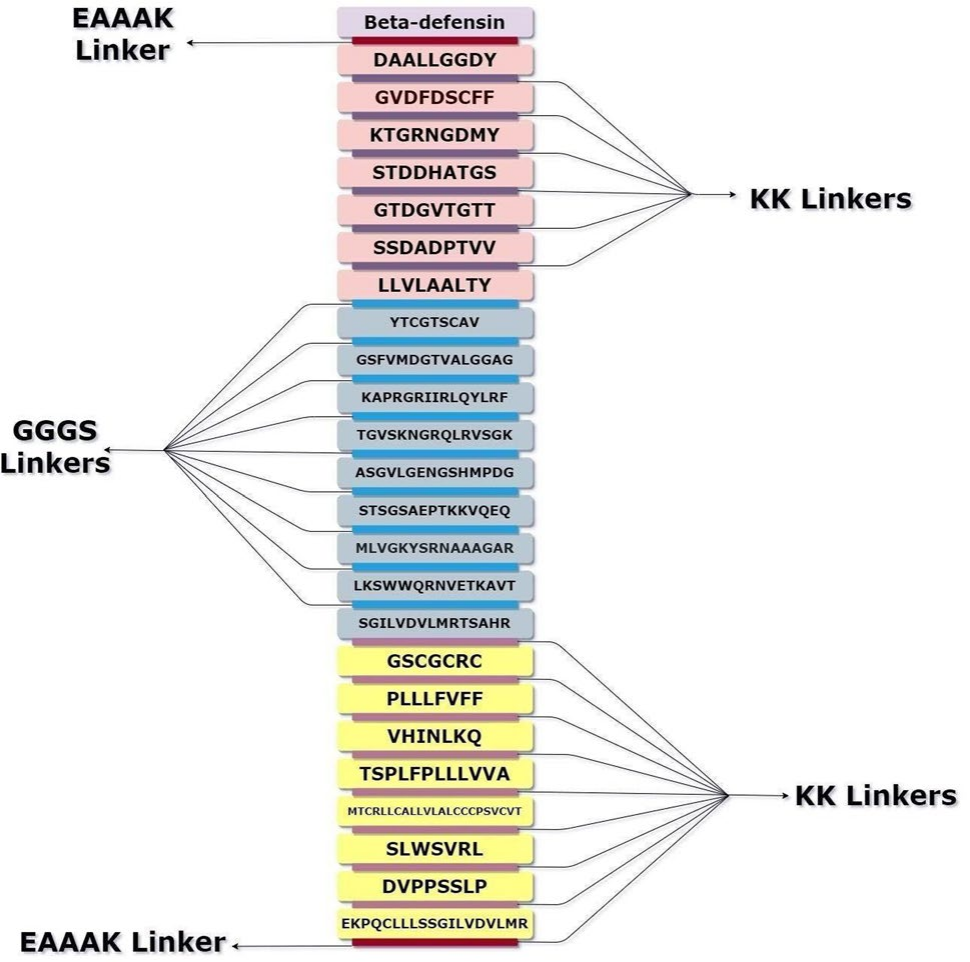
Multi-epitope vaccine constructs for Chagas disease. The vaccine construct consists of 24 epitope sequences, belonging to CTL, HTL and BCL epitopes of 8 *T. cruzi* proteins. Beta-defensin (Light purple) was used as the adjuvant and is linked to the top CTL epitope (light pink) using an EAAAK (maroon) linker. The other CTL epitopes were linked to each other using GGGS (violet) linkers. The last CTL epitope and the first HTL epitope (blue), as well as the other HTL epitopes were connected through an AAY (sky blue) linker. The last HTL epitope and the first BCL epitope (yellow) as well as the other BCL epitopes were connected through a KK (purple) linker. An EAAAK (maroon) linker was added at the end of the sequence for increasing stability (Draw.io—https://www.diagrams.net/-14.6.10).

The vaccine (V1) was constructed from high-scoring CTLs, B-cell epitopes, and HTL epitopes. To enhance its immunogenicity, a Beta-defensin adjuvant [Accession ID: AGV15514.1] was obtained from NCBI and incorporated into V1 (Fig. 6).

#### Evaluation of antigenicity and allergenicity of the vaccine constructs

The predicted vaccine constructs were labelled as non-allergenic as predicted by AlgPred and AllerTop tools. The antigenicity value of the vaccine constructs was observed highest for V1 (1.06) as evaluated by Vaxijen 2.0 (Table 7).

**Table 7:** The top three vaccine constructs V1, V2, and V3 made using Beta-defensin, L7/L12 Ribosomal protein, and Gaba protein adjuvants along with the top BCL, HTL, and CTL epitope sequences.

Vaccine	Sequence	Antigenic propensity
V1	GIINTLQKYYCRVRGGRCAVLSCLPKEEQIGKCSTRGRKCCRRKKEAAAKDAALLGGDYGGGSGVDFDSCFFGGGSKTGRNGDMYGGGSSTDDHATGSGGGSGTDGVTGTTGGGSSSDADPTVVGGGSLLVLAALTYGGGSYTCGTSCAVAAYGSFVMDGTVALGGAGAAYKAPRGRIIRLQYLRFAAYTGVSKNGRQLRVSGKAAYASGVLGENGSHMPDGAAYSTSGSAEPTKKVQEQAAYMLVGKYSRNAAAGARAAYLKSWWQRNVETKAVTAAYSGILVDVLMRTSAHRKKGSCGCRCKKPLLLFVFFKKVHINLKQKKTSPLFPLLLVVAKKMTCRLLCALLVLALCCCPSVCVTKKSLWSVRLKKDVPPSSLPKK EKPQCLLLSSGILVDVLMREAAAK	1.06
V2	MSDINKLAETLVNLKIVEVNDLAKILKEKYGLDPSANLAIPSLPKAEILDKSKEKTSFDLILKGAGSAKLTVVKRIKDLIGLGLKESKDLVDNVPKHLKKGLSKEEAESLKKQLEEVGAEVELKEAAAKDAALLGGDYGGGSGVDFDSCFFGGGSKTGRNGDMYGGGSSTDDHATGSGGGSGTDGVTGTTGGGSSSDADPTVVGGGSLLVLAALTYGGGSYTCGTSCAVAAYGSFVMDGTVALGGAGAAYKAPRGRIIRLQYLRFAAYTGVSKNGRQLRVSGKAAYASGVLGENGSHMPDGAAYSTSGSAEPTKKVQEQAAYMLVGKYSRNAAAGARAAYLKSWWQRNVETKAVTAAYSGILVDVLMRTSAHRKKGSCGCRCKKPLLLFVFFKKVHINLKQKKTSPLFPLLLVVAKKMTCRLLCALLVLALCCCPSVCVTKKSLWSVRLKKDVPPSSLPKKEKPQCLLLSSGILVDVLMREAAAK	0.83
V3	MAENPNIDDLPAPLLAALGAADLALATVNDLIANLRERAEETRAETRTRVEERRARLTKFQEDLPEQFIELRDKFTTEELRKAAEGYLEAATNRYNELVERGEAALQRLRSQTAFEDASARAEGYVDQAVELTQEALGTVASQTRAVGERAAKLVGIELEAAAKDAALLGGDYGGGSGVDFDSCFFGGGSKTGRNGDMYGGGSSTDDHATGSGGGSGTDGVTGTTGGGSSSDADPTVVGGGSLLVLAALTYGGGSYTCGTSCAVAAYGSFVMDGTVALGGAGAAYKAPRGRIIRLQYLRFAAYTGVSKNGRQLRVSGKAAYASGVLGENGSHMPDGAAYSTSGSAEPTKKVQEQAAYMLVGKYSRNAAAGARAAYLKSWWQRNVETKAVTAAYSGILVDVLMRTSAHRKKGSCGCRCKKPLLLFVFFKKVHINLKQKKTSPLFPLLLVVAKKMTCRLLCALLVLALCCCPSVCVTKKSLWSVRLKKDVPPSSLPKKEKPQCLLLSSGILVDVLMREAAAK	0.99

#### Analysis of solubility and physicochemical properties

Using ProtParam, the theoretical molecular weight of the vaccine construct V1 was found to be 42.3 kDa constructed with Beta-defensin as an adjuvant (406 amino acids) whereas the theoretical isoelectric point [pI] of the protein was found to be 9.70 which suggest that the vaccine construct is highly charged. The instability index [II] was estimated to be 30.95, indicating that the vaccine construct is stable (II < 40 indicates stability). V1 was predicted to be thermostable (Aliphatic index—78.37). V1 was also found to be hydrophilic (the predicted hydropathicity or GRAVY came out to be − 0.062). The presence of negative value scores suggests hydrophilic epitopes that are likely to be present in the outer surface and have more chance to elicit the high immunogenicity in the host cell. Furthermore, the solubility value of the vaccine construct is 0.651 as predicted by the Protein-Sol tool which has a threshold value of 0.45 indicating that the vaccine construct has a higher solubility than the average soluble *E. coli* protein from the experimental dataset utilized by this tool (Table 8). The half-life was estimated to be 30 h in mammalian reticulocytes in vitro, and > 20 h in yeast in vivo*,* and > 10 h in *E. coli *in vivo*.*

**Table 8:** Comparison of physicochemical and solubility properties of different vaccine constructs.

Physicochemical properties	Vaccine 1	Vaccine 2	Vaccine 3
Antigenic propensity	1.062	0.83	0.992
Solubility	0.651	0.601	0.472
Molecular weight	42.3 kDa	50.70 kDa	54.7 kDa
Allergenicity	Non-allergenic	Non-allergenic	Non allergenic
Hydropathicity	− 0.062	− 0.056	− 0.14
Amino acids	406	485	520
Theoretical isoelectric point (pI)	9.7	9.45	9.05
Aliphatic index	78.37	89.94	83.23
Instability index (II)	30.95	27.71	32.94

#### Secondary structure analysis

**Figure 7:**
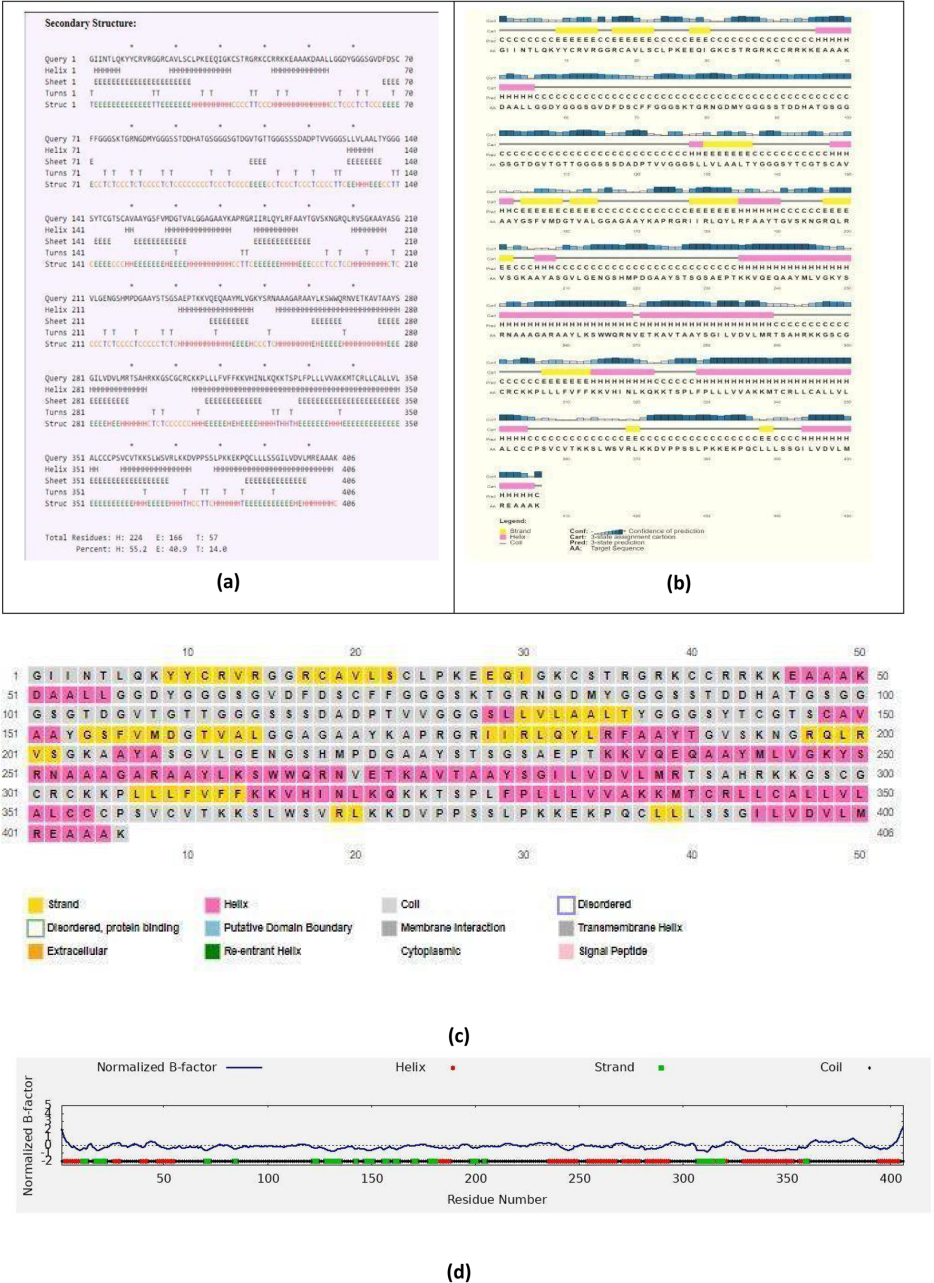
Secondary structure prediction of the final vaccine sequence using (**a**) CFSSP, (**b**) and (**c**) PSIPRED. (**d**) Graph of normalized B-factor predicted by I-TASSER.

Using the CFSSP tool and PSIPRED, we found that V1 consists of 55.2% helix, 14.0% turns and 40.9% of sheets (Fig. 7a–d). The presence of random coils in the vaccine construct suggests the existence of natively unfolded protein regions that can be identified by antibodies that are produced in response to infection^109^.

#### Tertiary structure assessment of the vaccine construct

**Figure 8:**
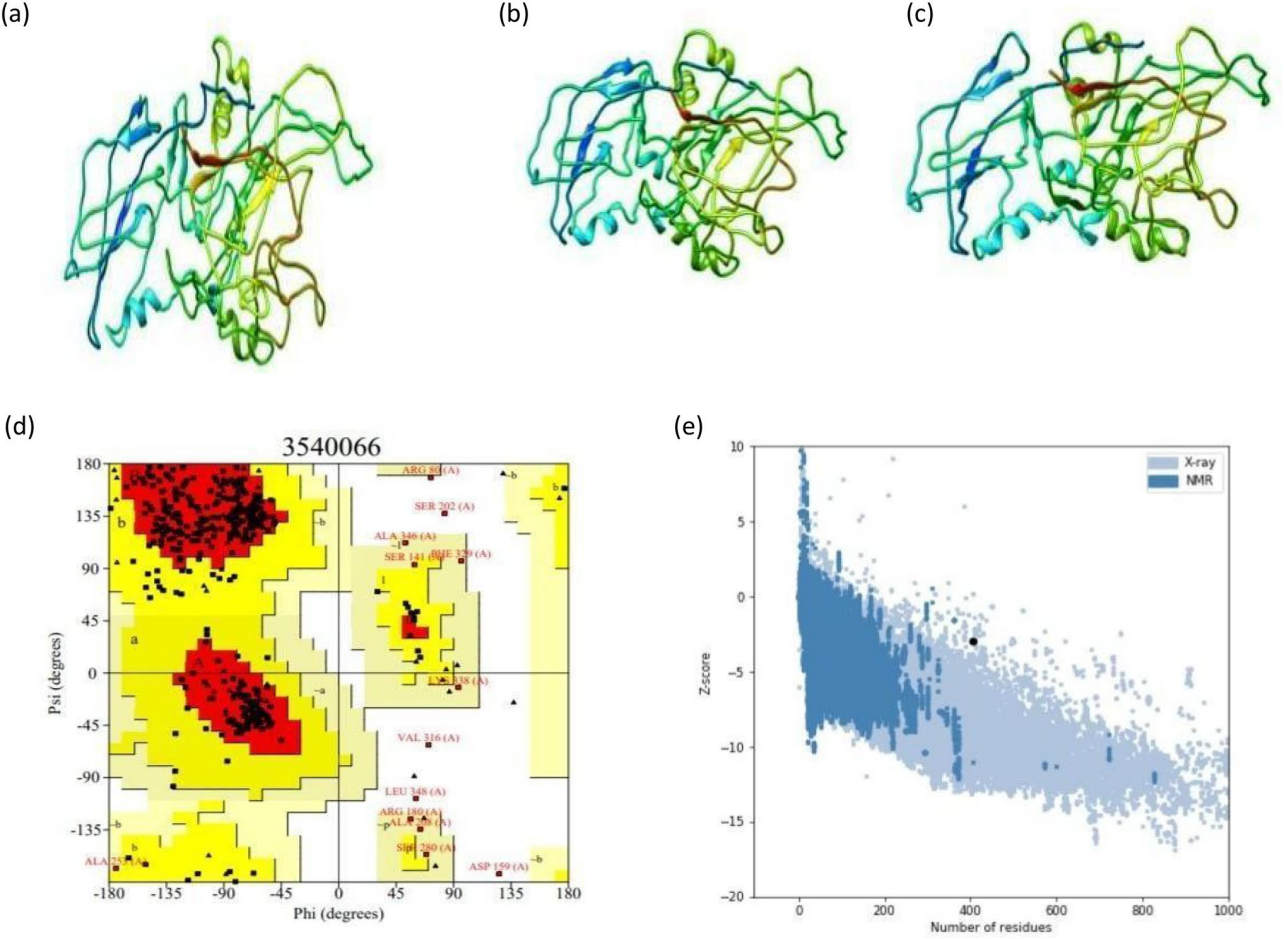
Modeling, refinement and validation of tertiary structures. (**a**) Multi-epitope vaccine chimeric protein 3D model generated using homology modelling (Chimera 1.15—https://www.cgl.ucsf.edu/chimera/download.html). (**b**) Refined model using 3Drefine (Chimera 1.15—https://www.cgl.ucsf.edu/chimera/download.html). (**c**) GalaxyRefine generated refined 3D structure (Chimera 1.15—https://www.cgl.ucsf.edu/chimera/download.html). (**d**) Ramachandran plot of vaccine construct V1. (**e**) Prosa-Web giving a Z- score of -2.9.

The tertiary structure models of the chimeric construct were predicted by the I-TASSER server by employing several threading templates [1kj6, 5nf2A, 1kj6A, 5ke1, 4om9A, 5ke1A, 4kh3A]. Out of 5 predicted results, model 1 was found to be the best one based upon the scores. In this study, the highest C-score model, derived from the homology modelling was selected for subsequent refinement protocol (Fig. 8a). The TM-score is defined to assess the topological similarity of the two protein structures. The TM-Score for our vaccine construct was found to be 0.56 ± 0.15 and the RMSD value was 9.6 ± 4.6 Å. It has been reported that a model with a TM score greater than 0.5, shows accurate topology, whereas a model with a TM score less than 0.17 indicates nonspecific similarity.

#### Refinement of the tertiary structure

The putative chimeric vaccine model was refined by the 3Drefine server (http://sysbio.rnet.missouri.edu/3Drefine/) and subsequently by GalaxyRefine (http://galaxy.seoklab.org/cgi-bin/submit.cgi?type=REFINE). The 3D-refine server-generated five models, out of which top-ranking model having favourable parameters such as lowest 3Drefine score (29,567.2), GDT-HA (0.96), RMSD (0.37 Å), lowest RWPlus score (− 63,611.70), and MolProbity (3.55). We also shortlisted Model 1 (from GalaxyRefine server) using a clash score (20.8), a score of poor rotamers (0.3), and the Ramachandran plot with a statistical score (89.4%) for downstream validation studies (Fig. 8b,c).

#### Validation of model stability

Ramachandran plot analysis of the protein model by ProCHECK-web predicted that 82.4% of amino acids were present in favoured regions. Moreover, 13.7% of the residues were present in the allowed regions, and only 1.5% of proteins were present in the disallowed or outlier boundary (Fig. 8d) indicating the quality of the model. The ProSA-web server authenticated the overall quality and errors that may potentially arise in the refined model. The refined model (obtained in this study) was considered to be appropriate with a Z-score of − 2.9 (Fig. 8e).

#### Prediction of discontinuous B-cell epitopes

**Figure 9:**
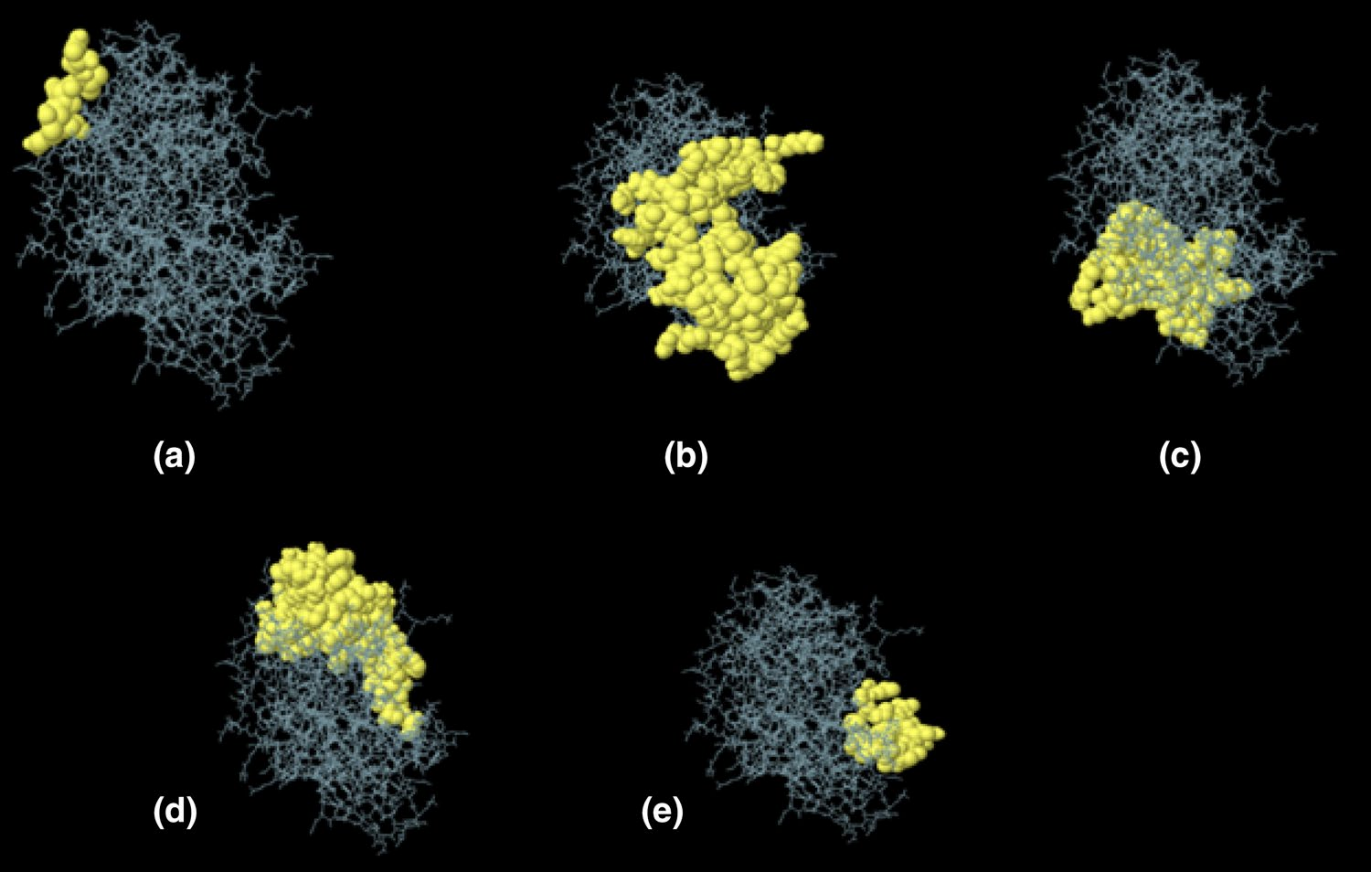
Discontinuous B-cell epitopes predicted by ElliPro. (**A**–**E**): 3D representation of conformational or discontinuous epitopes of the most antigenic chimeric protein from *T. cruzi* CL Brenner. Epitopes are shown as yellow surfaces, and the bulk of the protein is represented in grey sticks (JSmol 13.3.9—https://sourceforge.net/projects/jsmol/).

Ellipro estimated the five discontinuous B-cell epitopes and revealed the presence of 221 total residues among them (with score variation from 0.61 to 0.75) (Table 9, Fig. 9).

**Table 9:** Discontinuous B-cell epitopes predicted by the ElliPro. Two hundred and twenty-one residues were found to be located in five discontinuous B-cell epitopes of the refined vaccine model.

S. no.	Residues	Number of residues	Score
1	A: N269, A: V270, A: E271, A: T272, A: K273	5	0.75
2	A:R14, A:G15, A:G16, A:R17, A:V20, A:S22, A:C23, A:L24, A:P25, A:K26, A:E27, A:E28, A:Q29, A:I30, A:G31, A:K32, A:C33, A:S34, A:T35, A:R36, A:G37, A:R38, A:K39, A:C40, A:C41, A:R42, A:R43, A:K45, A:E46, A:A47, A:A48, A:A49, A:K50, A:D51, A:A52, A:A53, A:L54, A:L55, A:G56, A:G57, A:D58, A:Y59, A:G60, A:G61, A:G62, A:S63, A:G64, A:V65, A:D66, A:F67, A:D68, A:S69, A:N81, A:G82, A:D83, A:M84, A:G86, A:G87, A:G88, A:S89, A:D92, A:D93, A:L135, A:G138, A:G139, A:G140, A:S141, A:Y142, A:T143, A:C144, A:G145, A:T146, A:S147, A:C148, A:A149, A:A151, A:A152, A:Y153	78	0.70
3	A:S226, A:T227, A:S228, A:G229, A:S230, A:A231, A:E232, A:P233, A:T234, A:K235, A:K236, A:V237, A:E239, A:Q240, A:R302, A:C354, A:P356, A:C359, A:V360, A:T361, A:K362, A:K363, A:S364, A:L365, A:W366, A:S367, A:V368, A:R369, A:L370, A:K371, A:D373, A:V374, A:P375, A:P376, A:S377, A:S378, A:L379, A:P380, A:K381, A:E383, A:K384, A:P385, A:Q386, A:C387	44	0.65
4	A:I3, A:N4, A:T5, A:L6, A:Q7, A:K8, A:Y10, A:G166, A:A167, A:G168, A:A169, A:A170, A:Y171, A:K172, A:A173, A:P174, A:R175, A:G176, A:R177, A:I178, A:I179, A:R180, A:L181, A:Q182, A:Y183, A:L184, A:R185, A:F186, A:A187, A:A188, A:Y189, A:T190, A:V192, A:S193, A:K194, A:A260, A:Y261, A:L262, A:K263, A:S264, A:W265, A:W266, A:Q267, A:R268, A:R294, A:V311, A:F312, A:F313, A:K314, A:K315, A:V316, A:H317, A:I318, A:N319, A:L320, A:K321, A:Q322, A:K324, A:T325, A:S326, A:P327, A:L328, A:F329, A:P330, A:L347, A:L348, A:L389, A:L390, A:S391, A:S392, A:G393, A:I394, A:D397, A:V398	74	0.65
5	A:V211, A:L212, A:G213, A:E214, A:N215, A:G216, A:S217, A:P220, A:R289, A:T290, A:S291, A:A292, A:H293, A:K295, A:K296, A:G297, A:S298, A:C299, A:L331, A:L332	20	0.61

#### Molecular docking of the chimeric protein with TLR-4

The CastP^110^ server was employed for determining protein binding and hydrophobic contact sites on the protein surface. One of the potential binding pockets (A) was identified for the interaction with a TLR-4 receptor. It was found that the molecular surface area of the pocket ‘A’ was 6008.1 Å^2^ with a molecular surface volume of 39,003.9 Å^3^, the mouth molecular area was about 1088.07 Å^2^, and the molecular surface sum was calculated to be 1888.9 Å. CPORT predicted G1, A19, L21, C33 as active amino acid residues in the adjuvant sequences; A52, L54, L55, G56, G57, D58, T59, G60, D68, S69, C70, F72, M84, G87, T137, G138, G140, S141, Y142, T143, C144, G145, T146, C148, P174, G176, I178, I179, R180, L181, Y183, L184, R185, F186, A187, Y189, N215, A255, G300, C301, P306, L307, L308, L309, F310, V311, F312, F313, K314, K315, V316, H317, I318, N319, L320, K321, S326, L328, F329, P330, L333, C345, L348, V349, L350, A351, L352, C353, C354, C355, P356, S357, D373, L388, L389, L390, S391, S392, G393, I394, L395, V396, V398, L399 from the chimeric protein joined with linker sequences^111^.

**Figure 10:**
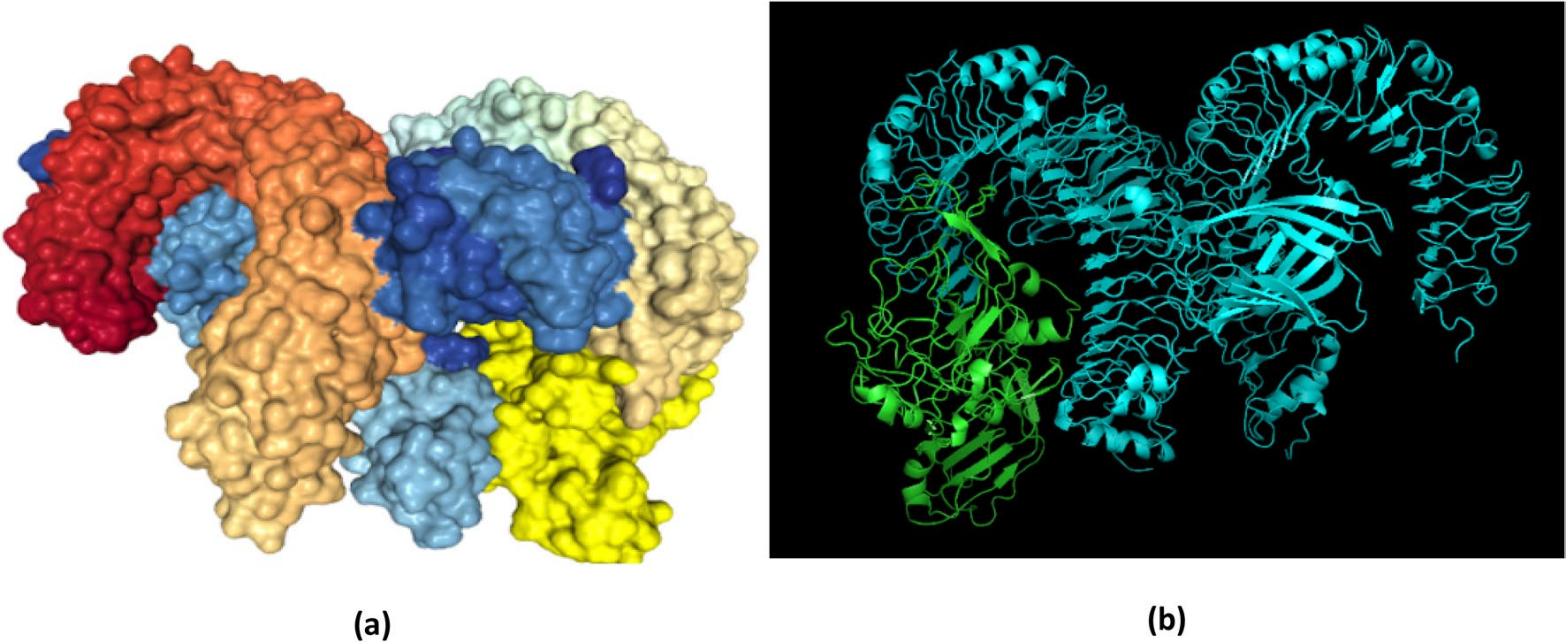
Molecular docking of subunit vaccine with the immune receptor—TLR4. (**a**) Docked image of the chimeric protein generated by HDOCK server having a binding energy score of -314.02. The rainbow-colored complex represents the TLR4 receptor molecule, while the golden-yellow colour denotes vaccine construct V1. (**b**) ClusPro generated model 5, which represents the protein–ligand complex (cyan–green). The lowest binding energy of -973.2 kcal was achieved for this model (Chimera 1.15—https://www.cgl.ucsf.edu/chimera/download.html).

For the highest-ranking docked complex, the ClusPro tool revealed the lowest total intermolecular energy (− 973.2 kcal/mol), indicating a good interaction between V1 and TLR-4. The HDOCK server predicted the binding energy for the protein–protein complex as − 314.02 kcal/mol (Fig. 10). The refinement of PatchDock docking results, as obtained by the Firedock result also showed the lowest global energy values (Table 10).

**Table 10:** Molecular docking results using the PatchDock server.

Vaccine construct	PDB ID of the HLA alleles	Solution no.	Global energy	Hydrogen bond energy	ACE	Score	Area
VACCINE 1	3vcl1	28	8.99	0.0	− 136.02	14,722	2001.1
	1a6a	82	8.82	− 1.63	− 284.02	14,114	2888.6
	1h15	48	4.17	− 2.06	− 264.30	15,388	3810.5
	2fse	9	− 29.11	− 2.32	− 192.83	16,506	2909.7
	TLR4	83	− 19.85	− 3.35	− 152.69	15,728	2531.6

The model was refined further using Firedock server.

#### Codon optimization of the chimeric protein

**Figure 11:**
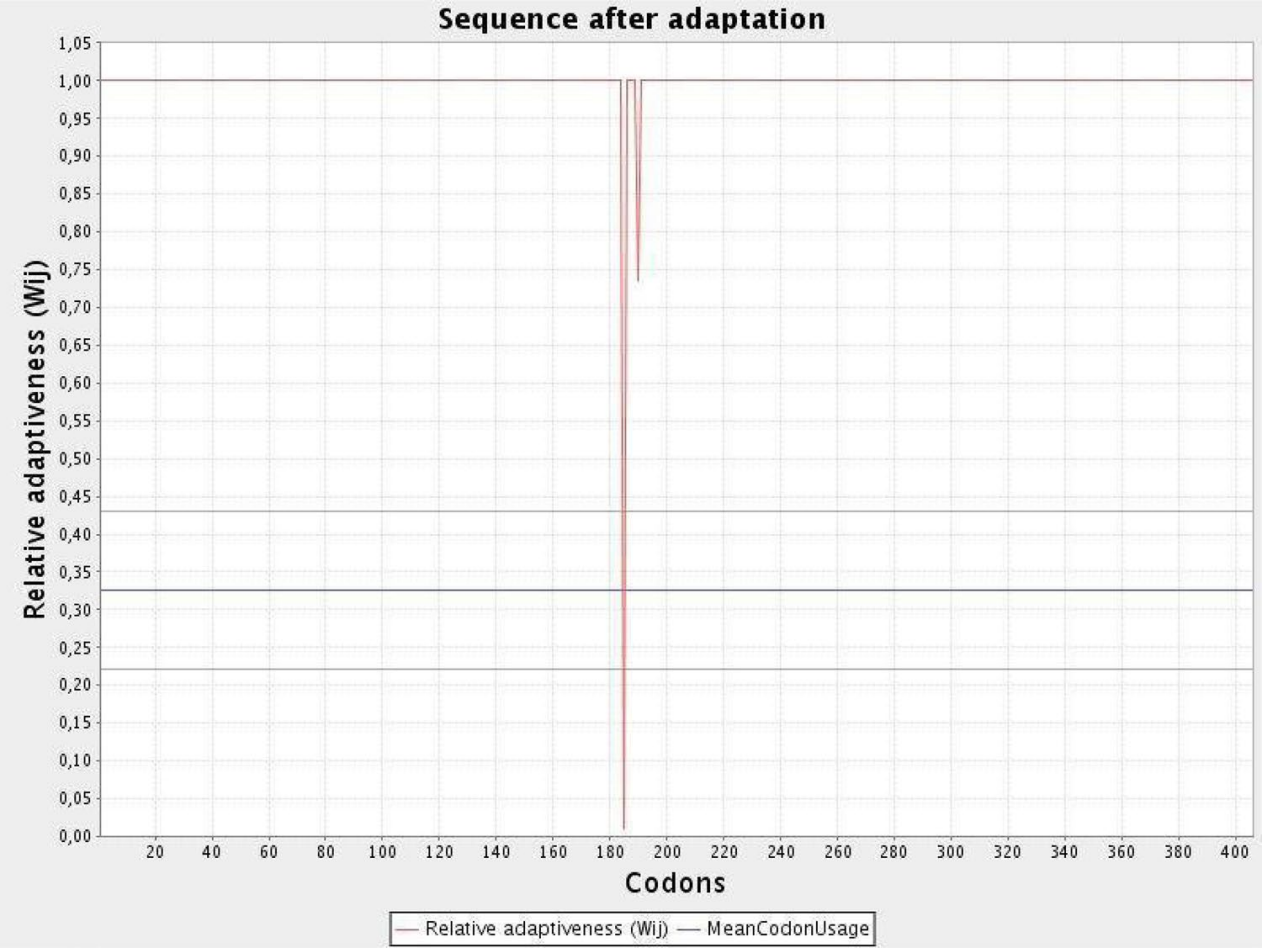
Codon optimization of the vaccine construct V1. Here, CAI of the optimized codon and average GC content were 0.98 and 51.8% respectively.

**Figure 12:**
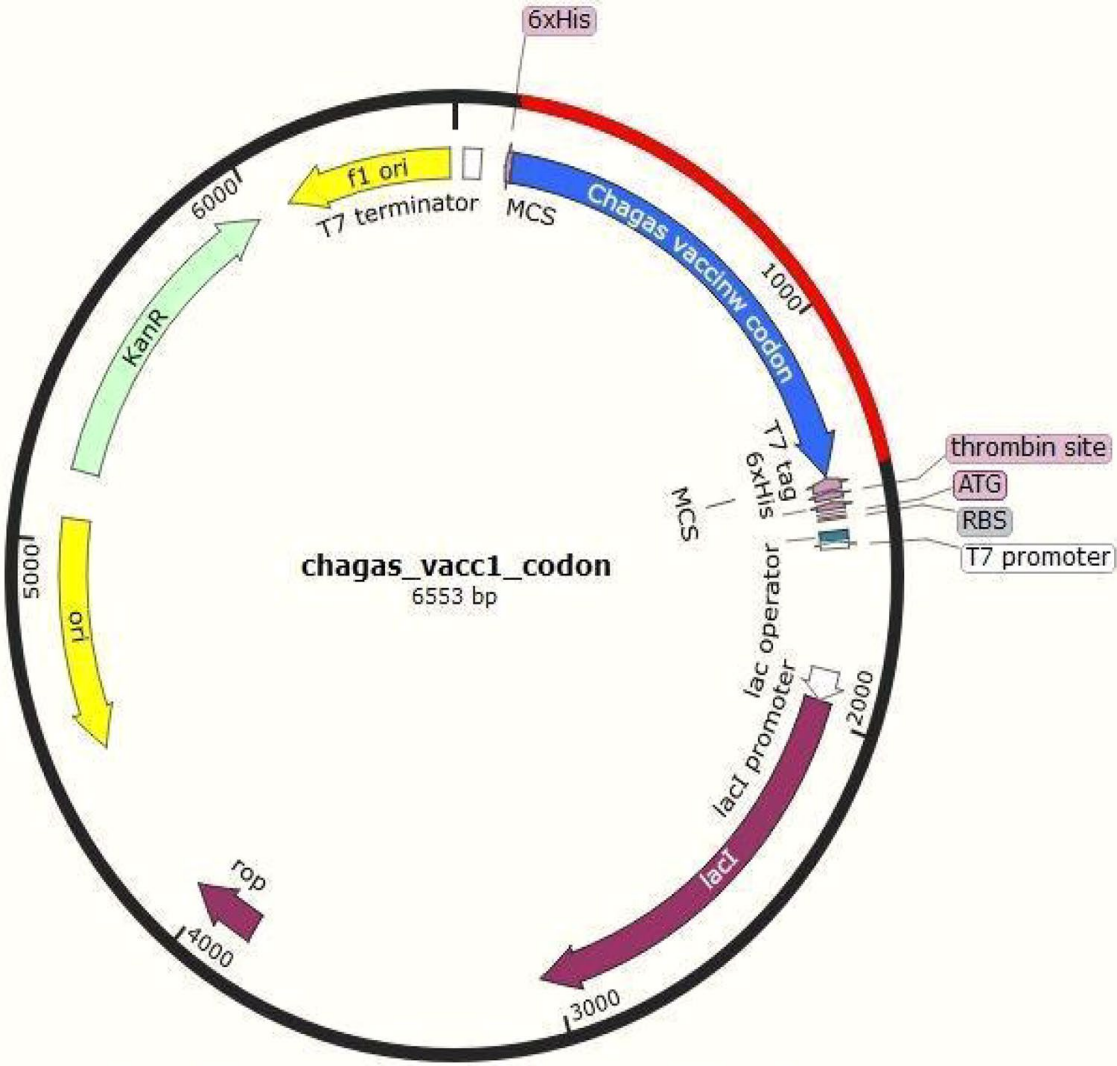
In silico cloning of optimized codons encoding vaccine protein into pET28a (+) vector to ensure expression in microbial systems. The DNA sequence was inserted into the multiple cloning-site of the cloning vector. Here, the red portion denotes the gene sequence of our designed vaccine construct while the black portion denotes the backbone of the vector. All colored arrows denote the location and direction of the expression of gene. The blue portion shows vaccine codon sequence while green denotes kanamycin resistance gene, violet represents vector genes and yellow denotes origin of replication (SnapGene 5.2.5.1—https://www.snapgene.com/).

JCAT results revealed that the optimized codon sequence has a length of 1308 nucleotides and its CAI (Codon Adaptation Index) was predicted to be 0.98, with an average of 51.88% GC for the adapted sequence. These values indicate stable expression of the designed vaccine construct in the selected microbial host. For optimal gene expression, SnapGene software was employed, the designed chimeric protein sequence was integrated into the *E. coli* pET-28a [+] vector by incorporating restriction sites which were followed by cloning into the vector using published methods (Figs. 11 and 12).

#### Characterization of the immune profile of the vaccine construct

**Figure 13:**
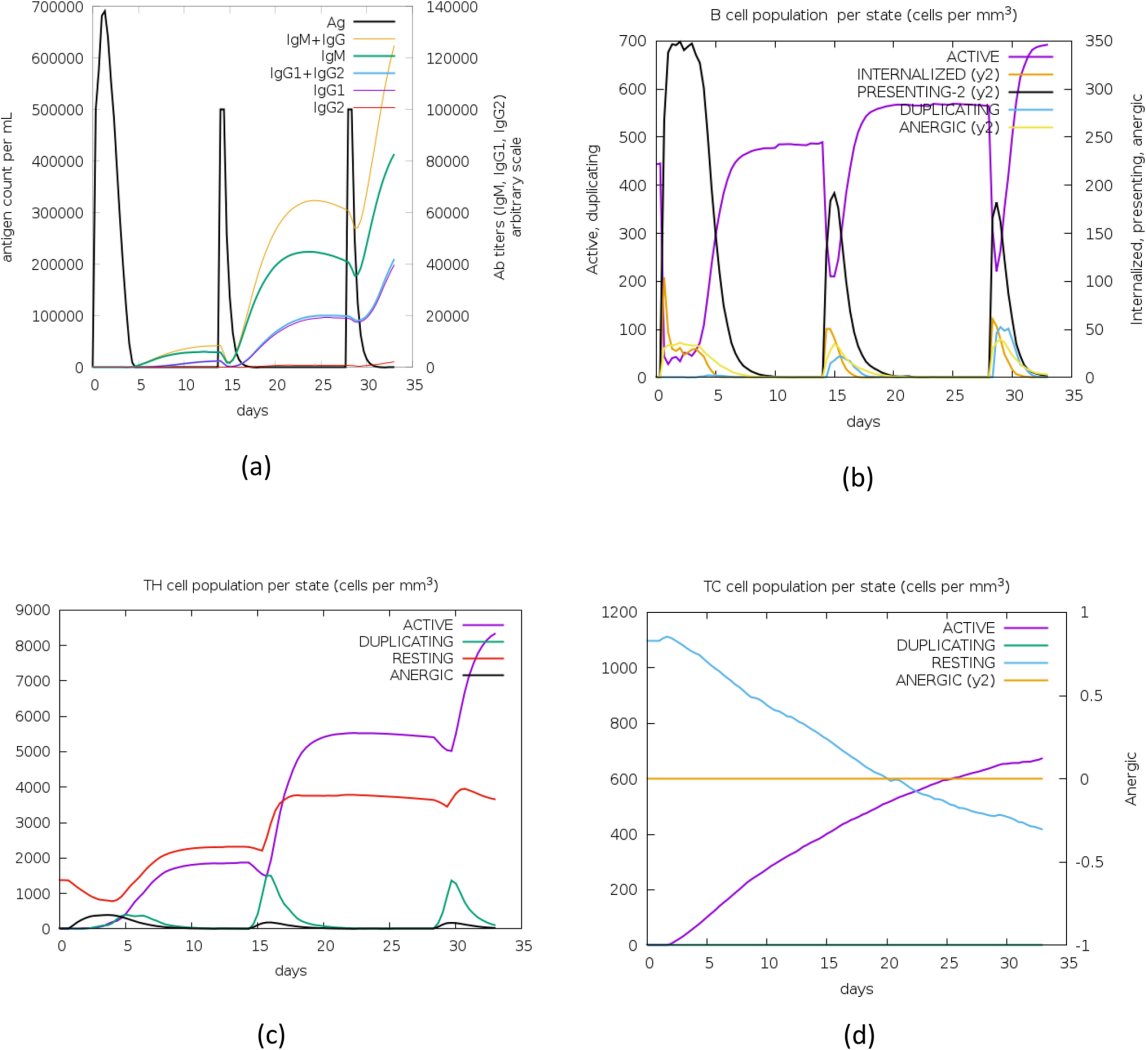
Molecular simulations of the chimeric protein. (**a**) Successive antigen injections leading to immunoglobulins production (Colored peaks indicate black vertical lines, along with other sub-classes of immune cells). (**b**) Post-insertion development in B-cell population per state as per observation after inserting three injections. (**c**) Development of T-helper cells (**d**) T-cytotoxic cell population per state after injections. The resting phase denotes those cells that are not presented with the antigens and energy state denotes cells that are tolerant to antigens due to repeated exposure, thereby indicating a lack of immune responsiveness.

With C-ImmSim, the immune response of the final vaccine construct was analysed. Results of the simulated immune responses indicated an increased surge in the induction of secondary and tertiary immune responses. At the first dose, a high surge of IgM and IgG1 antibodies was predicted. However, these titters increased exponentially with the second and third dose. Furthermore, an increase in active B-cell, CTL, and HTL cell populations was predicted for all doses (Fig. 13).

#### Evaluation of genetic diversity

Protein sequences of the prioritized proteins were extracted from 13 *T. cruzi* annotated proteomes which were aligned to predict conserved regions (Supplementary Table 13). Five proteins namely DNAJ chaperon protein, subtilisin-like serine peptidase, DGF-1, MASP, and trans-sialidase displayed strong homology (above 80%) across 13 different strains of *T. cruzi*.

In the context of the DNAJ protein, the estimated evolutionary distance (*p*-distance) was found to be 0.005 (across 13 strains) and 0.746 (across species). Whereas for TS, *p*-distance was found to be 0.234 (across strains) and 0.795 (across species). Next, we extracted all the copies of TS from TC-CLB proteome and computed evolutionary divergence (0.616) as well (Supplementary Tables 14a–14c).

**Figure 14:**
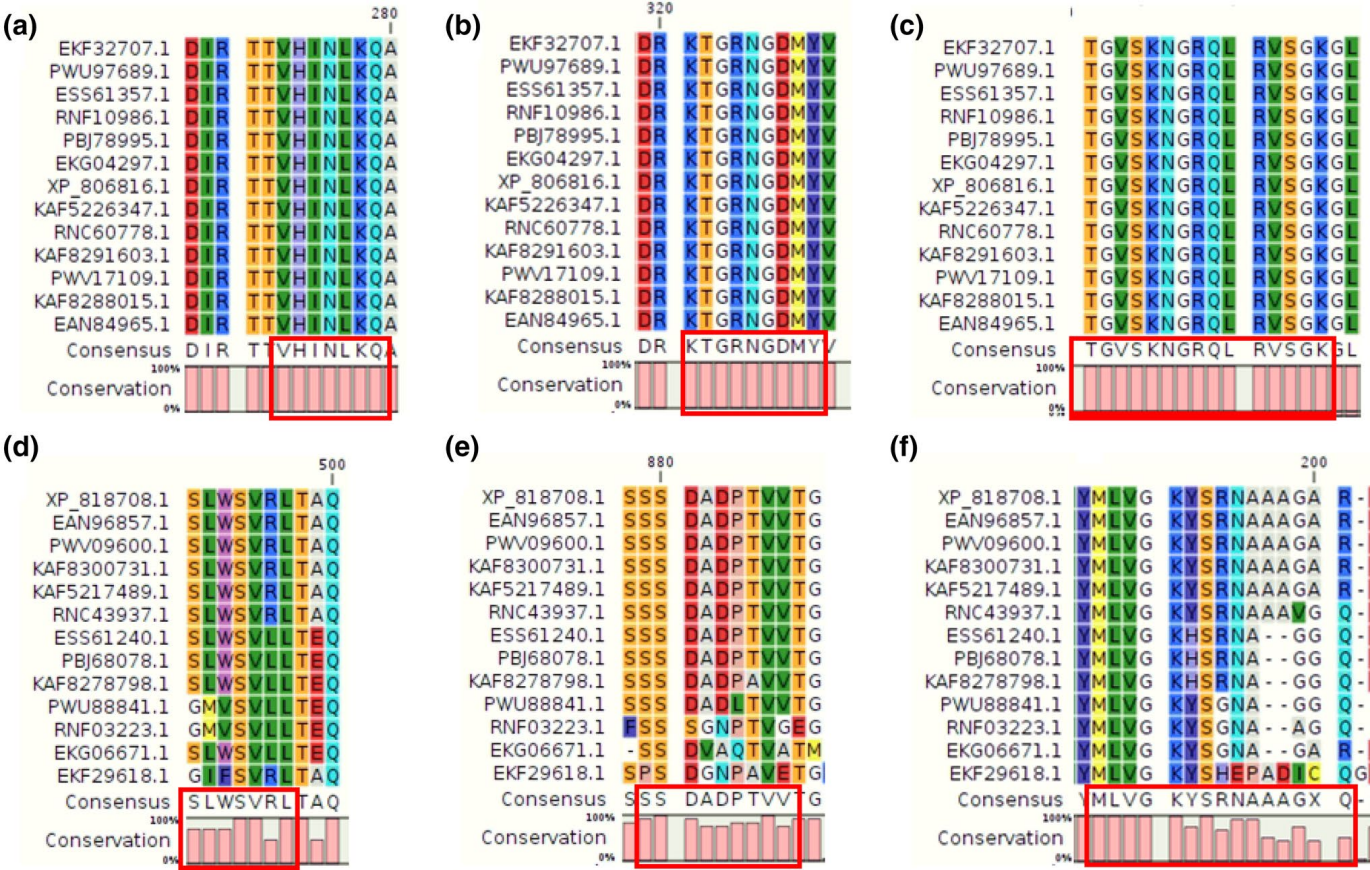
Aligned regions showing conserved epitopes among various strains of *T. cruzi*. Individual targeted proteins (DNAJ chaperon and trans-sialidase proteins) among the 12 strains are aligned using CLC Main workbench and the regions with conserved epitopes (sequences) have been shown in the red boxes. (**a**) DNAJ BCL epitope; (**b**) DNAJ CTL epitope; (**c**) DNAJ HTL epitope; (**d**) trans-sialidase BCL epitope; (**e**) trans-sialidase CTL epitope; and (**f**) trans-sialidase HTL epitope.

Estimates of evolutionary divergence between sequences and the number of amino acid differences per site among sequences are shown along with the standard error in Supplementary Tables 14a–14c. We found that most of the epitopes (belonging to the top eight proteins) were mapped/aligned to the conserved regions. For example, a 15-mer HTL epitope, "TGVSKNGRQLRVSGK" (from DNAJ protein), was found to be completely conserved (100%) across 13 different strains and four species (see Fig. 14 and Supplementary Table 15). Next, a predicted CTL epitope (‘SSDADPTVV’) from trans-sialidase protein sequences was also found to be conserved (Fig. 14). Likewise, we performed epitope conservancy analysis using the IEDB tool and observed that all the predicted epitopes were conserved across different strains of *T. cruzi* (Supplementary Table 16, Supplementary File 4). In addition, we also mapped epitopes (after reverse translation) on genomic sequences of Trypanosoma strains and species to check the conservation at the genomic level (See “Supplementary Website”). Further, we extracted 5750 copies of TS from different proteomes of *T cruzi*. Thereafter, we searched for the presence of epitopes in variants of TS using the Smith Water-Mann algorithm as well as using the IEDB conservancy tool. We found that the epitopes were present in the proteins with varying levels of conservation (See Supplementary Files Y_G and H in Supplementary File 5).

## Discussion

The study reported here comprises a comprehensive approach to utilize informatics and computer algorithms towards the prediction of vaccine targets in pathogens. Our work combines immuno-informatics approaches and reverse vaccinology methods to design an in-silico multi-epitope subunit vaccine that can offer protection against CD. The datasets and frameworks are also used to develop a new machine learning and deep learning system for the prediction of vaccine candidates in general. We have created a resource base for the scientific community working in the area of CD vaccine design [https://tinyurl.com/CDWork800]. We used several strategies to shortlist potential vaccine candidates. The goal was to obtain non-allergenic, antigenic, non-toxic, conserved B-cell, CD8+ and CD4+ epitopes that were assembled into three separate vaccine constructs, V1, V2, and V3. Our major findings include several unique vaccine antigens that are antigenic, immunogenic, and safe (showing no homology with human proteins and the proteome of the gut flora). Further, the designed vaccine constructs are also found to be, theoretically, soluble, thermostable, amenable for expression in model systems, and likely to interact with other proteins. Structurally, the designed constructs show a likelihood of favourable interactions with the TLR-4 on professional antigen-presenting cells. Our vaccine construct consists of epitopes derived from multiple protein molecules (PVCs) which have exhibited the potential to be PVCs in various independent experimental studies. The designed vaccine construct is likely to offer cross-protection since the selected proteins and predicted epitopes used in generating the cocktail vaccine exhibited considerable conservation across the related *Trypanosoma* species/strains.

In the past decade, different research groups have used several strategies ranging from stages of pathogenesis^112^; immunogenic assays^109^, subtractive proteomics^9^, and as well as properties/filters (Supplementary Table 9) to determine candidates for their respective pathogens. Different authors have used different orders of these properties [**P**_**1**_**, P**_**2**_**…. P**_**n**_] as a combined filter to reach the final list of PVCs. Our study explains the impact of the order of applications of these properties on the outcome. Since no proteome-wide studies have been conducted to find the distribution of properties, we decided to apply multiple strategies to rank or filter TC-CLB proteins by randomizing the order, changing the number of filters, etc. For instance, in one of the strategies, we randomized the order of applications for properties [**P**_**n**_] on TC-CLB. In another strategy, we removed the P_1_ [extracellular/secretory] filter which allowed an additional set of proteins [i.e., intracellular] to appear as PVCs. The objective was to screen proteomes diversely to select all best-ranking protein molecules (i.e., PVCs) with desired properties. One of the unique highlights is that we have examined the distribution of different properties across the pathogens’ proteome as well as on positive and control datasets. Further, we also applied Vax-ELAN on recently sequenced Y strain. We observed that top ranking candidates (in both CLB and Y strains) includes TS, Mucin, and Mucin associated surface proteins.

Researchers have initiated several efforts to develop vaccines against CD but issues related to a variety of *T. cruzi* strains, the genetic variability of the host, complex genomic structure^24^, significant phenotypic variation, and variable behaviour of pathogen (in vitro and in vivo) in context of pathophysiology, virulence, tropism, and immunological responses, have created several obstacles^113^. Further, *T. cruzi* is known to be a complex organism with multiple developmental forms with transient expression of different antigens. The problem is compounded by a wide variety of strains, antigenic shifts during different life stages, making proper immunization against the parasite an improbable task. The ability of *T. cruzi* to modulate and evade host immune responses and influence host-parasite interactions allows the parasite to survive through novel mechanisms^114^.

Several vaccine candidates have been reported for CD vaccine development programs across the world. These include Tc24 [and its modified Tc24-C4 derivative], TSA-1, ASP-2, TS, TSSA CD8 epitope, Tc52, TcG1, TcG2, TcG4, TcVac2, TcVac4, and MASP^25^. It is interesting to note that several of these candidates appeared in the final protein list used for our final vaccine construct. In one of the research studies, Michel-Todo et al. extracted *T. cruzi* epitopes from several antigens using publicly available databases^115^. They prioritized a set of epitopes based on sequence conservation criteria, projected population coverage of Latin America population, and biological features using in-silico methods and selected CD8+ T cell, CD4+ T, and B-cell epitopes with < 70% identical to human or human microbiome protein sequences. As a benchmark, we also compared epitopes^115^ with epitopes identified in our study using the VaxiJen tool (Supplementary File-F).

The in-silico approach to design a multi-epitope vaccine construct for Chagas disease presents challenges as a protein-based vaccine given the complexities of producing such candidates as experimental soluble proteins^109^ suitable for scale-up production and purification. However, we have recently embarked upon an mRNA vaccination approach for Chagas disease that might obviate the need for expression and purification steps^116^. We are now working to incorporate the findings here into our mRNA vaccination program.

## Conclusion

Therapeutic interventions for the prevention and elimination of Chagas disease require novel treatment and immunization methods that can protect people at risk and infected populations while providing them with a good quality of life. This study is aimed at developing putative multi-epitope vaccines against CD, a protozoan infection caused by *T. cruzi*. The disease is endemic in Latin America and has impacted other parts of the world. In this study, computational approaches and a reverse vaccinology pipeline were used to screen the complete genomic and proteomic sequences for predicting potential vaccine candidates and designing in-silico chimeric vaccine constructs against the *T. cruzi* CL Brenner. Multiple antigenic B-cell, CD8+, and CD4+ epitopes were assembled into three non-allergenic, antigenic, and non-toxic constructs that can act as a prophylactic potential multi-epitope vaccine construct. Appropriate linkers and adjuvant sequences were also used to enhance the stability, effectiveness, as well as immune response of the engineered vaccine constructs. The designed vaccine construct has suitable structural, physicochemical, and immunological properties which can strongly stimulate both humoral and cellular immune responses in humans. However, experimental validation for efficacy and safety is needed along with pre-clinical studies before human immunization. Planning for such studies in appropriate mouse models of *T. cruzi* and CCC is in progress.

## Supplementary Information

Supplementary Information 1.

Supplementary Information 2.

Supplementary Information 3.

Supplementary Information 4.

Supplementary Information 5.

Supplementary Information 6.

Supplementary Information 7.

Supplementary Information 8.

Supplementary Information 9.

Supplementary Information 10.

Supplementary Information 11.

Supplementary Information 12.

Supplementary Information 13.

Supplementary Information 14.

Supplementary Information 15.

Supplementary Information 16.

Supplementary Information 17.

Supplementary Information 18.

## Data Availability

All raw data were obtained from open sources and have been cited and deposited in Datasets S1 and also available on our website. Supplementary Data: https://tinyurl.com/2b2s927h. Software Pipeline: Vax-ELAN: https://vac.kamalrawal.in/vaxelan/, Vax-ELAN Version 2: https://vac.kamalrawal.in/vaxelan/v2, Vaxi-DL: https://vac.kamalrawal.in/vaxidl/.
