# Supplementary material for: Identification of vaccine targets in pathogens and design of a vaccine using computational approaches
Source: Sci Rep. 2021 Sep 2;11:17626. doi: 10.1038/s41598-021-96863-x (PMC8413327; doi:10.1038/s41598-021-96863-x)
Supplement: Supplementary file 1 — Supplementary Information 1. [file 41598_2021_96863_MOESM1_ESM.docx]

# Identification of vaccine targets in pathogens and design of a vaccine using computational approaches

Kamal Rawal^#1^, Robin Sinha^1^, Bilal Ahmed Abbasi^1^, Amit Chaudhary^1^, Swarsat Kaushik Nath^1^, Priya Kumari^1^, Preeti P.^1^, Devansh Saraf ^1^, Shachee Singh^1^, Kartik Mishra^1^, Pranjay Gupta^1^, Astha Mishra^1^, Trapti Sharma^1^, Srijanee Gupta^1^, Prashant Singh^1^, Shriya Sood^1^, Preeti Subramani^1,^ Aman Kumar Dubey^1^, Ulrich Strych^2^, Peter J. Hotez^2, 3^, Maria Elena Bottazzi^2, 3^

1. Amity Institute of Biotechnology, Amity University Uttar Pradesh, India.
2. Texas Children’s Hospital Center for Vaccine Development, Departments of Pediatrics
   and Molecular Virology and Microbiology, National School of Tropical Medicine,
   Baylor College of Medicine, Houston, TX, USA.
3. Department of Biology, Baylor University, Waco, Texas, USA.

#Corresponding Author

Email ID: kamal.rawal@gmail.com

Centre for Computational Biology and Bioinformatics, AIB

Amity University, Noida.

| **Supplementary Figures** | | |
| --- | --- | --- |
| **Supplementary Figures** | **Description** | **Page No.** |
| **Supplementary Figure 1** | Strategies with relevant characteristics (features)used in Vax-Elan (Draw.io- <https://www.diagrams.net/-14.6.15> ). | **3** |
| **Supplementary Figure 2** | Computation of the cut-off values for ROC curve (Microsoft Office 2016-<https://www.microsoft.com/en-in/microsoft-365/word> ). | **4** |
| **Supplementary Figure 3a** | Frequency distribution of P_i_ values based on the results from the positive and negative datasets of bacteria. The Y-axis represents sequence count. The X-axis represents the *P_i_ score values for each sequence. Blue colour depicts non-antigen and red antigen sequences.  *P_i_ stands for probability value where P_i_= S_i_/N | **4** |
| **Supplementary Figure 3b** | Frequency distribution of P_i_ values based on the results from the positive and negative datasets of fungi. The Y-axis represents sequence count. The X-axis represents the *P_i_ score values for each sequence. Blue colour depicts non-antigen and red antigen sequences.  *P_i_ stands for probability value where P_i_= S_i_/N | **5** |
| **Supplementary Figure 3c** | Frequency distribution of P_i_ values based on the results from the positive and negative datasets of protozoa. The Y-axis represents sequence count. The X-axis represents the *P_i_ score values for each sequence. Blue colour depicts non-antigen and red antigen sequences.  *P_i_ stands for probability value where P_i_= S_i_/N | **5** |
| **Supplementary Figure 3d** | Frequency distribution of P_i_ values based on the results from the positive and negative datasets of viruses. The Y-axis represents sequence count. The X-axis represents the *P_i_ score values for each sequence. Blue colour depicts non-antigen and red antigen sequences.  *P_i_ stands for probability value where P_i_= S_i_/N | **6** |
| **Supplementary Figure 4** | Strategy-1 (Vax-ELAN) (Microsoft Office 2016-<https://www.microsoft.com/en-in/microsoft-365/word> ). | **6-7** |


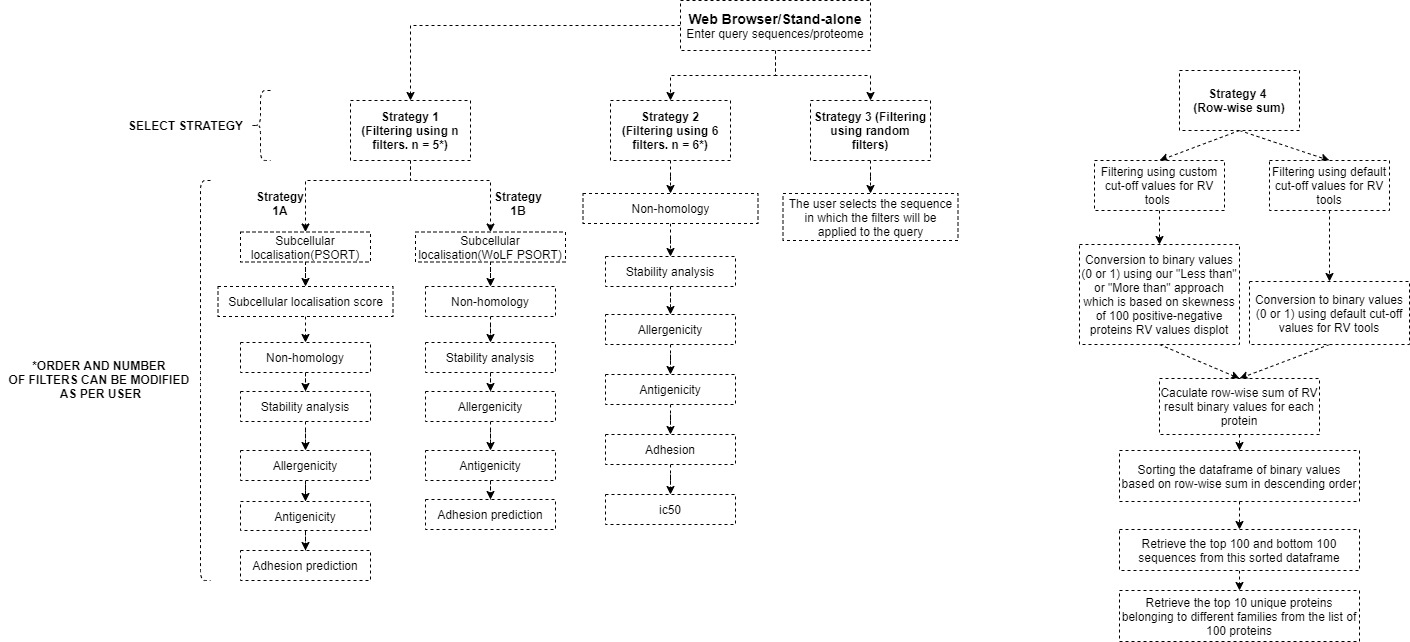


**Supplementary Figure 1:**  Strategies with relevant characteristics (features) used in Vax-Elan (Draw.io- <https://www.diagrams.net/-14.6.15> )


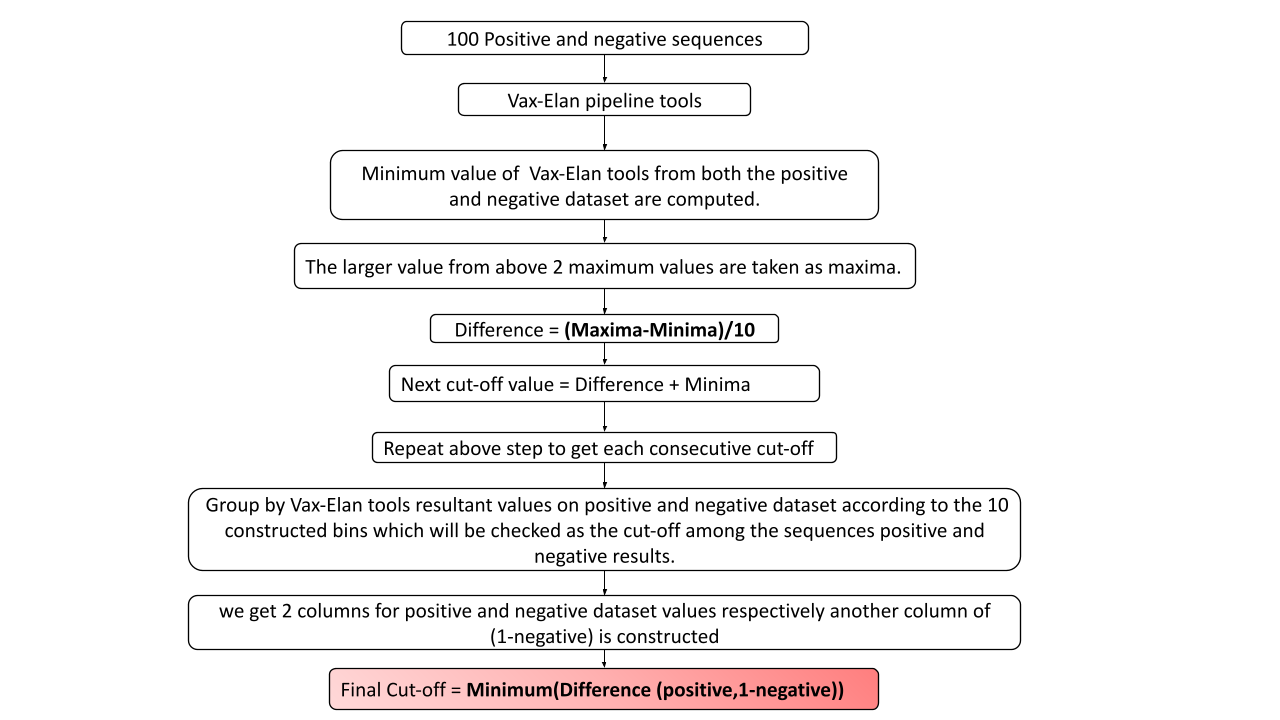


**Supplementary Figure 2**: Computation of the cut-off values for ROC curve (Microsoft Office 2016-<https://www.microsoft.com/en-in/microsoft-365/word> ).


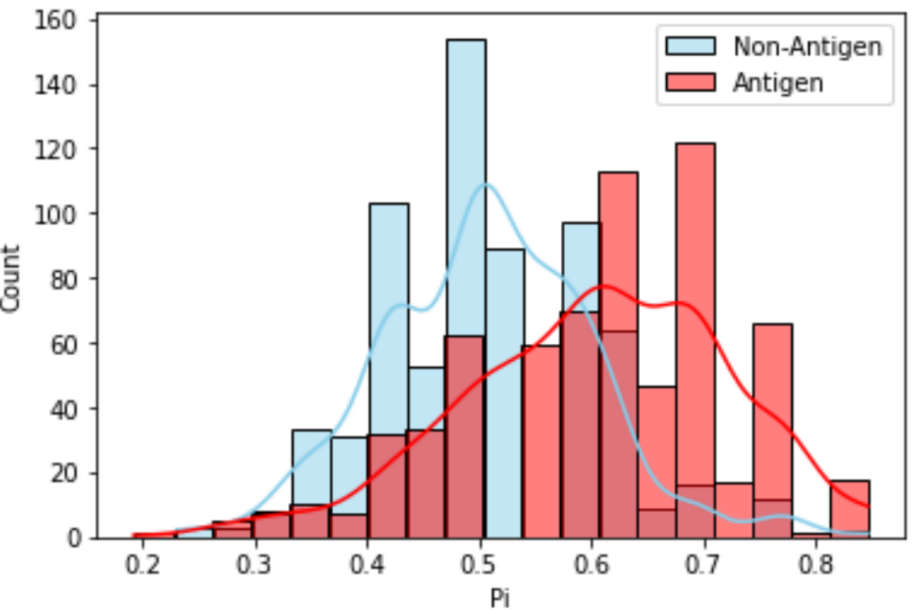


**Supplementary Figure 3a:** Frequency distribution of P_i_ values based on the results from the positive and negative datasets of bacteria. The Y-axis represents sequence count. The X-axis represents the *P_i_ score values for each sequence. Blue colour depicts non-antigen and red antigen sequences.

*P_i_ stands for probability value where P_i_= S_i_/N


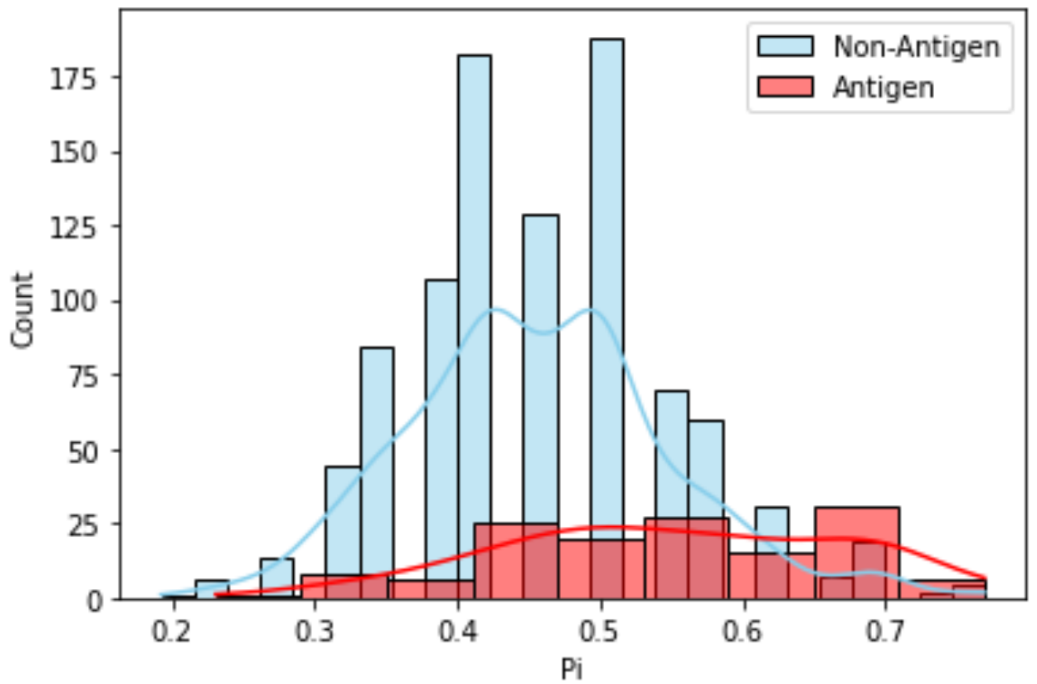


**Supplementary Figure 3b:** Frequency distribution of P_i_ values based on the results from the positive and negative datasets of fungi. The Y-axis represents sequence count. The X-axis represents the *P_i_ score values for each sequence. Blue colour depicts non-antigen and red antigen sequences.

*P_i_ stands for probability value where P_i_= S_i_/N


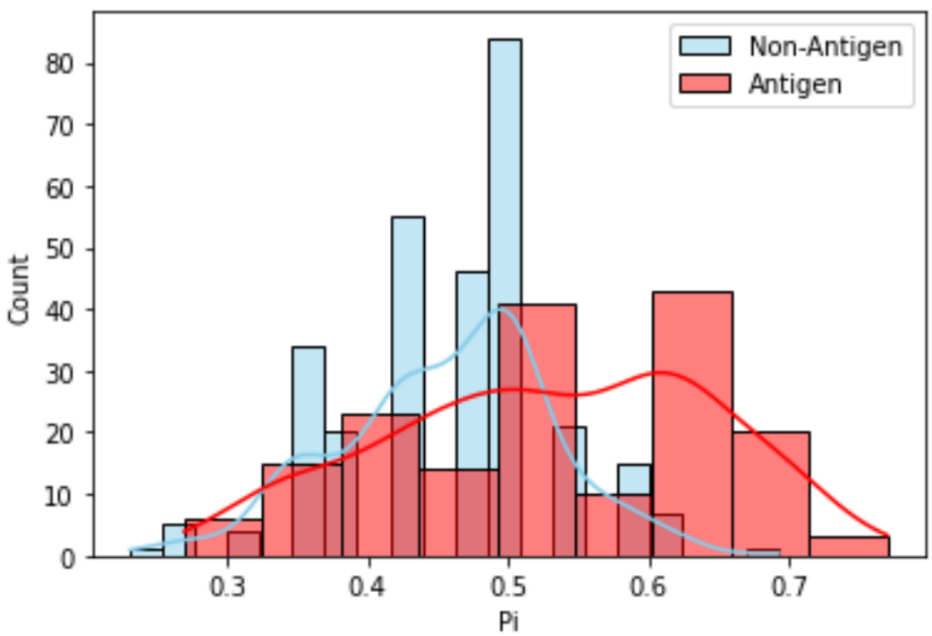


**Supplementary Figure 3c:** Frequency distribution of P_i_ values based on the results from the positive and negative datasets of protozoa. The Y-axis represents sequence count. The X-axis represents the *P_i_ score values for each sequence. Blue colour depicts non-antigen and red antigen sequences.

*P_i_ stands for probability value where P_i_= S_i_/N


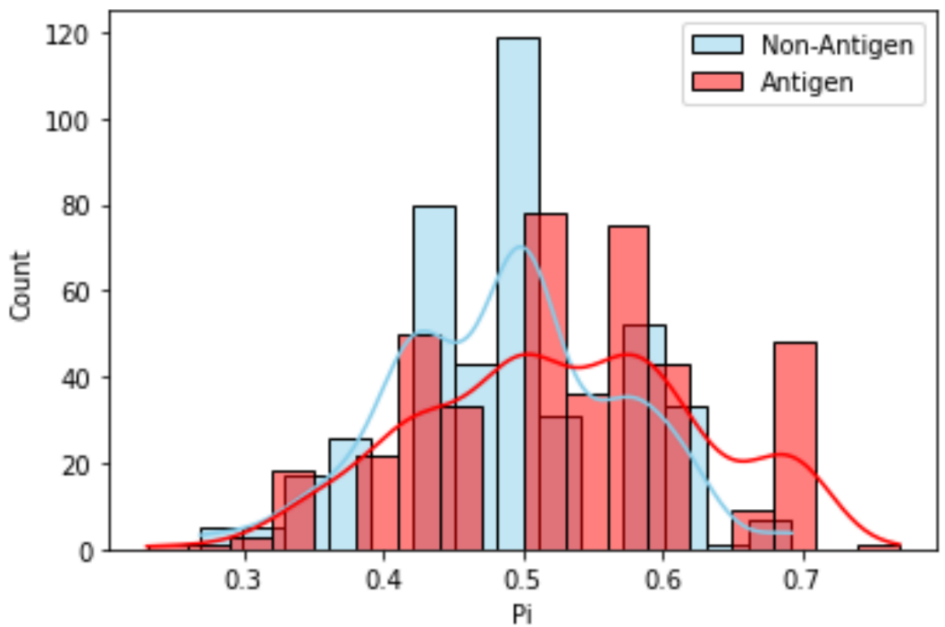


**Supplementary Figure 3d:** Frequency distribution of P_i_ values based on the results from the positive and negative datasets of viruses. The Y-axis represents sequence count. The X-axis represents the *P_i_ score values for each sequence. Blue colour depicts non-antigen and red antigen sequences.

*P_i_ stands for probability value where P_i_= S_i_/N

**Supplementary Figure 4: Strategy-1 (Vax-ELAN)**

In this experiment, we used 7 filters starting from subcellular localization as first screening point. PsortB^1^ tool and python scripts were used to filter those proteins which were predicted to be as ­­not extracellular or secretory in nature. In the next step, we have utilised the default threshold score defined by the tool itself as an additional filter. At the third level, we have executed blastp search against human proteome to find non-homologous proteins. This step was followed by the evaluation of proteins on the basis of stability to exclude unstable proteins. Next, we used blastp for allergenicity analysis. Further, we applied VaxiJen 2.0 for filtering non-antigenic proteins having a value less than 0.5. In the final step, we have applied FungalRV to shortlist those proteins having adhesive characteristics to be selected as a potential vaccine candidate.


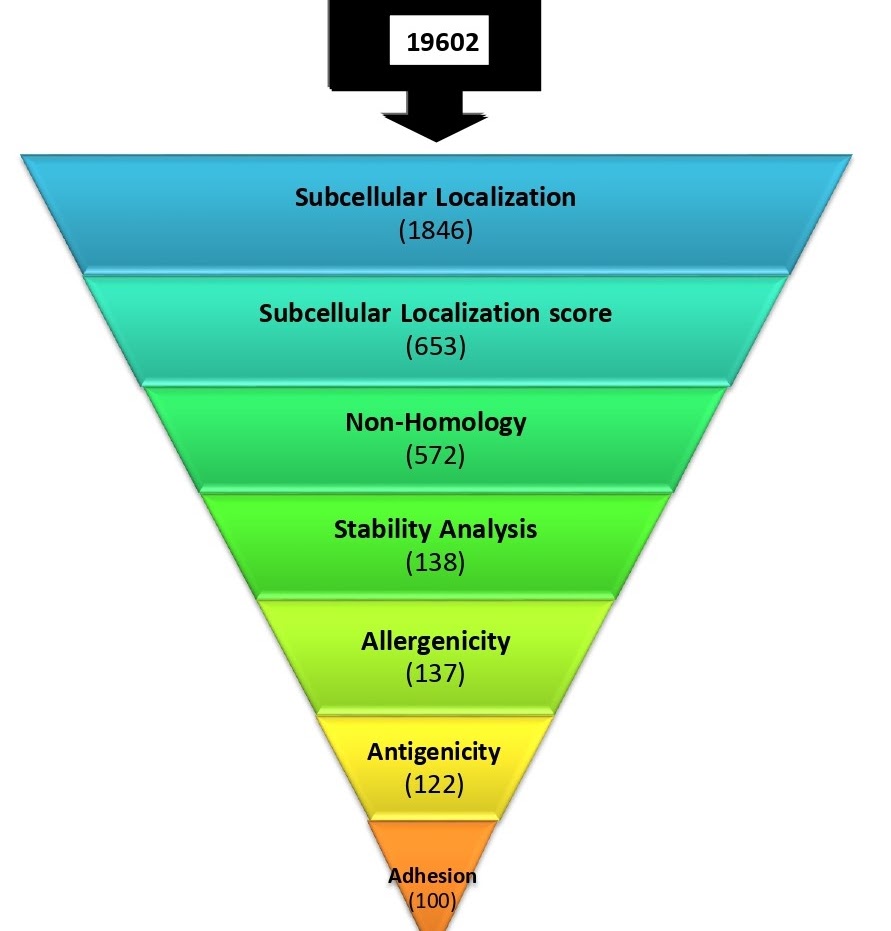


**Fig 4A:** Workflow adopted for determining PVC via strategy 1(Microsoft Office 2016-<https://www.microsoft.com/en-in/microsoft-365/word> ).


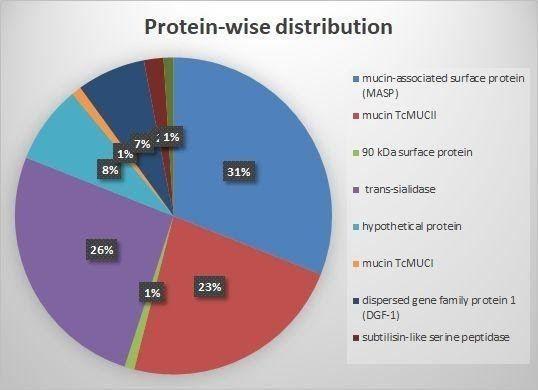


**Fig 4B:** Percentage distribution of protein superfamilies obtained in the top 100 proteins.
