# Supplementary material for: Identification of vaccine targets in pathogens and design of a vaccine using computational approaches
Source: Sci Rep. 2021 Sep 2;11:17626. doi: 10.1038/s41598-021-96863-x (PMC8413327; doi:10.1038/s41598-021-96863-x)
Supplement: Supplementary file 2 — Supplementary Information 2. [file 41598_2021_96863_MOESM2_ESM.zip › Supplementary Files/Supplementary File 5/Supplementary Files.docx]

# **Identification of vaccine targets in pathogens and design of a vaccine using computational approaches**

Kamal Rawal^#1^, Robin Sinha^1^, Bilal Ahmed Abbasi^1^, Amit Chaudhary^1^, Swarsat Kaushik Nath^1^, Priya Kumari^1^, Preeti P.^1^, Devansh Saraf^1^, Shachee Singh^1^, Kartik Mishra^1^, Pranjay Gupta^1^, Astha Mishra^1^, Trapti Sharma^1^, Srijanee Gupta^1^, Prashant Singh^1^, Shriya Sood^1^, Preeti Subramani^1,^ Aman Kumar Dubey^1^, Ulrich Strych^2^, Peter J. Hotez^2, 3^, Maria Elena Bottazzi^2, 3^

1. Amity Institute of Biotechnology, Amity University Uttar Pradesh, India.
2. Texas Children’s Hospital Center for Vaccine Development, Departments of Pediatrics
    and Molecular Virology and Microbiology, National School of Tropical Medicine,
    Baylor College of Medicine, Houston, TX, USA.
3. Department of Biology, Baylor University, Waco, Texas, USA.

#Corresponding Author

Email ID: kamal.rawal@gmail.com

Centre for Computational Biology and Bioinformatics, AIB

Amity University, Noida.

**Keywords:** Bioinformatics, Reverse vaccinology, Vaccine Development, Artificial Intelligence

**Supplementary Data**:<https://tinyurl.com/CDWork800>

**Software Pipeline**:

Vax-ELAN:<https://vac.kamalrawal.in/vaxelan/>

Vax-ELAN Version 2:<https://vac.kamalrawal.in/vaxelan/v2>

Vaxi-DL: <https://vac.kamalrawal.in/vaxidl/>

| **Supplementary Files** | **Captions** |
| --- | --- |
| Supplementary File Y_A | Whole-genome sequence of *T. cruzi* (strain Y) |
| Supplementary File Y_B | Shortlist 3772 proteins using strategy 1B |
| Supplementary File Y_C | Binary file for strategy 4 used to shortlist proteins based on Pi score |
| Supplementary File Y_D | Top 500 sequence IDs based on Si/Pi score |
| Supplementary File Y_E | Comparison between top 10 proteins of 2 different strains (CLB & Y) Acquired by applying strategies 1B and 4 |
| Supplementary File Y_F | The expression of vaccine construct (V1) in *Saccharomyces cerevisiae* and *Pichia pastoris* |
| Supplementary File Y_G | Epitope conservancy analysis in different strains for trans-sialidase protein (IEDB analysis resource) |
| Supplementary File Y_H | Epitope conservancy analysis in different strains for trans-sialidase protein (Smith-Waterman alignment approach) |
| Supplementary File Y_I | P-Distance of different strains (Trans-sialidase protein) |
