# Supplementary material for: Identification of vaccine targets in pathogens and design of a vaccine using computational approaches
Source: Sci Rep. 2021 Sep 2;11:17626. doi: 10.1038/s41598-021-96863-x (PMC8413327; doi:10.1038/s41598-021-96863-x)
Supplement: Supplementary file 2 — Supplementary Information 2. [file 41598_2021_96863_MOESM2_ESM.zip › Supplementary Files/Supplementary File 5/Supplementary -Y.docx]

# **Identification of vaccine targets in pathogens and design of a vaccine using computational approaches**

Kamal Rawal^#1^, Robin Sinha^1^, Bilal Ahmed Abbasi^1^, Amit Chaudhary^1^, Swarsat Kaushik Nath^1^, Priya Kumari^1^, Preeti P.^1^, Devansh Saraf^1^, Shachee Singh^1^, Kartik Mishra^1^, Pranjay Gupta^1^, Astha Mishra^1^, Trapti Sharma^1^, Srijanee Gupta^1^, Prashant Singh^1^, Shriya Sood^1^, Preeti Subramani^1,^ Aman Kumar Dubey^1^, Ulrich Strych^2^, Peter J. Hotez^2, 3^, Maria Elena Bottazzi^2, 3^

1. Amity Institute of Biotechnology, Amity University Uttar Pradesh, India.
2. Texas Children’s Hospital Center for Vaccine Development, Departments of Pediatrics
   and Molecular Virology and Microbiology, National School of Tropical Medicine,
   Baylor College of Medicine, Houston, TX, USA.
3. Department of Biology, Baylor University, Waco, Texas, USA.

#Corresponding Author

Email ID: kamal.rawal@gmail.com

Centre for Computational Biology and Bioinformatics, AIB

Amity University, Noida.

**Keywords:** Bioinformatics, Reverse vaccinology, Vaccine Development, Artificial Intelligence

**Supplementary Data**: <https://tinyurl.com/CDWork800>

**Software Pipeline**:

Vax-ELAN: <https://vac.kamalrawal.in/vaxelan/>

Vax-ELAN Version 2: <https://vac.kamalrawal.in/vaxelan/v2>

Vaxi-DL: <https://vac.kamalrawal.in/vaxidl/>

**INDEX**

| S.No. | Title | Page No. |
| --- | --- | --- |
| 1. | **INTRODUCTION** | 3 |
| 2. | **METHODOLOGY** | 3-4 |
|  | Retrieval of genome and proteome sequences |  |
|  | Strategy - ORF-based screening of Y strain |  |
|  | Strategies adopted for protein filtering |  |
|  | Conversion of Proteins’ Feature/Property Values into Binary Values |  |
|  | Comparison of different strategies to find top ranking proteins |  |
|  | Codon optimization of the chimeric protein |  |
|  | Evaluation of genetic diversity |  |
| 3. | **RESULTS** | 4-7 |
|  | Identification of Subcellular Location of the Proteins |  |
|  | Identification of Y strain proteins that are non-homologous to human proteins |  |
|  | Instability Analysis |  |
|  | Non-Allergenicity Analysis |  |
|  | Adhesion Prediction |  |
|  | Shortlisted Protein Vaccine Candidates (PVCs) |  |
|  | Codon optimization of the chimeric protein |  |
|  | Evaluation of genetic diversity |  |
| 4. | **REFERENCES** | 7 |

**INTRODUCTION**

*Trypanosoma cruzi* Y strain (TC-Y) genome was sequenced using Illumina MiSeq series^1^.

**METHODOLOGY:**

**Retrieval of genome and proteome sequences:**

The whole-genome sequence of *T. cruzi* (strain Y) (**Supplementary Table Y_A**) was obtained from NCBI (Accession ID: NMZO00000000).

**Strategy - ORF-based screening of Y strain:**

We used Prodigal^2^ to predict 38,653 ORFs (see website). Further, we removed duplicates and obtained 38218 ORFs in the genome. Next, the predicted ORFs (after translation) were subjected for evaluation using the VAX-ELAN pipeline. Firstly, in strategy 1(B), we applied bioinformatics tools such as WoLF PSORT^3^, BLAST, ProtParam, VaxiJen and Fungal RV (in that order) to filter proteins.

Secondly, we screened the total proteome using all the Vax-ELAN tools (Strategy 4). After different levels of filtering, we shortlisted 3,772 top proteins in strategy 1B [(](https://drive.google.com/file/d/1U2phBnUIllsNrdqzoYGDIznWv8D9emm5/view?usp=sharing)**Supplementary File Y_**[B](https://drive.google.com/file/d/1U2phBnUIllsNrdqzoYGDIznWv8D9emm5/view?usp=sharing)). We also shortlisted top 10 proteins based on their S_i_ score (**Supplementary File Y_**[C](https://docs.google.com/spreadsheets/d/1gD5cyVb7KhOqBjls1DbytDJaNczhxcHUiD4escoC3tU/edit#gid=311146063)). The function of top-ranking proteins was determined using the BLAST tool. Next, we identified evidence from literature for the top 10 proteins.

**Strategies adopted for protein filtering:**

In strategy 1, we included WoLF PSORT^3^ as an additional tool for finding subcellular locations apart from PSORTB. Thus, strategy 1 was categorized into two sub-categories: 1A (which uses PSORTB in the first level of filtering) and 1B (uses WoLF PSORTB for filtering). To illustrate strategy 1B, we used the BLASTp tool to evaluate non-homology with human proteins. Subsequently, we used ProtParam to compute the stability of proteins, succeeded by a BLASTp search against the allergen database to filter non-allergen proteins. Furthermore, we used FungalRV to predict adhesion molecule-like properties. This strategy generated a set of potential vaccine candidates. Additionally, we filtered the proteins using strategy 4 as well to generate top ranking hits (See, (**Supplementary File Y_C**).

**Conversion of Proteins’ Feature/Property Values into Binary Values:**

By applying strategy 4, we evaluated 38612 proteins of Y strain. A row-wise sum corresponding to all the properties [i.e., total score] was computed for Y strain proteins. Thereafter, all the proteins of the Y strain were ranked according to the total score (S_i_ or P_i_). Finally, we shortlisted the top 10 unique hits based upon the Pi score. ([**Supplementary File Y_D**](https://docs.google.com/spreadsheets/d/1FhXilnE25X1vilrZ8mVC7A6c0pfFcqnG/edit#gid=150299772)).

**Comparison of different strategies to find top ranking proteins:**

We collected the top-ranking hits from different strategies and used python-based programs to find common and unique proteins (See, **Supplementary File Y_E**). Shortlisted proteins reported from multiple strategies were used in subsequent steps such as epitope prediction and vaccine construction.

**RESULTS**

**Identification of Subcellular Location of the Proteins:**

Using the WoLF PSORT tool, we screened 38218 proteins (out of 38653, after removing duplicates) of the reference proteome of Y strain [Accession ID: NMZO00000000] and found that 23,540 proteins were predicted to be localized in the plasma membrane and outer membrane of the cell.

### **Identification of Y strain proteins that are non-homologous to human proteins:**

### To prevent undesired cross-reactivity of vaccines with the human host, the proposed vaccine candidate must be different from human proteins. Therefore, we used BLASTp and found 23,153 proteins (out of 23,540) to be non-homologous to human proteins.

**Stability Analysis:**

Protein stability is of crucial importance for the efficient presentation of antigenic peptides on MHC, which plays a decisive role in triggering strong immune reactions. Using ProtParam, the protein instability index was determined and proteins having an Instability Index (II) less than 40 were selected. This led to shortlisting of 8169 proteins (out of 23,153) that were predicted to be stable.

**Non-Allergenicity Analysis:**

To find out non-allergenic proteins in our list, we performed a BLASTp search against the Allergen Online database and found 8164 proteins (out of 8169) to be non-allergenic.

**Antigenicity Prediction:**

To check the antigenicity of the filtered-out protein, we performed the analysis using Vaxijen with a threshold value of greater or equal to 0.4 and found 6695 proteins (out of 8164) to be antigenic.

**Adhesion Prediction:**

Next, we performed adhesion prediction using FungalRV with a threshold value of greater or equal to -1.2. Several studies have shown that adhesins are vital in initiating pathogen-based infections. Therefore, it seemed practical to target these proteins for vaccine development. A total of 3,772 proteins (out of 7069) were predicted to possess desired properties similar to adhesin proteins. We used these top 3,772 proteins for subsequent analysis as a filtered list. It was also found that multiple hits belonging to the multi-gene/protein family such as trans-sialidases, and mucin-associated surface protein were present in the top 3,772 list.

**Shortlisted Protein Vaccine Candidates (PVCs):**

**Strategy 1B:**

The top 3,772 shortlisted proteins were analysed further to evaluate the presence of additional criteria (TM α-helices, signal peptides, essentiality, and virulence) to narrow down the best ten proteins as PVCs. These include hypothetical protein, trans-sialidase, putative dispersed gene family protein 1 (DGF-1), ATPase, Mucin-associated surface protein (MASP), putative Serine/threonine-protein kinase NEK10, surface protease GP63, mucin-like glycoprotein, mucin TcMUCII and beta galactofuranosyl glycosyltransferase

Next, we compared the shortlisted proteins in TC-Y and TC-CLB. The proteins that were common in both the strains were hypothetical protein, trans-sialidase, putative dispersed gene family protein 1 (DGF-1), Mucin-associated surface protein (MASP), Surface Protease GP63 and Mucin TcMUCII (**Supplementary File Y_E**).

**Strategy 4:**

The final top 10 proteins with highest Si scores in TC-Y strain were found to be putative mucin TcMUCII, trans-sialidase, Mucin-associated surface protein (MASP) subgroup S022, hypothetical protein ECC02_011937, 90 kDa surface protein, surface protease GP63, putative dispersed gene family protein 1 (DGF-1), surface protein-1, ATPase and putative phosphatidylserine decarboxylase.

Out of the shortlisted 10 proteins of TC-Y strain, we found that putative mucin TcMUCII , trans-sialidase, Mucin-associated surface protein (MASP) subgroup S022, hypothetical protein, 90 kDa surface protein were also present in top 10 hits of CLB as well (**Supplementary File Y_E**).

The subsequent steps which include epitope prediction, construction of chimeric protein etc are described on our supplementary website.

**Evaluation of genetic diversity**

We also performed evolutionary analysis on different strains of *T.cruzi* for Trans-sialidase protein (**Supplementary File Y_I**). We extracted 4 copies of trans-sialidase from the Y strain. The average evolutionary distance among multiple copies of the Y strain was found to be 0.879.

For epitope conservancy analysis, we applied 2 approaches. The first set of data was retrieved from NCBI for the 12 previously studied strains along with the Y strain. First, we analysed our data using the IEDB analysis resource (<http://tools.iedb.org/conservancy/> ).

For instance, upon epitope conservancy analysis on Y strain trans-sialidase copies, we found the identity ranging from 42.86% to 57.14% for BCL, 33.33% to 55.56% for CTL, and 26.67% to 33.33% for HTL epitope (**Supplementary File Y_G)**.

In our second approach, we applied the Smith-Waterman algorithm for local alignment to align the epitopes with the conserved region of the sequences. For Y, the identity ranged from 56% to 80% for BCL, 50% to 83% for CTL, and 62% to 100% for HTL epitope ([Supplementary File Y_H](https://docs.google.com/spreadsheets/d/1oauQzHcc15-NX0FFgBGsUTbZNwgGQ4PYy3qAW9QRGos/edit#gid=447318787)) .

**References**

1. Callejas-Hernández, F., Gutierrez-Nogues, Á., Rastrojo, A., Gironès, N. & Fresno, M. Analysis of mRNA processing at whole transcriptome level, transcriptomic profile and genome sequence refinement of Trypanosoma cruzi. *Sci. Rep.* **9**, 1–11 (2019).

2. Hyatt, D. *et al.* Prodigal: Prokaryotic gene recognition and translation initiation site identification. *BMC Bioinformatics* **11**, 119 (2010).

3. Horton, P. *et al.* WoLF PSORT: protein localization predictor. *Nucleic Acids Res.* **35**, W585–W587 (2007).
