# Supplementary material for: Identification of vaccine targets in pathogens and design of a vaccine using computational approaches
Source: Sci Rep. 2021 Sep 2;11:17626. doi: 10.1038/s41598-021-96863-x (PMC8413327; doi:10.1038/s41598-021-96863-x)
Supplement: Supplementary file 2 — Supplementary Information 2. [file 41598_2021_96863_MOESM2_ESM.zip › Supplementary Files/Supplementary File 5/Supplmentary File Y_F.docx]

**Supplementary File Y_F: The expression of vaccine construct (V1) in *Saccharomyces cerevisiae* and *Pichia pastoris***

In case of *Saccharomyces cerevisiae,* Java Codon Adaptation Tool or JCat server [[http://www.jcat.de](http://www.jcat.de/)] was employed for codon optimisation of designed vaccine construct (V1). JCAT results revealed that the optimized codon sequence has a length of 1,218 nucleotides and its CAI (Codon Adaptation Index) was predicted to be 0.93 (**see Fig A**), with an average of 44.66% GC for the adapted sequence. These values indicate a stable expression of the designed vaccine construct in the selected microbial host. For optimal gene expression, SnapGene software was employed, the designed chimeric protein sequence was integrated into the *E. coli* pET-28a [+] vector and *Saccharomyces cerevisiae* pRS416 phagemid vector by incorporating restriction sites which were followed by cloning into the vector using published methods (**see Fig. B & C**).


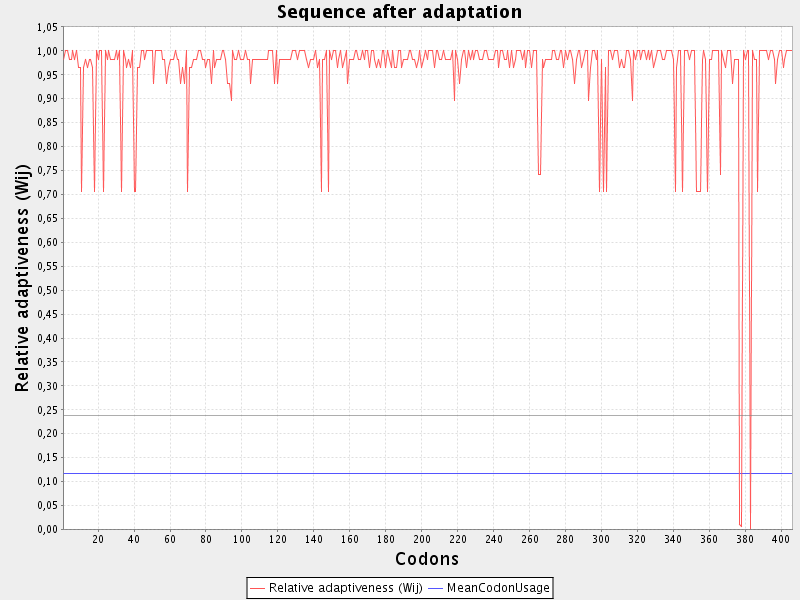


**Figure A:** Optimized codons of designed vaccine construct (VI) in *Saccharomyces cerevisiae*


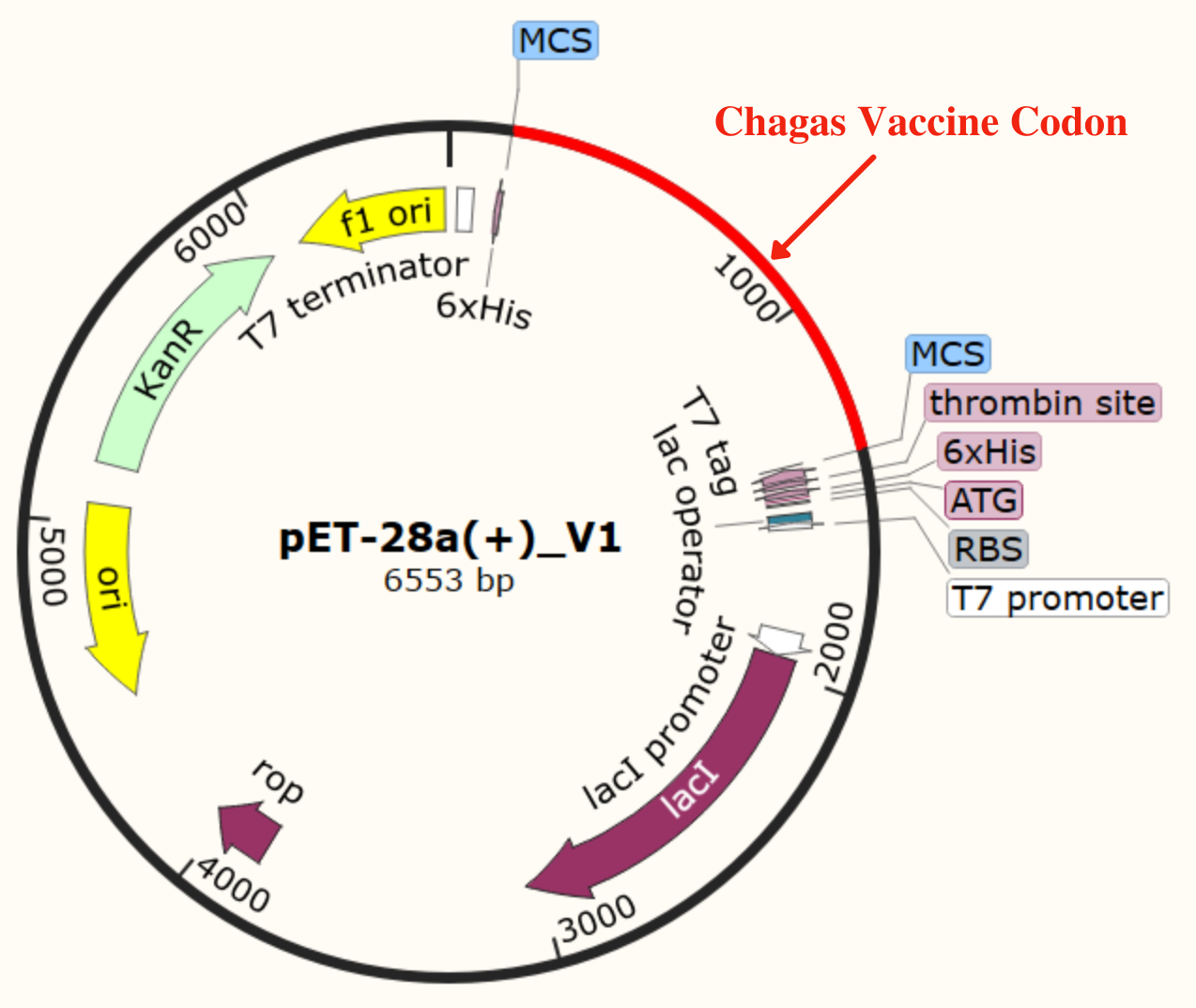


**Figure B:** *In-silico* cloning of the chimeric protein sequence into the *E. coli* pET-28a [+] vector .

**
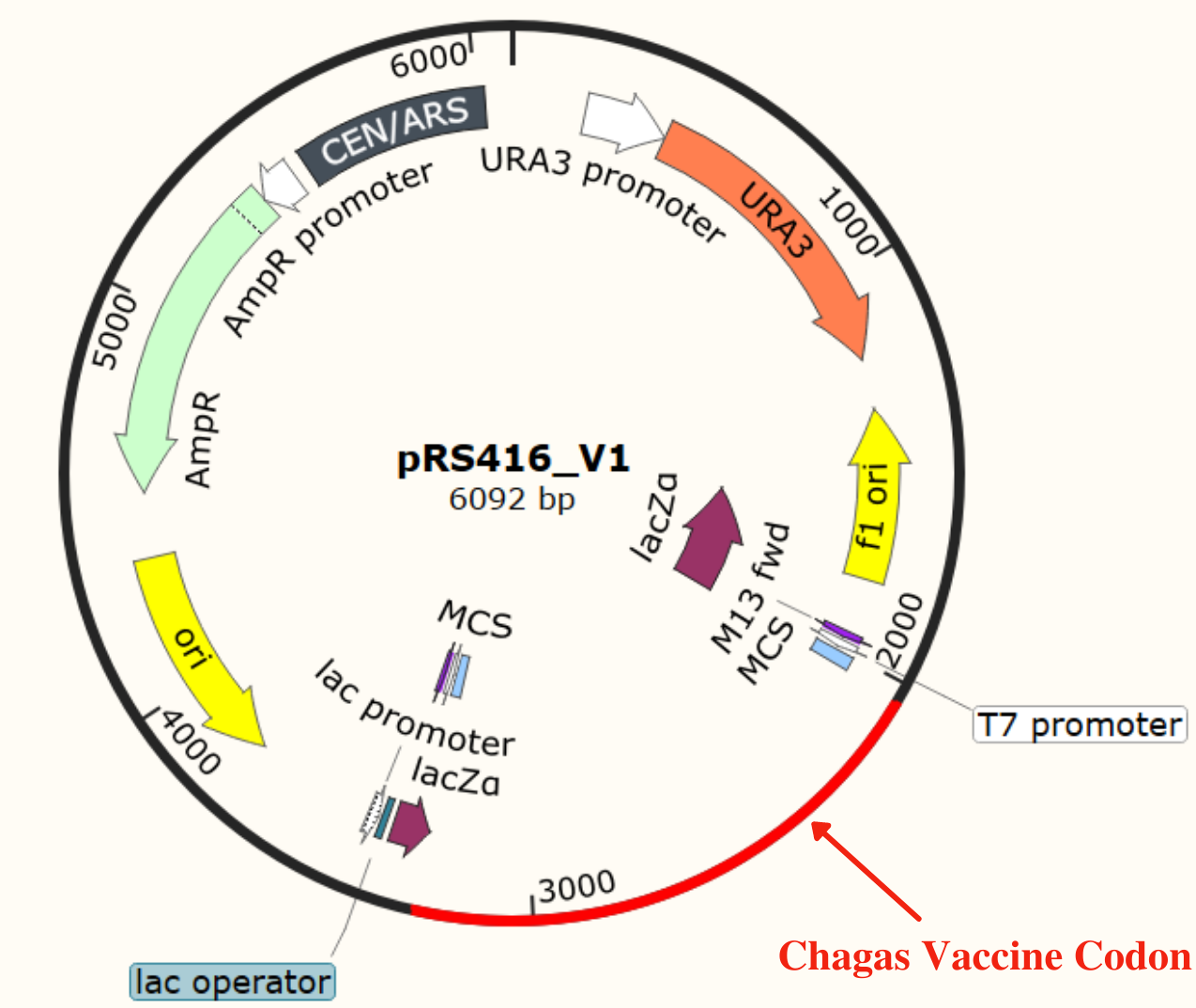
**

**Figure C:** *In-silico* cloning of the chimeric protein sequence in the *Saccharomyces cerevisiae* pRS416 phagemid vector.

Numerous recombinant protein antigens against diseases such as Chagas’ disease (*Trypanosoma cruzi*) have been synthesized in *Pichia pastoris* for use in developing human subunit vaccines (Bill, 2015). In one such experiment, Fontanella et al., 2008 immunized mice with an engineered mutant *trans*-sialidase expressed in *P. pastoris.* JCat server doesn’t have the option of *Pichia pastoris* to select as an option for expression host organisms. In order to overcome this shortcoming, we employed Genscript Rare Codon Analysis tool [<https://www.genscript.com/tools/rare-codon-analysis>] for codon optimization of the designed vaccine construct. Gencript results revealed that the optimized codon sequence has a length of 1,218 nucleotides and its CAI (Codon Adaptation Index) was predicted to be 0.96 (**see Fig. D**), with an average of 44.81% GC (**see Fig. E**) for the adapted sequence. These values indicate a stable expression of the designed vaccine construct in the selected microbial host. For optimal gene expression, SnapGene software was employed, the designed chimeric protein sequence was integrated into the E. coli pET-28a [+] vector and *Saccharomyces cerevisiae* pRS416 phagemid vector by incorporating restriction sites which were followed by cloning into the vector using published methods (**see Fig. B & C**).


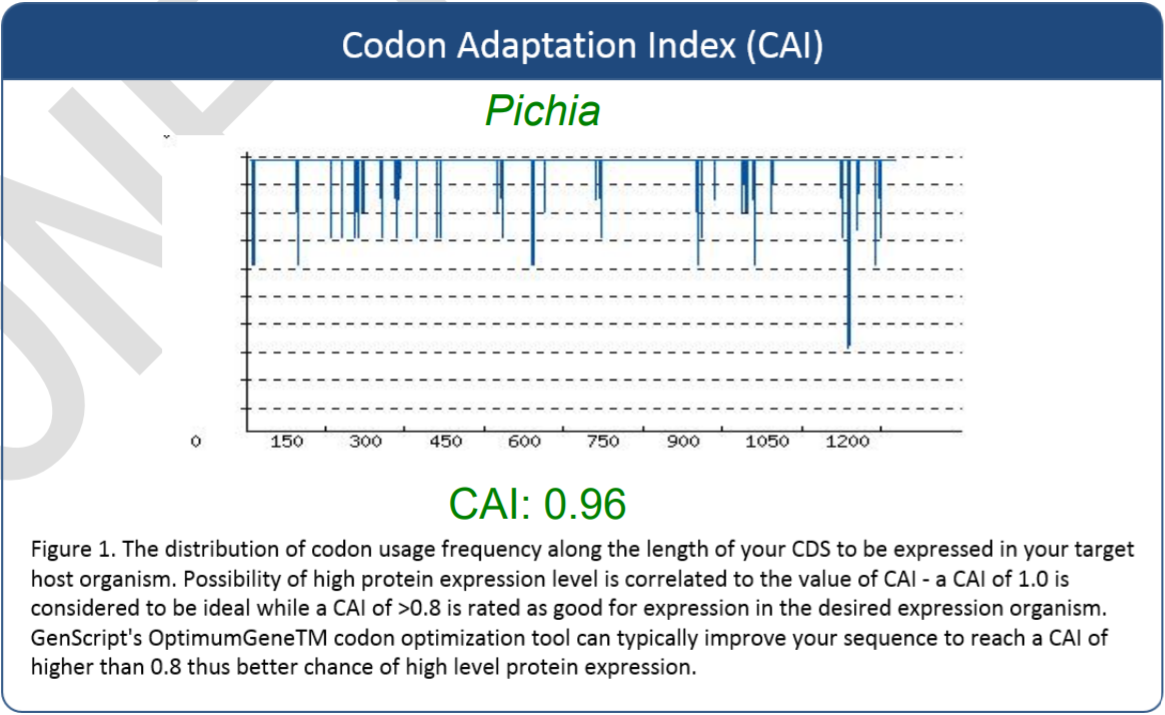


**Figure D:** The graph showing Codon Adaptation Index (CAI) of designed vaccine construct.

**
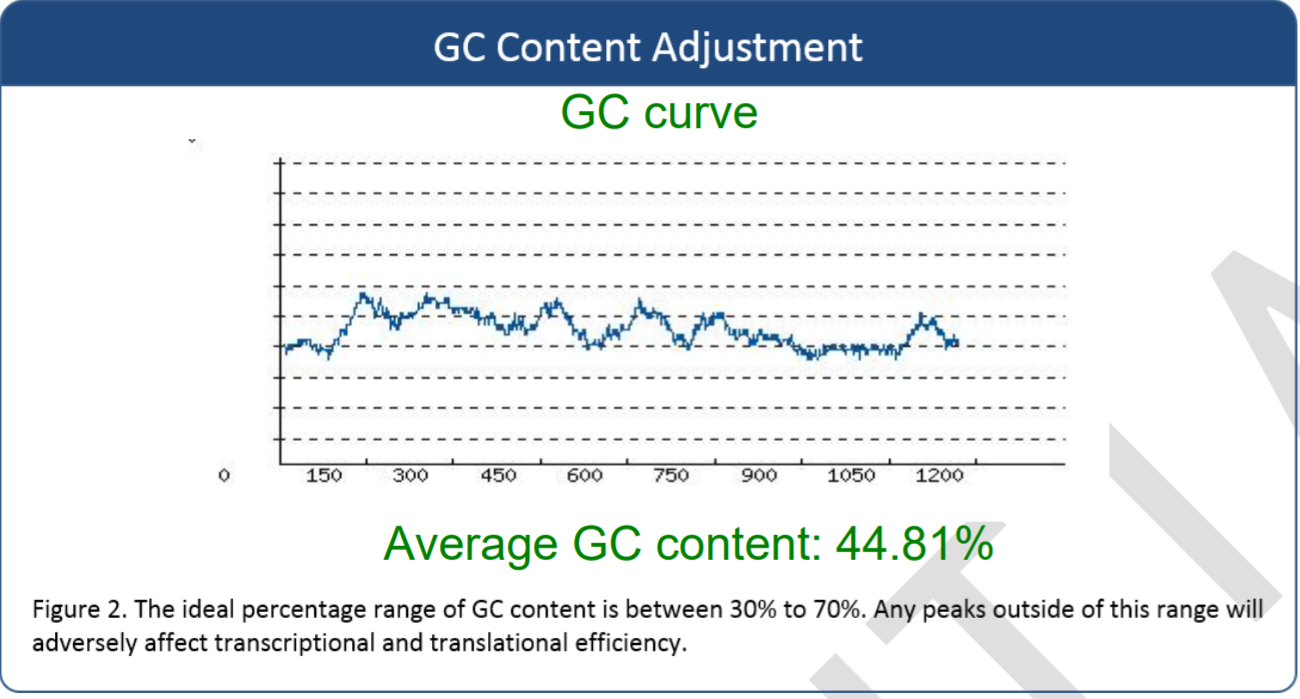
**

**Figure E:** The graph of average of GC content of designed vaccine construct.

**References**
1. Bill, R. M. (2015). Recombinant protein subunit vaccine synthesis in microbes: a role for yeast?. Journal of Pharmacy and Pharmacology, 67(3), 319-328.

2. Fontanella, G. H., De Vusser, K., Laroy, W., Daurelio, L., Nocito, A. L., Revelli, S., & Contreras, R. (2008). Immunization with an engineered mutant trans-sialidase highly protects mice from experimental Trypanosoma cruzi infection: a vaccine candidate. Vaccine, 26(19), 2322-2334.
