# Supplementary material for: Identification of vaccine targets in pathogens and design of a vaccine using computational approaches
Source: Sci Rep. 2021 Sep 2;11:17626. doi: 10.1038/s41598-021-96863-x (PMC8413327; doi:10.1038/s41598-021-96863-x)
Supplement: Supplementary file 2 — Supplementary Information 2. [file 41598_2021_96863_MOESM2_ESM.zip › Supplementary Files/Supplementary Files.docx]

# **Identification of vaccine targets in pathogens and design of a vaccine using computational approaches**

Kamal Rawal^#1^, Robin Sinha^1^, Bilal Ahmed Abbasi^1^, Amit Chaudhary^1^, Swarsat Kaushik Nath^1^, Priya Kumari^1^, Preeti P.^1^, Devansh Saraf ^1^, Shachee Singh^1^, Kartik Mishra^1^, Pranjay Gupta^1^, Astha Mishra^1^, Trapti Sharma^1^, Srijanee Gupta^1^, Prashant Singh^1^, Shriya Sood^1^, Preeti Subramani^1,^ Aman Kumar Dubey^1^, Ulrich Strych^2^, Peter J. Hotez^2, 3^, Maria Elena Bottazzi^2, 3^

1. Amity Institute of Biotechnology, Amity University Uttar Pradesh, India.
2. Texas Children’s Hospital Center for Vaccine Development, Departments of Pediatrics
   and Molecular Virology and Microbiology, National School of Tropical Medicine,
   Baylor College of Medicine, Houston, TX, USA.
3. Department of Biology, Baylor University, Waco, Texas, USA.

#Corresponding Author

Email ID: kamal.rawal@gmail.com

Centre for Computational Biology and Bioinformatics, AIB

Amity University, Noida.

| **Supplementary Files** | |  |
| --- | --- | --- |
| **S.No.** | **Captions** | **Page No.** |
| **Supplementary File 1** | Shortlisted vaccine target of different pathogens | See folder |
| **Supplementary File 2** | Positive dataset of bacterial antigen sequences | See folder |
| **Supplementary File 3** | Vaccine candidates from Interspecies and inter-strain of Trypanosoma | See folder |
| **Supplementary File 4** | Conservancy analysis of epitopes across different species and strains of *Trypanosoma cruzi* | See folder |
| **Supplementary File 5** | The supplementary files for *Trypanosoma cruzi* Y strain | See folder |
| **Supplementary File-A** | Alternate Strategies for PVCs Predictions | 3-9 |
| **Supplementary File-B** | Comparison of different strategies to find top ranking proteins | 10-11 |
| **Supplementary File-C** | Identification of B cell Epitope sequences | 12 |
| **Supplementary File-D** | Identification of CTL Epitope sequences | 13 |
| **Supplementary File-E** | Identification of HTL Epitope sequences | 14 |
| **Supplementary File-F** | Experiment with Michel-Todo’s work | 15-19 |

**Supplementary File-A**: **Alternate Strategies for PVCs Predictions**

We also implemented several alternative approaches to identify the potential vaccine candidates to design vaccine construct in order to select additional PVCs (i.e., improve diversity) which might get filtered in Strategy 1. These approaches were formulated based upon previously reported methodologies to identify PVCs. In the subsequent sections, we shall explain these approaches:

**>Strategy 2**

It includes filtering of TC-CLB proteome without the use of sub-cellular localization and sub-cellular localization score filters. The purpose was to include those proteins which are not extracellular or secretory in nature (**Fig. C1**).

**
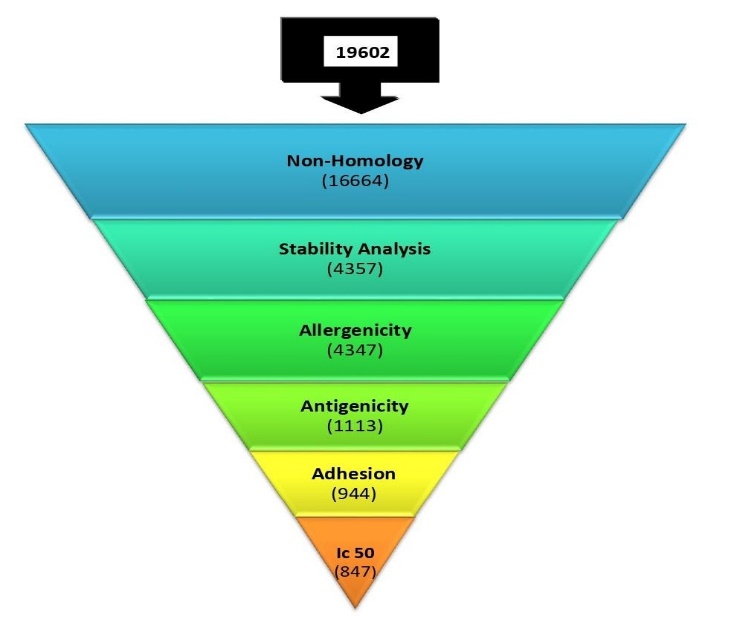
**

**Fig A1:** Workflow adopted for determining PVC via strategy 2(Microsoft Office 2016-<https://www.microsoft.com/en-in/microsoft-365/word> ).

The final filtered set of PVC along with intermediate file and Top PVC are displayed in the following link:

<https://tinyurl.com/y3ass9ec>

**>Strategy 3:**

Here, we applied various filters in random order to screen vaccine candidates. Random filtering of proteins (non-sequentially) was performed to identify desired protein candidates.


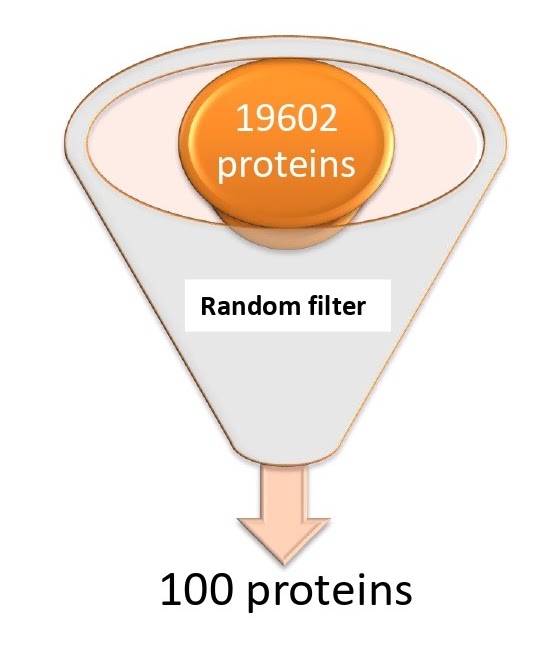


**Fig A2:** Workflow adopted for determining PVC via strategy 3(Microsoft Office 2016-<https://www.microsoft.com/en-in/microsoft-365/word> ).

In order to implement, we developed scripts which shuffles the order of application of filters (See list of filters, **Fig A1**) on the TC-CLB proteome. This experiment also finds out the effect of order of application of filters on final outcome. The final set of PVCs obtained using this approach along with intermediate files as well as top ranking PVCs are present at the web-link:

<https://tinyurl.com/y2ay48c3>

**>Strategy 4:**

In this strategy, we used predefined thresholds **(See, Supplementary Table 6)** to transform scores (or values, labels etc. which were determined using 21 different tools; (See, [Tcruzi_clbrener_final_file.xlsx)](https://drive.google.com/file/d/1BD5m_y7tVR0H1HpWfDdIuubNbHDMXbyK/view) of 19,602 proteins. The output file (See, [Cl Brener 19k Protein Binary](https://drive.google.com/file/d/1N9cRs_MNJmjoyMhvi0OdBLcCE70WOqzZ/view)) contains binary score (0 or 1) corresponding to different properties **(See Supplementary Table 3)** against each protein.


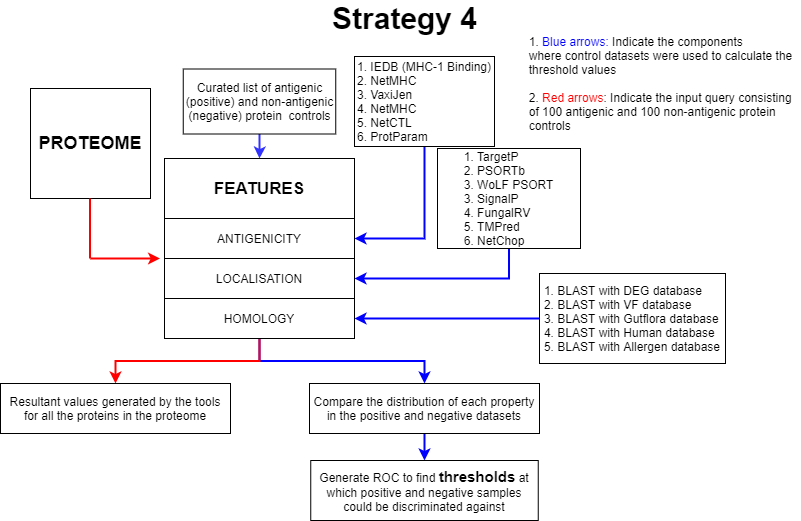


**Fig A3:** Workflow adopted for determining PVC via strategy 4(Draw.io- <https://www.diagrams.net/-14.6.15> ).

**Procedure to Determine Thresholds/Cut-offs:**

Here, we generated distributions of scores corresponding to each property for positive examples (100) as well as for control (100) (See: [A](https://drive.google.com/file/d/1SS7yAj6iddG3I41qxrn2tsxS8UKvFp03/view), [A1](https://drive.google.com/file/u/0/d/1ckPM7UUJqiiqkul0qDcCSd85Msy3TfC7/view) and [B](https://drive.google.com/file/d/1KkICJeoxeM-2q-FbGqjR1HRHdJV8q0CO/view)). The characteristics of distribution (i.e., peak location, kurtosis etc.) enabled us to determine the cut-offs. For instance, in context of “secretory nature using TargetP” property, we find that location of distribution of scores for positive dataset are present on “relatively on far right” (i.e. greater mean) when compared with distribution of control datasets (less mean score). This intuitively suggests that population of positive examples would occupy certain range of scores on X axis therefore, any new protein which has scores (for secretory nature) shall be labelled as positive when it shows similarities with positive dataset (See, [B](https://drive.google.com/file/d/1KkICJeoxeM-2q-FbGqjR1HRHdJV8q0CO/view)) and vice versa.

After, we converted properties of each protein into binary values (0 or 1), we also computed row wise sum for these binary values of 19602 proteins (See, [Sorted Cl Brener 19k Protein Binary Dataset with row-wise sum](https://drive.google.com/file/d/1HJG1vNTldZYwMbMEcvyVQqMk1TRneoyY/view)). Therefore, now the TCCLB protein can be visualised as vector (1, 0, 1, 0, 0….) as well as row wise sum total (S). Next, we ranked these proteins based upon the ‘S’. Top hundred proteins (i.e. having maximum ones/”S”) were selected in this strategy.

**Generation of cut-off for all parameters** -

We used 100 protein sequences belonging to vaccine candidates (labelled as positive dataset) and 100 protein sequences (control/negative dataset) to generate cut-offs. First, we evaluated sequences from positive dataset using tool(s) (corresponding to each parameter See**, Supplementary Table B).** An array of values was constructed comprising values generated by each tool for each protein sequence.  The maxima and minima were determined for each tool in the array. Secondly, we computed 10 bins for each tool using the difference as **(maxima-minima)/10.**

Third, A range of values were generated using this difference (Initially, first value was generated using the sum of minima and the difference and this step was followed until maxima value was reached). For instance, the first cut-off was the minima (See Figure: C2). Fourth, we used 10 different cut-offs to find out sensitivity parameters on 100 positive examples. Similar studies were conducted on negative examples and curves were plotted (See Figure C2, shown in red). The intersection points of these curves were considered as threshold value (See Figure C3). For example, for FungalRV, we found the optimal threshold as -1.477. At this threshold, sensitivity and specificity values were optimal.


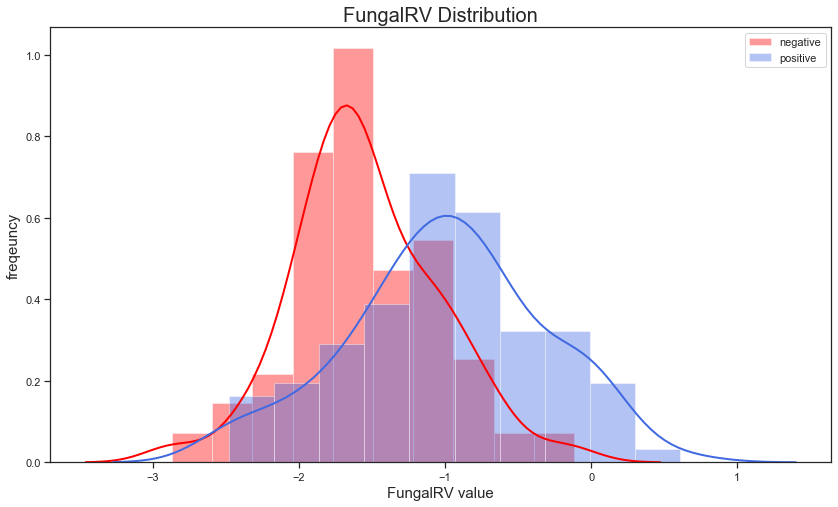


**Fig A4:** The distributions of scores generated by FungalRV tool has been displayed in the figure for positive (light blue) and negative (pink) protein sequence examples. The Y axis represent proportion of protein sequences. The X axis represent the FungalRV score values.


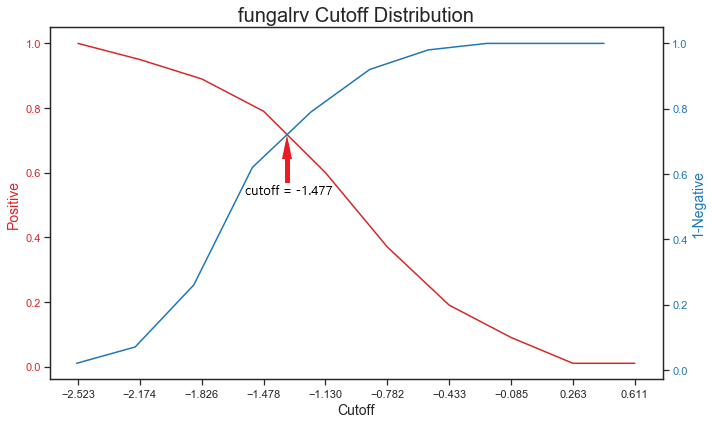


**Fig A5:** The figure shows mechanism to determine threshold (cut-off) for the given property (i.e., FungalRV). X axis denotes the cut-offs (obtained from FungalRV scores for positive and negative datasets) and Y axis shows proportion of positive and negative examples which are able to cross the given threshold. The cut-off was changed at each step leading to change in performance parameters (i.e., sensitivity as well as specificity). The performance parameters were computed for the positive and negative datasets at each step (i.e., cut-offs). The FungalRV value (on X axis) corresponding to intersection point was considered as threshold since the sensitivity and specificity values were maximised at that point.

The final filtered set of PVC along with intermediate file and top PVCs are available in the following link:

<https://tinyurl.com/y5axfths>

**ADDITIONAL DATA:**


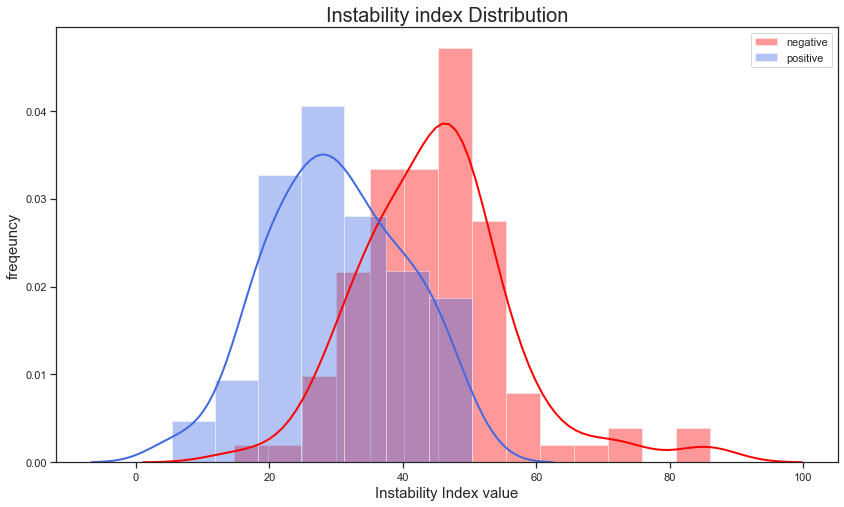


**Fig A4:** Distributions of scores for positive and negative dataset for the parameter named instability index.


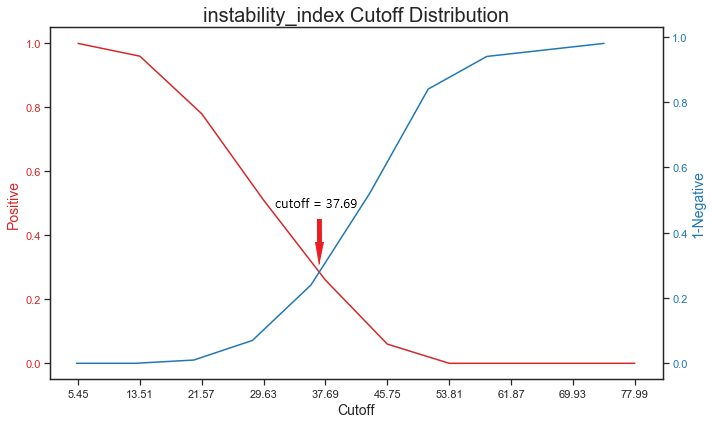


**Fig A5:** Mechanism to determine threshold for a given parameter (Instability index).


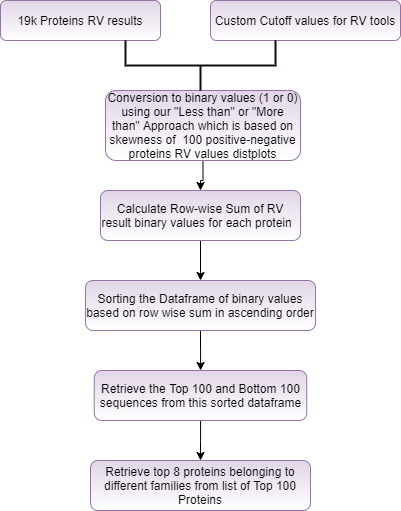


**Fig. A6:** Workflow for filtering of top 100 proteins (Draw.io- <https://www.diagrams.net/-14.6.15> ).

**Supporting files:**

<https://drive.google.com/file/d/1_wJisTWfGn3XK9289AlUdNdD363ZCcFA/view?usp=sharing>

<https://drive.google.com/file/d/1WfSFlDCFzFbNGyXLPgZEdmZZzs29m1ca/view?usp=sharing>

<https://drive.google.com/file/d/10MhiiqnEmJbKGXzf90bAvBWUexBwwE_d/view?usp=sharing>

<https://drive.google.com/file/d/1QxK4LNHkjNeHUEs4d1BH1b4cgvZXPt-1/view?usp=sharing>

**>Strategy 5:**

The scaffold WGS (NW_001849447.1) was downloaded from NCBI. We predicted 121349 ORFs using PRODIGAL tool. These ORFs subjected to the RV pipeline using Strategy-1 to shortlist the ORF (1640).

The final filtered set of PVC along with intermediate files and top-ranking PVCs are available at: <https://tinyurl.com/y5xadd2t>

**Supplementary File-B:** **Comparison of different strategies to find top ranking proteins**

The top-ranking hits from different strategies were collected and python-based programs were used to find common and unique proteins. Shortlisted proteins reported from multiple strategies were used in subsequent steps such as epitope prediction and vaccine construction.

## **Table B1:** The comparison made between the strategies is aimed at identifying unique and common epitopes across all the strategies.

| Strategies | Motivation |
| --- | --- |
| **Strategy 1:** | A series of bioinformatics filters which have resulted into 100 proteins. |
| **Strategy 2:** | A series of bioinformatics filters excluding sub-cellular localization tool (PSORTb score and localization) |
| **Strategy 3:** | Random shuffling of series of application of bioinformatics tools. |
| **Strategy 4:** | Binary representation of features and selecting top-ranked protein candidates. |
| **Strategy 5:** | Genome to ORF prediction via Prodigal tool and subjected to series of bioinformatics tools excluding few(allergen) |

| Strategies | Strategy | Conclusion/Results Achieved |
| --- | --- | --- |
| Strategy-I | **7 filters.** | 100 proteins, In which top 8 highest unique antigenic proteins were selected to make subunit vaccine. (Main Research work) |
| Strategy-II | **No PsortB filters** | 847 proteins after removing **PSortB.** Top ranking proteins (See, [CD website](https://tinyurl.com/y2xedhrv)). |
| Strategy-III | **Strategy-I (shuffled)** | 100 proteins, In which 8 highest antigenic proteins were selected to make subunit vaccine. We find that the top-ranking proteins were similar to results obtained through **Strategy I.** |
| Strategy-IV | **Binary, Summation and Ranking approach** | (Unique) 8 proteins having highest feature score **(across the row)** were shared to website. |
| Strategy-V | **Genome to ORF, 7 filters.** | 121,349 ORF were filtered to **1640** ORF; top 100 ORFs & top 8 ORFs were shortlisted (See website- Link) |

**Table B2:** Summary of results based upon different strategies.

## **Identification of common and unique proteins by comparison of different strategies:**

## **Strategy 1** Vs **2:**

## We find that the mucin-associated surface protein (MASP - XP_809166.1) & trans-sialidase (XP_818708.1) were found to shortlisted by both approaches.

## **Strategy 1 Vs 3:**

## As mentioned above, Strategies 1 and 3 comprise the same filters, therefore, they have resulted in the same set of shortlisted proteins.

## **Strategy 1 Vs 4:**

There were no common proteins.

## **Strategy 1 vs 5:**

## No proteins were identified as common protein vaccine candidates.

**Table B3:** Binary Comparison of conservation of unique proteins filtered using Strategy-1 in other strategies. Only top 100 proteins identified by each strategy were studied.

| **Top Proteins (unique) after filtration** | **Strategy I** | **Strategy II** | **Strategy III** | **Strategy IV** | **Strategy V** |
| --- | --- | --- | --- | --- | --- |
| XP_813527.1 (DGF-1) | 1 | 0 | 1 | 0 | 0 |
| XP_809835.1 (subtilin-like serine peptidase) | 1 | 0 | 1 | 0 | 0 |
| XP_806816.1 (DNAJ Chaperone protein) | 1 | 0 | 1 | 0 | 0 |
| XP_809166.1 (MASP) | 1 | 1 | 1 | 0 | 0 |
| XP_816522.1 (Mucin TcMUCII) | 1 | 1 | 1 | 0 | 1 |
| XP_818708.1(Trans-sialidase) | 1 | 0 | 1 | 0 | 0 |
| XP_815016.1 (Surface Protein) | 1 | 0 | 1 | 0 | 0 |
| XP_821916.1 (hypothetical protein) | 1 | 0 | 1 | 0 | 0 |

The top 100 proteins obtained from each of the strategy can be accessed at the following link:

[<https://drive.google.com/file/d/1qn1607n2suvQm-EIDq2qb71Djx7VDJAx/view?usp=sharing>].

**Supplementary File-C**: Identification of B-cell Epitope sequences.

We have used various online available bioinformatics tools for Prediction of linear B-cell epitopes including BcePred^1^, ABCPred^2^ and BepiPred^3^. The predicted epitopes by each tool were further evaluated based on their antigenicity and immunogenicity. The final list of epitopes predicted by each tool are shown in:

<https://drive.google.com/file/d/1-gYeqEpnZZK-l5np9dPM5wCIx5KMg8Vm/view>


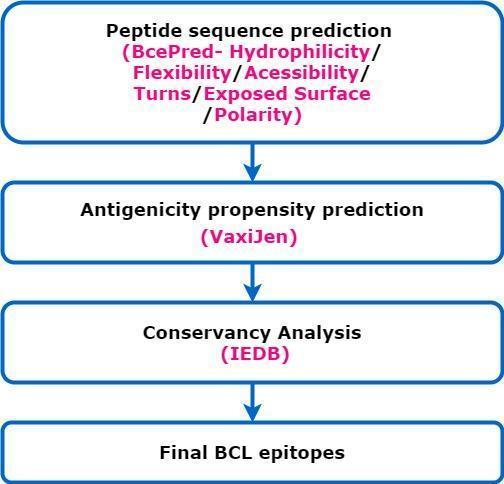


**Fig C1:** Flow chart illustrating workflow for selection of B-cell epitope sequences (Draw.io- <https://www.diagrams.net/-14.6.15> ).

**References:**

1. Saha, S. and Raghava, G.P.S., 2004, September. BcePred: prediction of continuous B-cell epitopes in antigenic sequences using physico-chemical properties. In *International Conference on Artificial Immune Systems* (pp. 197-204). Springer, Berlin, Heidelberg.
2. Saha, S. and Raghava, G.P.S., 2006. Prediction of continuous B‐cell epitopes in an antigen using recurrent neural network. Proteins: Structure, Function, and Bioinformatics, 65(1), pp.40-48.
3. Jespersen, M.C., Peters, B., Nielsen, M. and Marcatili, P., 2017. BepiPred-2.0: improving sequence-based B-cell epitope prediction using conformational epitopes. Nucleic acids research, 45(W1), pp. W24-W29.

**Supplementary File-D**: Identification of Cytotoxic T- lymphocyte Epitope sequences.

We have used various online available bioinformatics tools for prediction of CTL epitopes including NetMHC^1^, EpiJen^2^, Propred1^3^ and NetCTL^4^. The predicted epitopes by each tool were further selected based on their antigenicity and immunogenicity. The final list of epitopes are available at following link:

<https://drive.google.com/file/d/1etN8gzG_Zifi_L_7Xx0pa3DU1YgBrqEy/view>


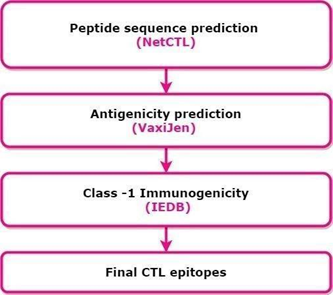


**Fig D1:** Flow chart illustrating workflow for selecting Cytotoxic-T-Lymphocyte epitope sequences From NetCTL (Draw.io- <https://www.diagrams.net/-14.6.15> ).

**References:**

1. Lundegaard, C., Lamberth, K., Harndahl, M., Buus, S., Lund, O., & Nielsen, M. (2008). NetMHC-3.0: accurate web accessible predictions of human, mouse and monkey MHC class I affinities for peptides of length 8–11. *Nucleic acids research*, *36*(suppl_2), W509-W512.
2. Doytchinova, I. A., Guan, P., & Flower, D. R. (2006). EpiJen: a server for multistep T cell epitope prediction. *BMC bioinformatics*, *7*(1), 131.
3. Singh, H., & Raghava, G. P. S. (2003). ProPred1: prediction of promiscuous MHC Class-I binding sites. *Bioinformatics*, *19*(8), 1009-1014.
4. Larsen, M. V., Lundegaard, C., Lamberth, K., Buus, S., Lund, O., & Nielsen, M. (2007). Large-scale validation of methods for cytotoxic T-lymphocyte epitope prediction. *BMC bioinformatics*, *8*(1), 424.

**Supplementary** **File-E**: Identification of HTL epitope sequences

We have used various online available bioinformatics tools for prediction of linear B-cell epitopes including IEDB MHC-2^1^, MHC2PRED^2^ and Propred^3.^ The predicted epitopes by each tool were further filtered based on their antigenicity, allergenicity, and toxicity and IFN gamma induction analysis. The intermediate data and final list are available at the following link:

<https://drive.google.com/file/d/1WuzncnVW4GirbHvSXg5keX8U2sg95N2R/view>


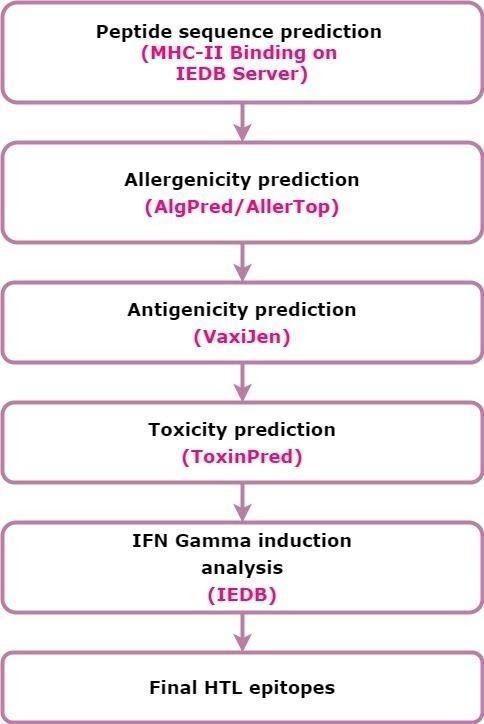


**Fig E1:** Flow chart illustrating workflow for selecting Helper-T-Lymphocyte epitope sequences (Draw.io- <https://www.diagrams.net/-14.6.15> ).

**References:**

1. Wang, P., Sidney, J., Kim, Y., Sette, A., Lund, O., Nielsen, M. and Peters, B., 2010. Peptide binding predictions for HLA DR, DP and DQ molecules. BMC bioinformatics, 11(1), p.568.
2. Lata, S., Bhasin, M. and Raghava, G.P., 2007. Application of machine learning techniques in predicting MHC binders. In Immunoinformatics (pp. 201-215). Humana Press.
3. Singh, H. and Raghava, G.P.S., 2001. ProPred: prediction of HLA-DR binding sites. Bioinformatics, 17(12), pp.1236-1237.

**Supplementary File-F**: Comparative analysis with predictions generated by Michel-Todo et al 2019.

In this study, we compared the epitopes identified by Michel-Todo et al., (2019) in developing vaccine against Chagas disease. The top-scoring antigenic epitopes from our study and Michel-Todo et al are shown in Table C1.

|  | Michel-Todo et al. 2019 | | Our study | |
| --- | --- | --- | --- | --- |
|  | Epitopes | Highest Antigenic score | Epitopes | Highest Antigenic score |
| CD4+(HTL) | DDELFHYFLWTFFFIDLLYAVM | 1.60 | TGVSKNGRQLRVSGK | 1.79 |
| B cell | EGESRHRTRSGSARHHRRHHRNEAGG | 1.70 | GSCGCRC | 3.51 |
| CD8+(CTL) | FPFCWLPTY | 2.31 | DAALLGGDY | 2.09 |

We computed the mean antigenic scores of epitopes predicted by Michel-Todo et al 2019^1^ and found that mean antigenic scores of Michel-Todo was comparatively lesser than that of scores of our epitopes.

**Table F1:** Table showing the highest antigenic scoring epitopes from both the studies.

**Figure F1:** Graphical representation of the antigenic score used by Michel-Todo et al 2019 versus epitopes used by us for constructing a vaccine against Chagas disease.

**Table F2:** B cell epitopes vaccine component (Michel-Todo et al).

| **Epitopes** | **Protein Family** | **Accession no** | **Antigenic score** |
| --- | --- | --- | --- |
| GTDEGLLLPVDNDGDESS | Lipophosphoglycan biosynthetic protein/HSP90 superfamily | XP_818651.1 | 0.94  (Probable ANTIGEN) |
| QNKKFFADKPDESTL | Kinetoplastid membrane protein 11 (KMP11) | XP_810488.1 | 1.07 (Probable ANTIGEN) |
| EGESRHRTRSGSARHHRRHHRNEAGG | Hypothetical protein conserved | XP_814578.1 | 1.70  (Probable ANTIGEN) |
| RRRRHSRSKRGEEDGGET | Hypothetical protein conserved | XP_814578.1 | 1.64  (Probable ANTIGEN) |
| GTPSRTTGRSTSTTRGVSRPTNGVTPSTSLAHRASTPGRTGTRSTTPSRSSVLS | Associated kinase of Tb14-3-3 putative | XP_819464.1 | 0.81  (Probable ANTIGEN) |
| GVCTSAEPRDLLDPVALCMPYPGAERIIG | Associated kinase of Tb14-3-3putative | XP_819464.1 | 0.16 (Probable NON-ANTIGEN) |
| NSQETPDQQKTGITRV | Associated kinase of Tb14-3-3putative | XP_819464.1 | 0.19 (Probable NON-ANTIGEN) |
| REARGTSTPRRAETPSGGSRVRGA | Associated kinase of Tb14-3-3putative | XP_819464.1 | 0.79  (Probable ANTIGEN) |
| KRSRSHNDGPARKRRRKDNRP | Methyl-transferase putative | XP_803963 | 0.75  (Probable ANTIGEN) |
| NPSASPEASWQLNQSWNPL | Hypothetical protein conserved | XP_809003.1 | -0.33  (Probable NON-ANTIGEN) |
|  |  |  | Mean=0.71 |

**Table F3**: List of B cell epitopes in predicted proteins and their antigens predicted in our study.

| **S. No.** | **Protein ID** | **Top BCL Epitopes** | **Antigenic Propensity** |
| --- | --- | --- | --- |
| 1 | XP_813527.1 (DGF-1) | GSCGCRC | 3.51 (Probable ANTIGEN) |
| 2 | XP_809835.1 (subtilisin-like serine peptidase) | PLLLFVFF | 3.06(Probable ANTIGEN) |
| 3 | XP_806816.1 (DNAJ Chaperone protein) | VHINLKQ | 1.49 (Probable ANTIGEN) |
| 4 | XP_809166.1 (MASP) | TSPLFPLLLVVAC | 1.23 (Probable ANTIGEN) |
| 5 | XP_816522.1 (Mucin TcMUCII) | MTCRLLCALLVLALCCCPSVCVT | 0.77 (Probable ANTIGEN) |
| 6 | XP_818708.1 (Trans-sialidase) | SLWSVRL | 1.61 (Probable ANTIGEN) |
| 7 | XP_815016.1 (Surface Protein) | DVPPSSLP | 0.89 (Probable ANTIGEN) |
| 8 | XP_821916.1 (hypothetical protein) | EKPQCLLLSSGILVDVLMR | 1.15 (Probable ANTIGEN) |
|  |  |  | **Mean=1.71** |

**Table F4:** Michel-Todo CD8+ T cell epitopes vaccine component.

| **Antigen id** | **Protein family** | **Epitopes** | **Vaxijen score** |
| --- | --- | --- | --- |
| XP_807293.1 | Phosphoglycerate mutase | VYGRFYYRF | 1.68 (Probable ANTIGEN) |
| XP_811816.1 | Hypothetical protein | RFFPSVFWR | 1.46 (Probable ANTIGEN) |
| XP_806973.1 | Hypothetical protein | FPFCWLPTY | 2.31 (Probable ANTIGEN) |
| PWV07436 | Hypothetical protein – C3747_101g9 | MPAFQGWA | -0.24 (Probable NON-ANTIGEN) |
| XP_807600.1 | Hypothetical protein | KICHVVFFR | 0.82 (Probable ANTIGEN) |
| PWV16110.1 | Hypothetical proteinC3747_25g140 | RTYHMIWNR | 0.30 (Probable NON-ANTIGEN) |
| PWV13412.1 | Putative SNF2 DNA repair protein | RVFFWKVQR | 0.04 (Probable NON-ANTIGEN) |
| XP_817046.1 | Hypothetical protein | MTFVFEARR | 0.06 (Probable NON-ANTIGEN) |
| PWV08907.1 | Chaperonin HSP60, mitochondria precursor | KMWQRTFTR | -0.14 (Probable NON-ANTIGEN) |
| PWV16110.1 | Hypothetical protein C3747_25g140 | RLWRWRCMR | 1.62 (Probable ANTIGEN). |
| ESS67427.1 | Phosphatase -like protein | RINFCFYVR | 1.98 (Probable ANTIGEN). |
| PWV08907.1 | Hypothetical protein C3747_84g29 | RQRAILMYR | -0.45 (Probable NON-ANTIGEN). |
| XP_811814.1 | Hypothetical protein | KMRVWRHQR | -0.36 (Probable NON-ANTIGEN). |
| ESS67427.1 | Hypothetical protein TCDM_03859 | RMNLITWHR | -0.19 (Probable NON-ANTIGEN |
| XP_805332.1 | Hypothetical protein | FHDQTIFCL | -0.44 (Probable NON-ANTIGEN). |
| XP_811822.1 | Leucine carboxyl methyltransferase | MHDHYCFVL | 0.29 (Probable NON-ANTIGEN) |
| XP_818330.1 | Hypothetical protein | IPMRRRRSL | -0.58 (Probable NON-ANTIGEN). |
| XP_807567.1 | Hypothetical protein | FHFCITFCL | 2.28 (Probable ANTIGEN) |
|  |  |  | Mean=0.58 |

**Table F5:** CTL (CD8+) epitopes used in our study.

| **Sl. No.** | **Protein ID** | **Top CTL Epitopes** | **VaxiJen Score** |
| --- | --- | --- | --- |
| 1 | XP_813527.1 (DGF-1) | DAALLGGDY | 2.09 (Probable ANTIGEN) |
| 2 | XP_809835.1 (subtilisin-like serine peptidase) | GVDFDSCFF | 1.84 (Probable ANTIGEN) |
| 3 | XP_806816.1 (DNAJ Chaperone protein) | KTGRNGDMY | 1.81 (Probable ANTIGEN) |
| 4 | XP_809166.1 (MASP) | STDDHATGS | 1.75 (Probable ANTIGEN) |
| 5 | XP_816522.1 (Mucin TcMUCII) | GTDGVTGTT | 1.48 (Probable ANTIGEN) |
| 6 | XP_818708.1 (Trans-sialidase) | SSDADPTVV | 1.03 (Probable ANTIGEN) |
| 7 | XP_815016.1 (Surface Protein) | LLVLAALTY | 0.94 (Probable ANTIGEN) |
| 8 | XP_821916.1 (hypothetical protein) | YTCGTSCAV | 0.75 (Probable ANTIGEN) |
|  |  |  | **Mean=1.46** |

**Reference:**

1. Michel-Todó, Lucas. *et al.* In silico design of an epitope-based vaccine ensemble for Chagas disease. *Frontiers in immunology* **10**, 2698 (2019).
