# Supplementary material for: Identification of vaccine targets in pathogens and design of a vaccine using computational approaches
Source: Sci Rep. 2021 Sep 2;11:17626. doi: 10.1038/s41598-021-96863-x (PMC8413327; doi:10.1038/s41598-021-96863-x)
Supplement: Supplementary file 3 — Supplementary Information 3. [file 41598_2021_96863_MOESM3_ESM.docx]

# Identification of vaccine targets in pathogens and design of a vaccine using computational approaches

Kamal Rawal^#1^, Robin Sinha^1^, Bilal Ahmed Abbasi^1^, Amit Chaudhary^1^, Swarsat Kaushik Nath^1^, Priya Kumari^1^, Preeti P.^1^, Devansh Saraf^1^, Shachee Singh^1^, Kartik Mishra^1^, Pranjay Gupta^1^, Astha Mishra^1^, Trapti Sharma^1^, Srijanee Gupta^1^, Prashant Singh^1^, Shriya Sood^1^, Preeti Subramani^1,^ Aman Kumar Dubey^1^, Ulrich Strych^2^, Peter J. Hotez^2, 3^, Maria Elena Bottazzi^2, 3^

1. Amity Institute of Biotechnology, Amity University Uttar Pradesh, India.
2. Texas Children’s Hospital Center for Vaccine Development, Departments of Pediatrics
   and Molecular Virology and Microbiology, National School of Tropical Medicine,
   Baylor College of Medicine, Houston, TX, USA.
3. Department of Biology, Baylor University, Waco, Texas, USA.

#Corresponding Author

Email ID: kamal.rawal@gmail.com

Centre for Computational Biology and Bioinformatics, AIB

Amity University, Noida.

| **Supplementary Tables** | | |
| --- | --- | --- |
| **Title** | **Sub-Title** | **Page Numbers** |
| **Supplementary Table 1** | Tools used for extraction of features along with their cut-off values and justification | **4-7** |
| **Supplementary Table 2** | Vax-Elan Standalone Docker | **See excel sheet [Supplementary Table 2.xlsx]** |
| **Supplementary Table 3** | Vax-Elan output (Binary file) | **See excel sheet [Supplementary Table 3.xlsx]** |
| **Supplementary Table 4** | Row wise sum file | **See excel sheet [Supplementary Table 4.xlsx]** |
| **Supplementary Table 5** | Top 10 unique protein list | **See excel sheet [Supplementary Table 5.xlsx]** |
| **Supplementary Table 6** | Strategy 2 pre-computed thresholds for each property | **8** |
| **Supplementary Table 7** | Different pathogens used for the identification of key vaccine candidates. | **8-9** |
| **Supplementary Table 8** | Positive and negative sequences extracted from bacteria, protozoa, fungi, and viruses. | **9** |
| **Supplementary Table 9** | Set of desirable properties | **9-12** |
| **Supplementary Table 10** | **a.** Different strains of *T. cruzi* downloaded from NCBI for evolutionary and conservancy analysis.  **b.** Different species of Trypanosoma downloaded from NCBI for evolutionary and conservancy analysis | **See excel sheet [Supplementary Table 10.xlsx]** |
| **Supplementary Table-11** | Distribution of properties in *T. cruzi* CL Brener | **See excel sheet [Supplementary Table 11.xlsx]** |
| **Supplementary Table-12** | Evidence extracted from literature for the shortlisted proteins | **12-13** |
| **Supplementary Table 13** | Percentage of Identity in 8 prioritized proteins across different strains of *T. cruzi*  Percentage of Similarity in 8 prioritized proteins across different strains of *T. cruzi* | **See excel sheet [Supplementary Table 13.xlsx]** |
| **Supplementary Table 14** | **a.** Estimates of evolutionary divergence between sequences from different species of Trypanosoma for different proteins  **b.** Estimates of evolutionary divergence between sequences of different strains of *Trypanosoma cruzi* for different proteins  **c.** Estimates of Evolutionary Divergence between Sequences | **See excel sheet [Supplementary Table 14.xlsx]** |
| **Supplementary Table 15** | Antigenicity score of epitopes | **See excel sheet [Supplementary Table 15.xlsx]** |
| **Supplementary Table 16** | Conservancy analysis of epitopes against different strains of *Trypanosoma cruzi* | **See excel sheet [Supplementary Table 16.xlsx]** |

**Supplementary Table 1:** Tools used for extraction of features along with their cut-off values and justification

| **S. No.** | **Features** | **Tool** | **Cut-off** | **References** | **Justification/Reason** |
| --- | --- | --- | --- | --- | --- |
| **1** | Proteins with less number of Trans-membrane Helices | TmPred  TMHMM  HMMtop | <=1 | Monterrubio et al., 2015  Naz et al., 2019  Solanki et al., 2018 | **See doc file Supplementary Table A.doc** |
| **2** | Non-homology with Human | BLAST with Human Proteome | e-value:10e-5,  Identity>30%,  Query coverage>= 70% | Pearson et al.,2013 | **See doc file Supplementary Table A.doc** |
| **3** | Stability (Instability Index Value) | ProtParam | <40 | Solanki et al., 2018 | **See doc file Supplementary Table A.doc** |
| **4** | Non-Allergen | Blastp with Aller | e-value:10e-5,  Identity>30% | Pearson et al.,2013 | **See doc file Supplementary Table A.doc** |
| **5** | Adhesion Prediction | FungalRv | >=-1.2 | Monterrubio et al., 2015 | **See doc file Supplementary Table A.doc** |
| **6** | Essential genes prediction | DEG Database | e-value:10e-5,  Identity>30%,  Query coverage>= 70% | Solanki et al., 2018 | **See doc file Supplementary Table A.doc** |
| **7** | Virulence Factor | Blastp with VFDB | e-value:10e-5,  Identity>30%, | Solanki et al., 2018 | **See doc file Supplementary Table A.doc** |
| **8** | Molecular Weight | ProtParam | <110 kDa | Naz et al., 2019 | **See doc file Supplementary Table A.doc** |
| **9** | Secretory/Non-secretory protein | Signalp (dvalue) | >=0.5 | Liebenberg et al., 2012 | **See doc file Supplementary Table A.doc** |
| **10** | Non-Bacterial Pathogen/BLAST with Gutflora. | Blastp with GutfloraDB | e-value:10e-5,  Identity>30%,  Query coverage>= 70% | Naz et al., 2019  Pearson et al.,2013 | **See doc file Supplementary Table A.doc** |
| **11** | Sub-cellular Localization | Targetp | >=0.8 | Goodswen et al., 2014 | **See doc file Supplementary Table A.doc** |
| **12** | MHC Class-1 binding (Number of High Binders) | NetMHC | >=4.9 | Schroeder et al., 2011 | **See doc file Supplementary Table A.doc** |
| **13** | MHC Class-1 binding (Number of Weak Binders) | NetMHC | >=5.05 | Schroeder et al., 2011 | **See doc file Supplementary Table A.doc** |
| **14** | Number of Cleavage Sites | NetChop | >=110 | Dhanda et al., 2017 | **See doc file Supplementary Table A.doc** |
| **15** | Number of Peptides | NetMHC | <500 | Schroeder et al., 2011 | **See doc file Supplementary Table A.doc** |
| **16** | Number of Amino Acids | NetChop | <500 | Dhanda et al., 2017 | **See doc file Supplementary Table A.doc** |
| **17** | Cytotoxic T Lymphocytes (CTL Epitope Prediction) (Number of MHC Ligands) | NetCTL | <7.5 | Solanki et al., 2018 | **See doc file Supplementary Table A.doc** |
| **18** | Antigenicity | Vaxijen | >0.4 | Monterrubio et al., 2015 | **See doc file Supplementary Table A.doc** |
| **19** | Subcellular Localization (Gram Positive) | Psortb | >9.5 | Muruato et al., 2017 | **See doc file Supplementary Table A.doc** |
| **20** | Subcellular Localization | Psortb | >9.5 | Muruato et al., 2017 | **See doc file Supplementary Table A.doc** |
| **21** | MHC Class-1 binding prediction | IEDB  (HLA02*01) | >50nM | Schroeder et al., 2011 | **See doc file Supplementary Table A.doc** |
| **22** | Subcellular Localization (Gram Positive) | Psortb | Cell wall  Extracellular | Naz et al., 2019  Muruato et al., 2017  Solanki et al., 2018 | **See doc file Supplementary Table A.doc** |
| **23** | Subcellular Localization | Psortb | Outer Membrane, Extracellular and periplasmic | Naz et al., 2019 | **See doc file Supplementary Table A.doc** |
| **24** | Subcellular Localization | Wolf Psort | Extracellular or Plasma membrane | Watanabe et al., 2021 | **See doc file Supplementary Table A.doc** |

**References:**

1. Monterrubio-López, G. P., & Ribas-Aparicio, R. M. (2015). Identification of novel potential vaccine candidates against tuberculosis based on reverse vaccinology. *BioMed research international*, *2015*.
2. Naz, K., Naz, A., Ashraf, S. T., Rizwan, M., Ahmad, J., Baumbach, J., & Ali, A. (2019). PanRV: Pangenome-reverse vaccinology approach for identifications of potential vaccine candidates in microbial pangenome. *BMC bioinformatics*, *20*(1), 1-10.
3. Muruato, L. A., Tapia, D., Hatcher, C. L., Kalita, M., Brett, P. J., Gregory, A. E., ... & Torres, A. G. (2017). Use of reverse vaccinology in the design and construction of nanoglycoconjugate vaccines against Burkholderia pseudomallei. *Clinical and Vaccine Immunology*, *24*(11).
4. Solanki, V., & Tiwari, V. (2018). Subtractive proteomics to identify novel drug targets and reverse vaccinology for the development of chimeric vaccine against Acinetobacter baumannii. *Scientific reports*, *8*(1), 1-19.
5. Goodswen, S. J., Kennedy, P. J., & Ellis, J. T. (2014). Vacceed: a high-throughput in silico vaccine candidate discovery pipeline for eukaryotic pathogens based on reverse vaccinology. *Bioinformatics*, *30*(16), 2381-2383.
6. Schroeder, J., & Aebischer, T. (2011). Vaccines for leishmaniasis: from proteome to vaccine candidates. *Human vaccines*, *7*(sup1), 10-15.
7. Dhanda, S.K., Usmani, S.S., Agrawal, P., Nagpal, G., Gautam, A. and Raghava, G.P., 2017. Novel in silico tools for designing peptide-based subunit vaccines and immunotherapeutics. Briefings in Bioinformatics, 18(3), pp.467-478
8. Liebenberg, J., Pretorius, A., Faber, F. E., Collins, N. E., Allsopp, B. A., & Van Kleef, M. (2012). Identification of Ehrlichia ruminantium proteins that activate cellular immune responses using a reverse vaccinology strategy. *Veterinary immunology and immunopathology*, *145*(1-2), 340-349.
9. Pearson, W. R. (2013). An introduction to sequence similarity (“homology”) searching. *Current Protocols in Bioinformatics*, *Chapter 3*(SUPPL.42).
10. Watanabe Y, Zenke K, Itoh N, Yoshinaga T. Functional analysis of the proteases overexpressed during the invasive and parasitic stages of Cryptocaryon irritans and their potential as vaccine antigens. Aquaculture. 2021 Jul 15; 540:736657.

**Supplementary Table 6:** Strategy 2 pre-computed thresholds for each property


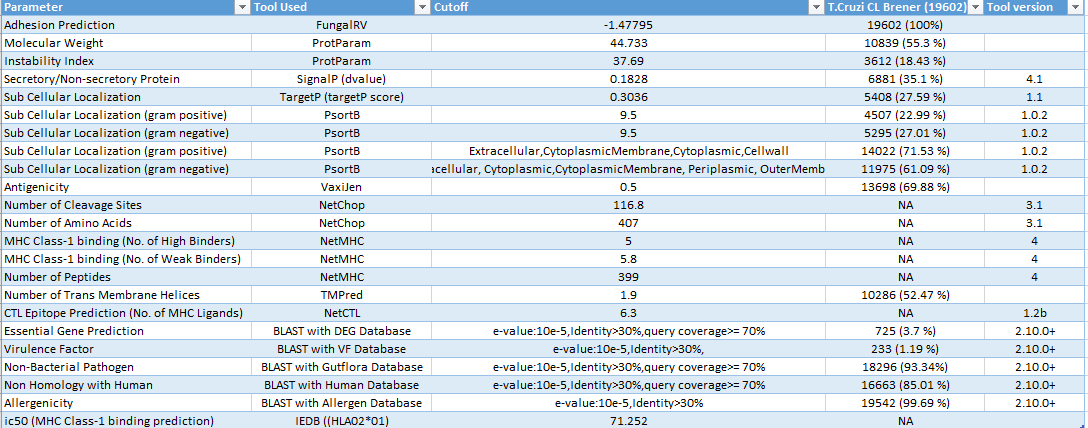


**Supplementary Table 7:** Different pathogens used for the identification of key vaccine candidates.

| **Bacteria** |
| --- |
| 1. *Bordetella pertussis* (strain Tohama I / ATCC BAA-589 / NCTC 13251) 2. *Corynebacterium diphtheriae* (strain ATCC 700971 / NCTC 13129 / Biotype gravis) 3. *Mycoplasma pneumoniae* (strain ATCC 29342 / M129) 4. *Neisseria gonorrhoeae* (strain ATCC 700825 / FA 1090) 5. *Salmonella typhi* (strain: CT18) 6. *Treponema pallidum* (strain Nichols) 7. *Clostridium botulinum* (strain Hall / ATCC 3502 / NCTC 13319 / Type A) (Strain: Hall / ATCC 3502 / NCTC 13319 / Type A [Sanger]) |
| **Fungi** |
| 1. *Aspergillus fumigatus* (strain ATCC MYA-4609 / Af293 / CBS 101355 / FGSC A1100) 2. *Blastomyces dermatitidis* (strain: ATCC 26199) 3. *Candida albicans* (strain SC5314 / ATCC MYA-2876) 4. *Histoplasma capsulatum* (strain G186AR / H82 / ATCC MYA-2454 / RMSCC 2432) (Darling's disease fungus) |
| **Protozoa** |
| 1. *Leishmania donovani* (strain: FDAARGOS_361) 2. *Plasmodium falciparum* (isolate 3D7) 3. *Plasmodium vivax* (strain Salvador I) 4. *Entamoeba histolytica* (Strain: ATCC 30459 / HM-1:IMSS) 5. *Toxoplasma gondii* (strain ATCC 50861 / VEG) |
| **Viruses** |
| 1. Human Papillomavirus (Human papillomavirus type 1a) 2. Influenza A virus (strain A/Puerto Rico/8/1934 H1N1) 3. Measles virus (strain Ichinose-B95a) (MeV) (*Subacute sclerose panencephalitis* virus) 4. Mumps Virus (strain Miyahara vaccine) (MuV) 5. Nipah Virus |

**Supplementary Table 8:** Information regarding positive and negative sequences extracted from bacteria, protozoa, fungi, and viruses.

| **Organism** | **Positive** | **Negative** | **Source** |
| --- | --- | --- | --- |
| Bacteria | 670 | 677 | Protegen and UniProt |
| Protozoa | 175 | 293 | Protegen and UniProt |
| Fungi | 139 | 138 | Protegen and UniProt |
| Viruses | 418 | 419 | Protegen and UniProt |

**Defining a Potential Vaccine Candidate (PVC) and its properties.**

Vaccines are the biological agent which have the potential to invoke an immune response. In the past century, vaccines have been successful in decreasing morbidity and mortality rates^1^. Here, we present the set of desirable properties for protein derived vaccines, which have been investigated by different scientific groups to characterise vaccine candidates and antigens. These properties were used in our study for evaluating and identifying potential vaccine candidates.

**Supplementary Table 9:** Set of desirable properties.

| **S. No.** | **Features** | **Tool** | **Cut-off** | **REFERENCES** |
| --- | --- | --- | --- | --- |
| **1** | Proteins with less number of Trans-membrane Helices | TmPred  TMHMM  HMMtop | <=1 (L), <=1.9 (C) | Monterrubio et al., 2015  Naz et al., 2019  Solanki et al., 2018 |
| **2** | Non-homology with Human | BLAST with Human Proteome | e-value:10e-5,  Identity>30%,  Query coverage>= 70% | Pearson et al.,2013 |
| **3** | Stability (Instability Index Value) | ProtParam | <40 (L), <37.69 (C) | Solanki et al., 2018 |
| **4** | Non-Allergen | Blastp with AllerBase | e-value:10e-5,  Identity>30% | Pearson et al.,2013 |
| **5** | Adhesion Prediction | FungalRv | >=-1.2(L), -1.47(C) | Monterrubio et al., 2015 |
| **6** | Essential genes prediction | DEG Database | e-value:10e-5,  Identity>30%,  Query coverage>= 70% | Solanki et al., 2018 |
| **7** | Virulence Factor | Blastp with VFDB | e-value:10e-5,  Identity>30%, | Solanki et al., 2018 |
| **8** | Molecular Weight | ProtParam | <110 kDa (L),  <44.73 kDa (C) | Naz et al., 2019 |
| **9** | Secretory/Non-secretory protein | Signalp (dvalue) | >=0.5(L), >=0.18 (C) | Liebenberg et al., 2012 |
| **10** | Non-Bacterial Pathogen/BLAST with Gutflora. | Blastp with GutfloraDB | e-value:10e-5,  Identity>30%,  Query coverage>= 70% | Naz et al., 2019 |
| **11** | Sub-cellular Localization | Targetp | >=0.8(L), >=0.30 (C)  Secretory protein | Goodswen et al., 2014 |
| **12** | MHC Class-1 binding (Number of High Binders) | NetMHC | >=4.9, >=5 (C) | Schroeder et al., 2011 |
| **13** | MHC Class-1 binding (Number of Weak Binders) ** | NetMHC | >=5.05, >=5.8 (C) | Schroeder et al., 2011 |
| **14** | Number of Cleavage Sites** | NetChop | >=110, > 116.8 (C) | Dhanda et al., 2017 |
| **15** | Number of Peptides** | NetMHC | <500, <399 (C) | Schroeder et al., 2011 |
| **16** | Number of Amino Acids** | NetChop | <500, < 407 (C) | Dhanda et al., 2017 |
| **17** | Cytotoxic T Lymphocytes (CTL Epitope Prediction) (Number of MHC Ligands) ** | NetCTL | <7.5, <6.3 (C) | Solanki et al., 2018 |
| **18** | Antigenicity | Vaxijen | >0.4 | Monterrubio et al., 2015 |
| **19** | Subcellular Localization (Gram Positive) | Psortb | >9.5 | Muruato et al., 2017 |
| **20** | Subcellular Localization | Psortb | >9.5 | Muruato et al., 2017 |
| **21** | MHC Class-1 binding prediction | IEDB  (HLA02*01) | >50 nM, >71.25 nM (C) | Schroeder et al., 2011 |
| **22** | Subcellular Localization (Gram Positive) | Psortb | Cell wall  Extracellular | Naz et al., 2019  Muruato et al., 2017  Solanki et al., 2018 |
| **23** | Subcellular Localization | Psortb | Outer Membrane, Extracellular and periplasmic | Naz et al., 2019 |
| **24** | Subcellular Localization | Wolf Psort | Extracellular and Plasma Membrane | Watanabe et al., 2021 |

(L) Represents cut-offs derived from the literature or the default threshold.

(C) Represents cut-off determined by the statistical & computational methods (developed in-house by comparing distributions of properties in positive and negative datasets).

**- Denotes properties which were found to be statistically not significant when evaluated through computational means.

**Reference:**

1. Monterrubio-López, G. P., & Ribas-Aparicio, R. M. (2015). Identification of novel potential vaccine candidates against tuberculosis based on reverse vaccinology. *BioMed research international*, *2015*.
2. Naz, K., Naz, A., Ashraf, S. T., Rizwan, M., Ahmad, J., Baumbach, J., & Ali, A. (2019). PanRV: Pangenome-reverse vaccinology approach for identifications of potential vaccine candidates in microbial pangenome. *BMC bioinformatics*, *20*(1), 1-10.
3. Muruato, L. A., Tapia, D., Hatcher, C. L., Kalita, M., Brett, P. J., Gregory, A. E., ... & Torres, A. G. (2017). Use of reverse vaccinology in the design and construction of nanoglycoconjugate vaccines against Burkholderia pseudomallei. *Clinical and Vaccine Immunology*, *24*(11).
4. Solanki, V., & Tiwari, V. (2018). Subtractive proteomics to identify novel drug targets and reverse vaccinology for the development of chimeric vaccine against Acinetobacter baumannii. *Scientific reports*, *8*(1), 1-19.
5. Goodswen, S. J., Kennedy, P. J., & Ellis, J. T. (2014). Vacceed: a high-throughput in silico vaccine candidate discovery pipeline for eukaryotic pathogens based on reverse vaccinology. *Bioinformatics*, *30*(16), 2381-2383.
6. Schroeder, J., & Aebischer, T. (2011). Vaccines for leishmaniasis: from proteome to vaccine candidates. *Human vaccines*, *7*(sup1), 10-15.
7. Dhanda, S.K., Usmani, S.S., Agrawal, P., Nagpal, G., Gautam, A. and Raghava, G.P., 2017. Novel in silico tools for designing peptide-based subunit vaccines and immunotherapeutics. Briefings in Bioinformatics, 18(3), pp.467-478
8. Liebenberg, J., Pretorius, A., Faber, F. E., Collins, N. E., Allsopp, B. A., & Van Kleef, M. (2012). Identification of Ehrlichia ruminantium proteins that activate cellular immune responses using a reverse vaccinology strategy. *Veterinary immunology and immunopathology*, *145*(1-2), 340-349.
9. Pearson, W. R. (2013). An introduction to sequence similarity (“homology”) searching. *Current Protocols in Bioinformatics*, *Chapter 3*(SUPPL.42).
10. Watanabe Y, Zenke K, Itoh N, Yoshinaga T. Functional analysis of the proteases overexpressed during the invasive and parasitic stages of Cryptocaryon irritans and their potential as vaccine antigens. Aquaculture. 2021 Jul 15; 540:736657.

We used literature mining to find out the experimental information against shortlisted proteins. In this exercise, we have collected pieces of evidences from literature databases (i.e., Google-scholar & PubMed) that have reported that predicted proteins have been evaluated by other research groups in the context of vaccines and antigens.

**Supplementary Table 12:** Evidence extracted from literature for the shortlisted proteins.

| **S. No** | **Proteins used in vaccine development**  **Keywords used** | **No. of Google scholar hits** | **Evidence(s)** |
| --- | --- | --- | --- |
| **1** | XP_813527.1 (DGF-1);  DGF-1 Vaccine | 83 | No Significant Results |
| **2** | XP_809835.1 (Subtilisin  -like serine peptidase); Subtilisin-like serine peptidase Vaccine | 27 | PMID:  21718740,  24100558,  22919676 |
| **3** | XP_806816.1 (DNAJ Chaperone protein);  DNAJ Chaperone protein Vaccine | 4,110 | PMID:  22539989,  17307260 |
| **4** | XP_809166.1 (MASP); MASP Vaccine | 2,930 | PMID: 24793944 |
| **5** | XP_816522.1 (Mucin TcMUCII);  Mucin TcMUCII Vaccine | 35 | PMID: 20957201 |
| **6** | XP_818708.1(Trans-sialidase);  Trans-sialidase Vaccine | 2,390 | PMID:  18403070,  25625671,  16972758 |
| **7** | XP_815016.1 (Surface Protein);  Surface Protein Vaccine | 18,00,000 | PMID: 16626494 |
| **8** | XP_821916.1 (hypothetical)  Matched with Regulator of Sigma E Protease. | 43,110 | No Significant Results. |
