# Supplementary material for: Identification of vaccine targets in pathogens and design of a vaccine using computational approaches
Source: Sci Rep. 2021 Sep 2;11:17626. doi: 10.1038/s41598-021-96863-x (PMC8413327; doi:10.1038/s41598-021-96863-x)
Supplement: Supplementary file 18 — Supplementary Information 18. [file 41598_2021_96863_MOESM18_ESM.docx]

**Supplementary Table A:** Evidences of parameters used by Vax-Elan

**Trans-membrane helices**

Serruto et al.,2004 reported the main cause of failed cloning and expression of 250 out of 600 vaccine candidates from *Neisseria meningitidis*B due to the presence of more than one transmembrane spanning region (TM) (Serruto et al., 2004).

Serruto, D., Rappuoli, R., & Pizza, M. (2004). Meningococcus B: from genome to vaccine. *Genomics, Proteomics and Vaccines*, 185-201.

**Non-homology with human**

Baseer et al., 2017 used BLASTp (sequences identity ≤35% and E-value cut-off-10^−4^) to filter out the host non-homologous proteins. Removal of host homologous sequences is crucial as such proteins generate cross-reactivity with the host proteins giving rise to adverse autoimmune responses (Baseer et al., 2017).

Baseer, S., Ahmad, S., Ranaghan, K. E., & Azam, S. S. (2017). Towards a peptide-based vaccine against Shigella sonnei: A subtractive reverse vaccinology-based approach. *Biologicals*, *50*, 87-99.

**Stability (Instability index value)**

Vishnu et al., 2017 analysed physicochemical properties of the designed protein and result suggested that the protein classified as stable with an instability index (II) of 24.67 (II < 40 is considered as stable) (Vishnu et al., 2017).

Vishnu, U. S., Sankarasubramanian, J., Gunasekaran, P., & Rajendhran, J. (2017). Identification of potential antigens from non-classically secreted proteins and designing novel multitope peptide vaccine candidate against Brucella melitensis through reverse vaccinology and immunoinformatics approach. *Infection, Genetics and Evolution*, *55*, 151-158.

**Non-allergen**

Baseer et al., 2017 used BLASTp (sequences identity ≤35% and E-value cut-off-10^−4^) to filter out the host non-homologous proteins. Removal of host homologous sequences is crucial as such proteins generate cross-reactivity with the host proteins giving rise to adverse autoimmune responses (Baseer et al., 2017).

Baseer, S., Ahmad, S., Ranaghan, K. E., & Azam, S. S. (2017). Towards a peptide-based vaccine against Shigella sonnei: A subtractive reverse vaccinology-based approach. *Biologicals*, *50*, 87-99.

**Adhesin probability**

Monterrubio et al., 2015 were performed *M. tuberculosis* H37Rv proteome analysis using NERVE and they found 331 proteins out of 3989 proteins of *M. tuberculosis* H37Rv proteome. Adhesion probability was ≥50% selected for the making good vaccine candidates (Monterrubio et al., 2015).

Monterrubio-López, G. P., & Ribas-Aparicio, R. M. (2015). Identification of novel potential vaccine candidates against tuberculosis based on reverse vaccinology. *BioMed research international*, *2015*.

**Essential genes prediction**

Baseer et al., 2017 used BLASTp (sequences identity ≤35% and E-value cut-off-10^−4^) to filter out the host non-homologous proteins. Removal of host homologous sequences is crucial as such proteins generate cross-reactivity with the host proteins giving rise to adverse autoimmune responses (Baseer et al., 2017).

Baseer, S., Ahmad, S., Ranaghan, K. E., & Azam, S. S. (2017). Towards a peptide-based vaccine against Shigella sonnei: A subtractive reverse vaccinology-based approach. *Biologicals*, *50*, 87-99.

**Virulence factor**

Baseer et al., 2017 used BLASTp (sequences identity ≤35% and E-value cut-off-10^−4^) to filter out the host non-homologous proteins. Removal of host homologous sequences is crucial as such proteins generate cross-reactivity with the host proteins giving rise to adverse autoimmune responses (Baseer et al., 2017).

Baseer, S., Ahmad, S., Ranaghan, K. E., & Azam, S. S. (2017). Towards a peptide-based vaccine against Shigella sonnei: A subtractive reverse vaccinology-based approach. *Biologicals*, *50*, 87-99.

**Molecular weight**

For the identifications of potential vaccine candidates in microbial pangenome Naz et al., 2019 used proteins having < 110 kDa molecular weight in the data set (proteome). As small (low mol. weight) proteins can easily be purified and handled effectively during vaccine development (Naz et al., 2019).

Naz, K., Naz, A., Ashraf, S. T., Rizwan, M., Ahmad, J., Baumbach, J., & Ali, A. (2019). PanRV: Pangenome-reverse vaccinology approach for identifications of potential vaccine candidates in microbial pangenome. *BMC bioinformatics*, *20*(1), 1-10.

**Secretory/Non-secretory protein**

In Genome-wide analysis of Excretory/Secretory proteins, Gahoi et al., 2017 found 17,876 non secretory proteins by using SignalP and Maritz-Olivier et al. 2012 identified 176 membrane-associated and 86 secreted soluble proteins (Maritz et al., 2012).

Gahoi, S., & Gautam, B. (2017). Genome-wide analysis of Excretory/Secretory proteins in root-knot nematode, Meloidogyne incognita provides potential targets for parasite control. Computational biology and chemistry, 67, 225-233.

Maritz-Olivier, C., van Zyl, W., & Stutzer, C. (2012). A systematic, functional genomics, and reverse vaccinology approach to the identification of vaccine candidates in the cattle tick, Rhipicephalus microplus. *Ticks and tick-borne diseases*, *3*(3), 179-187.

**Non-bacterial pathogen/BLAST with gut flora**

Baseer et al., 2017 used BLASTp (sequences identity ≤35% and E-value cut-off-10^−4^) to filter out the host non-homologous proteins. Removal of host homologous sequences is crucial as such proteins generate cross-reactivity with the host proteins giving rise to adverse autoimmune responses (Baseer et al., 2017).

Baseer, S., Ahmad, S., Ranaghan, K. E., & Azam, S. S. (2017). Towards a peptide-based vaccine against Shigella sonnei: A subtractive reverse vaccinology-based approach. *Biologicals*, *50*, 87-99.

**Subcellular localisation (TargetP)**

TargetP provides a ‘‘reliability coefficient’’, RC, which is a measure of how confident TargetP is in each prediction. The RC ranges from 1 (very reliable prediction; virtually no false positives detected in the TargetP test set) to 5 (not reliable prediction; many false positives detected)—

The ‘‘reliability class’’ (RC) value associated with each prediction signifies how confident TargetP is in each prediction. The RC is based on the difference between the highest and the second highest TargetP output scores: if this difference is larger than 0.8, then RC ¼ 1; if it is between 0.6 and 0.8, then RC ¼ 2, and so on. An RC value of 1 means that the prediction belongs to the most reliable class of predictions, and 5 that it belongs to the least reliable class (Emanuelsson et al., 2007).

Interestingly, a study demonstrated that IMP1 was found to localise to the membrane of N. caninum tachyzoites and speculated that this membrane targeting was instigated by N-myristoylation and palmitoylation (Goodswen et al., 2017).

Goodswen, S. J., Kennedy, P. J., & Ellis, J. T. (2017). On the application of reverse vaccinology to parasitic diseases: a perspective on feature selection and ranking of vaccine candidates. International journal for parasitology, 47(12), 779-790.

Emanuelsson, O., Brunak, S., Von Heijne, G., & Nielsen, H. (2007). Locating proteins in the cell using TargetP, SignalP and related tools. *Nature protocols*, *2*(4), 953.

**MHC Class-1 binding (Number of high binders & Number of weak binders)**

Schroeder et al., 2011 used NetMHC for the epitope prediction and they found Kinetoplastid membrane protein-11 (KMP-11) generates a cluster of predicted binding peptides in the N-terminal region of the protein, with one classified as strong binder (threshold affinity ≤50 nM) starting at position 2 and a number of weakly binding peptides (threshold affinity ≤500 nM) in the region 2–21(Schroeder et al., 2011).

Schroeder, J., & Aebischer, T. (2011). Vaccines for leishmaniasis: from proteome to vaccine candidates. *Human vaccines*, *7*(sup1), 10-15.

**Number of cleavage sites >116.8**

**Number of peptides (**Not used in current version of Vax-Elan**)**

**Number of amino acids (**Not used in current version of Vax-Elan**)**

**Cytotoxic T Lymphocytes (CTL Epitope Prediction) (Number of MHC Ligands)**

Solanki et al., 2018 used NetCTL server to predict MHC class I epitopes using threshold value 0.75. Result suggests that 17 epitopes in B0VMD0 proteins while 27 epitopes in B0VUZ6 were identified (Solanki et al., 2018).

Solanki, V., & Tiwari, V. (2018). Subtractive proteomics to identify novel drug targets and reverse vaccinology for the development of chimeric vaccine against Acinetobacter baumannii. Scientific reports, 8(1), 1-19.

**Antigenicity**

To predict the antigenic property of all the selected proteins was determined using VaxiJen web server and threshold of 0.5 was considered as the potent antigenicity. Result showed that B0VUZ6 and B0VMD0 were identified as the most potent antigenic protein having a maximum total prediction score of 0.5541 and 0.5789 respectively (Solanki et al., 2018).

Solanki, V., & Tiwari, V. (2018). Subtractive proteomics to identify novel drug targets and reverse vaccinology for the development of chimeric vaccine against Acinetobacter baumannii. *Scientific reports*, *8*(1), 1-19.

**Subcellular localization**

Baseer et al., 2017 used PSORTb to screen for outer membrane and extracellular (e.g., secreted) proteins, selecting proteins with prediction scores of >9.5. They found 26 outer membranes, 5 extracellular, 185 unknown, 41 periplasmic, 364 inner membrane and 674 cytoplasmic proteins (Baseer et al., 2017).

Baseer, S., Ahmad, S., Ranaghan, K. E., & Azam, S. S. (2017). Towards a peptide-based vaccine against Shigella sonnei: A subtractive reverse vaccinology-based approach. *Biologicals*, *50*, 87-99.

**MHC Class-1 binding prediction**

Andreatta et al., 2016 generated prediction models for all MHC class I molecules with at least 20 data points, of which at least four have IC50 affinity <500 nM, resulting in a set of 118 MHC class I (86 human, six murine, 26 primate) alleles (Andreatta et al., 2016).

Andreatta, Massimo, and Morten Nielsen. “Gapped sequence alignment using artificial neural networks: application to the MHC class I system.” Bioinformatics (Oxford, England) vol. 32,4 (2016): 511-7. doi:10.1093/bioinformatics/btv639

**Subcellular localization (**WoLF PSORT**)**

Watanabe et al., 2021 analysed target protease coding cDNA determined from trophonts obtained from RACE PCR using WoLF PSORT suggested that sp1, sp2, and cp1 are extracellular proteins, while cp2 is an extracellular or plasma membrane protein.

Watanabe Y, Zenke K, Itoh N, Yoshinaga T. Functional analysis of the proteases overexpressed during the invasive and parasitic stages of Cryptocaryon irritans and their potential as vaccine antigens. Aquaculture. 2021 Jul 15;540:736657.
